# Further contributions to the Aleocharinae (Coleoptera, Staphylinidae) fauna of New Brunswick and Canada including descriptions of 27 new species

**DOI:** 10.3897/zookeys.573.7016

**Published:** 2016-03-24

**Authors:** Reginald P. Webster, Jan Klimaszewski, Caroline Bourdon, Jon D. Sweeney, Cory C. Hughes, Myriam Labrecque

**Affiliations:** 1 24 Mill Stream Drive, Charters Settlement, NB, Canada E3C 1X1; 2 Natural Resources Canada, Canadian Forest Service, Laurentian Forestry Centre, 1055 du P.E.P.S., P.O. Box 10380, Stn. Sainte-Foy, Québec, Quebec, Canada G1V 4C7; 3 Natural Resources Canada, Canadian Forest Service, Atlantic Forestry Centre, 1350 Regent St., P.O. Box 4000, Fredericton, NB, Canada E3B 5P7

**Keywords:** Taxonomy, ecology, rove beetles, Staphylinidae, Aleocharinae, new species, new records, New Brunswick, Canada

## Abstract

This paper treats the discovery of new species and new records of aleocharine beetles for the province of New Brunswick. We report here 27 species new to science, one new North American record, six new Canadian records, and 29 new provincial records. The following are the new species: *Acrotona brachyoptera* Klimaszewski & Webster, **sp. n.**, *Acrotona sphagnorum* Klimaszewski & Webster, **sp. n.**, Atheta (Dimetrota) alphacrenuliventris Klimaszewski & Webster, **sp. n**., Atheta (Dimetrota) chartersensis Klimaszewski & Webster, **sp. n.**, Atheta (Dimetrota) cranberriensis Klimaszewski & Webster, **sp. n.**, Atheta (Dimetrota) bubo Klimaszewski & Webster, **sp. n**., Atheta (Dimetrota) mcalpinei Klimaszewski & Webster, **sp. n.**, Atheta (Dimetrota) makepeacei Klimaszewski & Webster, **sp. n.**, Atheta (Dimetrota) giguereae Klimaszewski & Webster, **sp. n.**, Atheta (Dimetrota) petitcapensis Klimaszewski & Webster, **sp. n**., *Atheta* (*sensu lato*) *pseudoschistoglossa* Klimaszewski & Webster, **sp. n**., *Atheta* (*sensu lato*) *sphagnicola* Klimaszewski & Webster, **sp. n**., *Atheta* (*sensu lato*) *thujae* Klimaszewski & Webster, **sp. n**., Atheta (Pseudota) pseudoklagesi Klimaszewski & Webster, **sp. n**., *Philhygra atypicalis* Klimaszewski & Webster, **sp. n**., Schistoglossa (Schistoglossa) pelletieri Klimaszewski & Webster, **sp. n**., *Thamiaraea corverae* Klimaszewski & Webster, **sp. n**., *Thamiaraea claydeni* Klimaszewski & Webster, **sp. n**., *Pleurotobia bourdonae* Klimaszewski & Webster, **sp. n**., *Pleurotobia brunswickensis* Klimaszewski & Webster, **sp. n**., *Agaricomorpha vincenti* Klimaszewski & Webster, **sp. n**., Gyrophaena (Gyrophaena) aldersonae Klimaszewski & Webster, **sp. n**., *Oligota polyporicola* Klimaszewski & Webster, **sp. n**., *Oligota sevogle* Klimaszewski & Webster, **sp. n**., *Hylota cryptica* Klimaszewski & Webster, **sp. n**., *Oxypoda sunpokeana* Klimaszewski & Webster, **sp. n**., and *Phloeopora gilbertae* Klimaszewski & Webster, **sp. n.** The spermatheca of *Dinaraea curtipenis* Klimaszewski & Webster, *Dinaraea longipenis* Klimaszewski & Webster, and *Dinaraea subdepressa* (Bernhauer) are illustrated for the first time. Male specimens of *Mniusa odelli* Klimaszewski & Webster were confirmed and are illustrated. Color habitus images and black and white images of the median lobe of the aedeagus, the spermatheca, and tergite and sternite VIII are provided for all species. New or additional habitat data are provided for most of the species treated in this contribution.

## Introduction

Webster et al. (2012) reviewed and summarized the knowledge of the Aleocharinae known from New Brunswick to 2012, and newly recorded 28 species, bringing the total number of species known from the province to 215. Later, Klimaszewski et al. (2013b, 2014, and 2015b, c) added 19 species in the genera *Atheta*, *Clusiota*, *Dinaraea*, *Gnathusa*, *Mniusa*, *Ocyusa*, and *Mocyta* to the faunal list of New Brunswick as a result of new species descriptions and new records. During the last several years, the senior author accumulated material containing 27 species new to science, one new North American record, six new Canadian records, and 29 new provincial records from the province of New Brunswick. The purpose of this paper is to report on these new discoveries.

## Methods and conventions

Various methods were employed to collect the specimens reported in this study. Details are outlined in Webster et al. (2009, Appendix). Some specimens were collected from Lindgren funnel trap samples during a study to develop improved survey tools for the detection of invasive species of Cerambycidae. These traps are visually similar to tree trunks and are often effective for sampling species of Coleoptera that live in microhabitats associated with standing trees (Lindgren 1983). In many sites, equal numbers of traps were deployed in the canopy and 1 m high under trees. For details of the methods used to deploy Lindgren traps and for sample collection, see Webster et al. (2012) and Hughes et al. (2014). A description of the habitat was recorded for all specimens collected during this survey. Locality and habitat data are presented here as on the labels for each record. Two labels were used on many specimens (RWC), one that included the locality, collection date, and collector, and one with macro- and microhabitat data and collection method. Information is separated by a ‘//’ in the data for specimens where more than one label is present. Macro- and microhabitat information, as well as additional published data, is summarized and discussed in the natural history section for each species.

Most specimens were dissected to confirm their identity. The genital structures were dehydrated in absolute alcohol and mounted in Canada balsam on celluloid microslides and then pinned with the specimen from which they originated. Images of the entire body and the genital structures were taken using an image processing system (Nikon SMZ 1500 stereoscopic microscope; Nikon Digit-like Camera DXM 1200F, and Adobe Photoshop software).

Morphological terms used in species descrptions mainly follow those used by Seevers (1978), Ashe (2000), and Klimaszewski et al. (2011). The ventral side of the median lobe of the aedeagus is considered to be the side of the bulbus containing the foramen mediale, the entrance of the ductus ejaculatorius, and the adjacent ventral side of the tubus of the median lobe with internal sac and its structures (this part is referred to as the parameral side in some recent publications); the opposite side is referred to as the dorsal part. In the species descriptions, microsculpture refers to the surface of the upper forebody (head, pronotum and elytra).

**Distribution.** New provincial records are cited with current distribution in Canada and Alaska, using abbreviations for the state, provinces, and territories, and are indicated in **bold** under **Distribution in Canada and Alaska**. The following abbreviations are used in the text:

AB Alberta


AK Alaska


BC British Columbia


MB Manitoba


NB New Brunswick


NF & LB Newfoundland and Labrador*


NS Nova Scotia


NT Northwest Territories


NU Nunavut


ON Ontario


PE Prince Edward Island


QC Quebec


SK Saskatchewan


YT Yukon Territory


*Newfoundland and Labrador are each treated separately under the current Distribution in Canada and Alaska.

USA state abbreviations follow those of the US Postal Service. Acronyms of collections examined and referred to in this study are as follows:

AFC
 Natural Resources Canada, Canadian Forest Service - Atlantic Forestry Centre, Fredericton, New Brunswick, Canada 


CNC
 Canadian National Collection of Insects, Arachnids and Nematodes, Agriculture and Agri-Food Canada, Ottawa, Ontario, Canada 


FMNH
 The Field Museum, Chicago, Illinois, USA. 


LFC
 Natural Resources Canada, Canadian Forest Service - Laurentian Forestry Centre, Quebec, Quebec, Canada 


LUC
Lund University Collection, Lund, Sweden 


NBM
 New Brunswick Museum, Saint John, New Brunswick, Canada 


NSPM
 Nova Scotia Provincial Museum, Halifax, Nova Scotia, Canada 


RWC
 Reginald Webster Collection, Charters Settlement, New Brunswick, Canada 


## Results

We report here on 63 species of Aleocharinae: 27 species new to science, one new North American record, six new Canadian records, and 29 new provincial records. Specimens were collected from a variety of microhabitats, including mushrooms (15 species), moist sphagnum or other vegetation near streams or ponds (14 species), under sea wrack or cobblestones near streams (7 species), in moldy corncobs or compost (7 species), inside or near the entrance to nests, burrows, or homes of animals such as owls, marmots and beavers (6 species), in animal dung (2 species), and on or under the bark of logs (2 species). Lindgren 12-funnel traps collected 33 of the 63 species and provided the sole specimens for 13 of the species.

### Species accounts

#### Family Staphylinidae Latreille, 1806

##### Subfamily Aleocharinae Fleming, 1821

###### Tribe Aleocharini Fleming, 1821

####### Subtribe Aleocharina Fleming, 1821

######## Aleochara (Calochara) rubricalis

Taxon classificationAnimaliaColeopteraStaphylinidae

(Casey, 1911)

Figs 1–8

Aleochara (Calochara) rubricalis (For diagnosis, see Klimaszewski 1984)

######### Material examined.

**Canada, New Brunswick, Northumberland Co.**, Upper Graham Plains, 47.1001°N, 66.8154°W, 28.V-10.VI.2014, C. Alderson & V. Webster // Old black spruce forest, Lindgren funnel trap (1 ♂, RWC). **Restigouche Co.**, Dionne Brook P.N.A., 47.9064°N, 68.3441°W, 31.V-15.VI.2011, M. Roy & V. Webster // Old-growth white spruce & balsam fir forest, Lindgren funnel trap (1 ♂, LFC); Jacquet River Gorge P.N.A., 47.8257°N, 66.0764°W, 15–29.VI.2014, C. Alderson & V. Webster // Old *Populus balsamifera* stand near river, Lindgren funnel traps 1 m high under trees (1 ♂, RWC). **York Co.**, New Maryland, Charters Settlement, 45.8430°N, 66.7275°W, 8.X.2004, R.P. Webster, coll. // Regenerating mixed forest, baited with pile of decaying mushrooms (1 ♂, RWC).

######### Natural history.

Little is known about the biology of *Aleochara rubricalis*. One specimen was reported from a mouse nest (Klimaszewski 1984). In NB, specimens were collected in Lindgren funnel traps in an old black spruce (*Picea mariana* (Mill.) BSP) forest, an old-growth white spruce (*Picea glauca* (Moench) Voss) and balsam fir (*Abies balsamea* (L.) Mill.) forest, and an old balsam poplar (*Populus balsamifera* L.) stand near a river. One specimen was found among decaying mushrooms in a regenerating mixed forest. Adults were collected during May, June, and September.

######### Distribution in Canada and Alaska.

BC, ON, **NB** (Klimaszewski 1984; Bousquet et al. 2013). *Aleochara rubricalis* was previously known mainly from the west coast from BC south to CA and northern AZ, with one doubtful record from western ON (Klimaszewski 1984). The records presented above indicate that this species is transcontinental in Canada.

**Figures 1–8.**
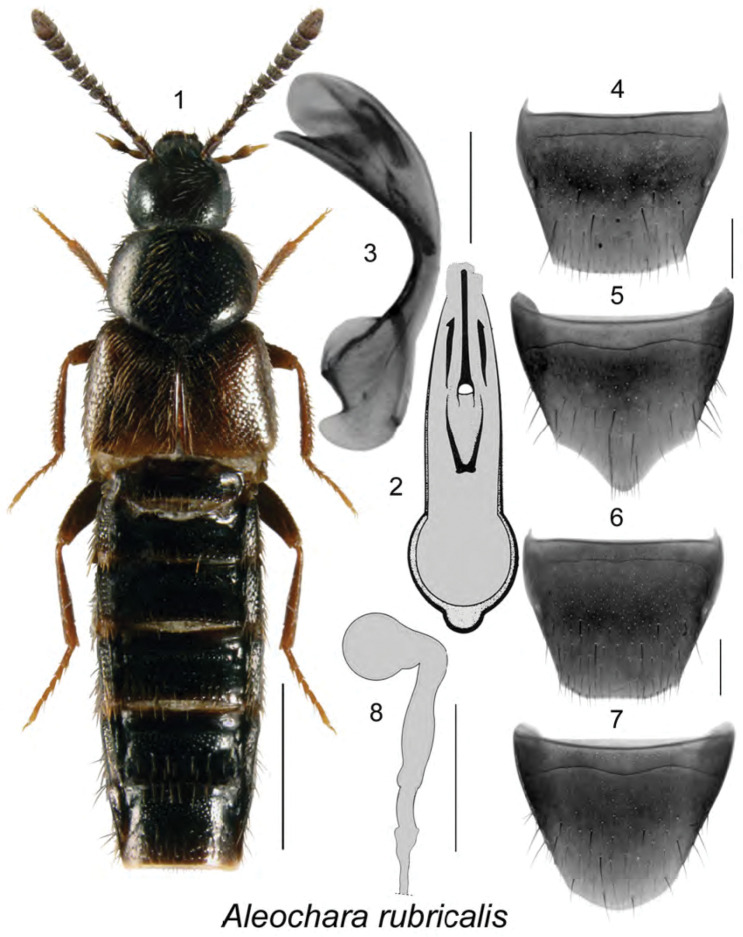
Aleochara (Calochara) rubricalis (Casey): **1**, habitus in dorsal view **2** median lobe of aedeagus in dorsal view **3** median lobe of aedeagus in lateral view **4** male tergite VIII **5** male sternite VIII **6** female tergite VIII **7** female sternite VIII **8** spermatheca **2**, **8** modified from Klimaszewski (1984). Scale bar of habitus = 1 mm; remaining scale bars = 0.2 mm.

######## Aleochara (Calochara) speculicollis

Taxon classificationAnimaliaColeopteraStaphylinidae

Bernhauer, 1901

Figs 9–16

Aleochara (Calochara) speculicollis (For diagnosis, see Klimaszewski 1984)

######### Material examined.

**Canada, New Brunswick, Restigouche Co.**, Jacquet River Gorge P.N.A., 47.8254°N, 66.0780°W, 13.VIII.2010, R.P. Webster // Mixed forest, in decaying chanterelle (1 ♀, RWC).

######### Natural history.

Almost nothing is known about the habitat and biology of this species. In NB, one specimen was found in a decaying chanterelle mushroom in a mixed forest. Elsewhere, one individual was sifted from deep layers of wet and moldy oak (*Quercus*) leaf litter (Klimaszewski 1984).

######### Distribution in Canada and Alaska.

AB, ON, QC, **NB** (Klimaszewski 1984; Bousquet et al. 2013). *Aleochara speculicollis* has a very spotty distribution, with most records from western North America. The previous easternmost record was from western QC (Klimaszewski 1984).

**Figures 9–16.**
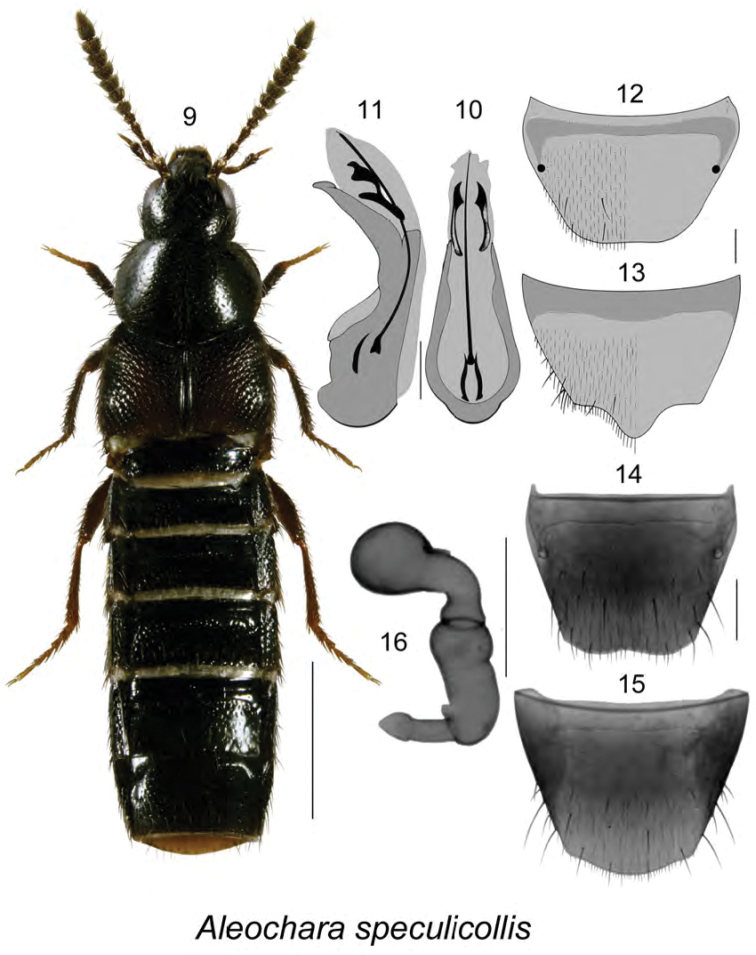
Aleochara (Calochara) speculicollis Bernhauer: **9** habitus in dorsal view **10** median lobe of aedeagus in dorsal view **11** median lobe of aedeagus in lateral view **12** male tergite VIII **13** male sternite VIII **14** female tergite VIII **15** female sternite VIII **16** spermatheca. **10–13** modified from Klimaszewski (1984). Scale bar of habitus = 1 mm; remaining scale bars = 0.2 mm.

######## Aleochara (Echochara) ocularis

Taxon classificationAnimaliaColeopteraStaphylinidae

Klimaszewski, 1984

Figs 17–24

Aleochara (Echochara) ocularis (For diagnosis, see Klimaszewski 1984)

######### Material examined.

**Canada, New Brunswick, Kent Co.**, Kouchibouguac N.P., 46.8072°N, 64.9082°W, 21.V.2015, R.P. Webster // Margin field/Jack pine forest, in litter in entrance to *Marmota monax* burrow (1 ♀, RWC). **York Co.**, Keswick Ridge, 45.9962°N, 66.8781°W, 25.V.2015, R.P. Webster // Margin field/hardwood forest, in litter in entrance to *Marmota monax* burrow (1 ♀, RWC).

######### Natural history.

This species has been found in entrances of fox (*Vulpes* sp.) and woodchuck (*Marmota monax* (L.)) burrows in early spring (April to June) (Klimaszewski 1984), and in caves (Klimaszewski and Peck 1986). Adults were taken from moist soil and grass roots near the burrow entrances, and in carrion in caves. The two specimens from NB were found in similar burrow habitats during May.

######### Distribution in Canada and Alaska.

MB, ON, QC, **NB** (Klimaszewski 1984; Bousquet et al. 2013).

**Figures 17–24.**
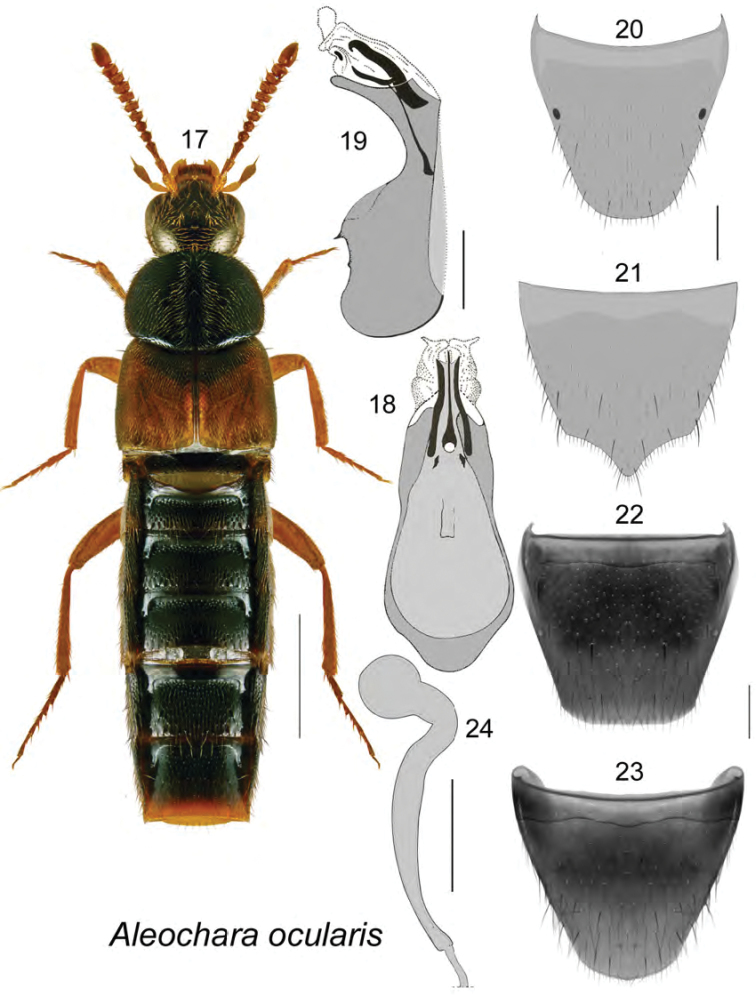
Aleochara (Echochara) ocularis Klimaszewski: **17** habitus in dorsal view **18** median lobe of aedeagus in dorsal view **19** median lobe of aedeagus in lateral view **20** male tergite VIII **21** male sternite VIII **22** female tergite VIII **23** female sternite VIII **24** spermatheca **19**, **18**, **24** modified from Klimaszewski (1984). Scale bar of habitus = 1 mm; remaining scale bars = 0.2 mm.

######## Tinotus caviceps

Taxon classificationAnimaliaColeopteraStaphylinidae

Casey, 1894

Figs 25–33

Tinotus caviceps (For diagnosis, see Klimaszewski et al. 2002)

######### Material examined.

**Canada, New Brunswick, York Co.**, New Maryland, Charters Settlement, 45.8430°N, 66.7275°W, 7.VI.2004, R.P. Webster, coll. // Regenerating mixed forest, pitfall trap (1 ♂, RWC); same locality and collector but 45.8428°N, 66.7279°W, 14.IX.2004 // Mixed forest, small sedge marsh, in moist grass litter (1 ♂, 1 sex undetermined, RWC).

######### Natural history.

In NB, specimens were collected from a pitfall trap and from moist grass litter in a small sedge marsh in a mixed forest. One specimen from QC was captured in a Luminoc pit-light trap (Klimaszewski et al. 2002); specimens from ON were collected in hedgerows beside soybean fields (Brunke 2011 [thesis]). Little else is known about the biology of this species. Adults were found during May, June, and September.

######### Distribution in Canada and Alaska.

ON, QC, **NB** (Klimaszewski et al. 2002; Majka and Klimaszewski 2008b; Bousquet et al. 2013).

######### Comments.

Brunke et al. (2012) provided additional evidence supporting the concept that *Tinotus caviceps* and *Tinotus trisecus* Casey are distinct species.

**Figures 25–33.**
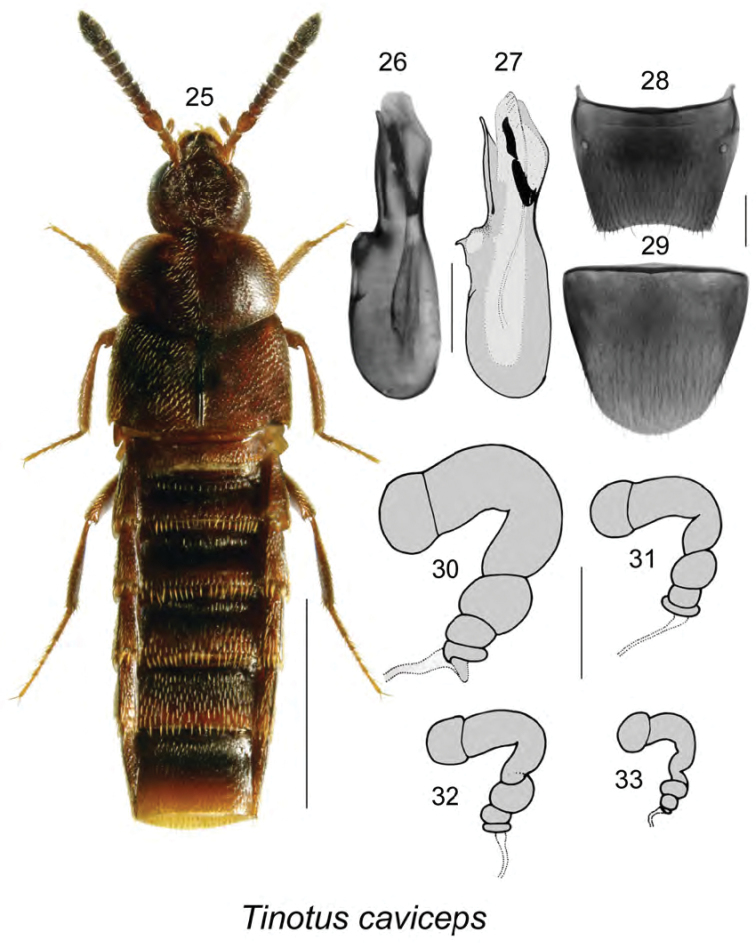
*Tinotus caviceps* Casey: **25** habitus in dorsal view **26**, **27** median lobe of aedeagus in lateral view **28** male tergite VIII **29** male sternite VIII **30–33** spermatheca **27**, **30–33** modified from Klimaszewski et al. (2002). Scale bar of habitus = 1 mm; remaining scale bars = 0.2 mm.

###### Tribe Athetini Casey, 1910

####### Subtribe Athetina Casey, 1910

######## Acrotona brachyoptera

Taxon classificationAnimaliaColeopteraStaphylinidae

Klimaszewski & Webster sp. n.

http://zoobank.org/658B1A2D-2996-4C2F-97BF-B3515B584577

Figs 34–41

######### Holotype (male).

**Canada, New Brunswick, Saint John Co.**, Chance Harbour off Rt. 790, 45.1355°N, 66.3672°W, 12.V.2008, R.P. Webster, coll. // Calcareous fen, in sphagnum and litter in depression with *Carex* (LFC). **Paratypes: Canada, New Brunswick, Carleton Co.**, Wakefield, “Bell Forest Nature Preserve”, 46.2210°N, 67.7210°W, 19.IV.2005, R.P. Webster, coll. // Rich Appalachian hardwood forest, in leaf litter on mound of soil (1 ♀, LFC). **Queens Co.**, ca. 3.5 km W of Lower Gagetown, 45.7497°N, 66.1846°W, 13.V.2008, R.P. Webster // Old red oak / red maple forest, in moist leaves on margin of vernal pool (1 ♂, RWC). **Sunbury Co.**, Acadia Research Forest, 29.VI.1999, 21.IX.1999, Site 2, Clearcut, Pitfall trap, G. Gesner, Coll. (2 ♂, LFC); same data but 22.VI.1999, Site 2, Select 2, (1 ♀, LFC); Acadia Research Forest, 45.9799°N, 66.3394°W, 18.VI.2007, R.P. Webster, coll. // Road 7 control, mature red spruce & red maple forest, sifting moss near brook (1 ♂, 1 ♀, RWC); same data but 14.V.2007, 18.VI.2007 // sifting leaf litter (2 sex undetermined, 1 ♂, 1 ♀, AFC); same data but 18.IX.2007 // sifting leaf litter & moss (1 sex undetermined, AFC); Acadia Research Forest, 45.9816°N, 66.3374°W, 18.IX.2007, R.P. Webster, coll. // Road 7 Regenerating Forest, 8.5 year old regenerating mixed forest, in sphagnum and leaf litter at bottom of old tire depression (2 sex undetermined, AFC); Acadia Research Forest, 46.0188°N, 66.3765°W, 14.V.2007, 14.V.2007, 17.VIII.2007, R.P. Webster, coll. // Road 16 control, mature red spruce & red maple forest, sifting moss (1 sex undetermined, 1 ♂, 1 ♀, AFC; 1 ♂, RWC); same data but 14.V.2007, 18.VII.2007 // sifting leaf litter (1 sex undetermined, 1 ♀, AFC); same data but 18.IX.2007 // sifting leaf litter & moss (1 sex undetermined, AFC); Acadia Research Forest, 46.0173°N, 66.3741°W, 14.V.2007, R.P. Webster, coll. // Road 16 Regenerating Forest, 8.5 year old regenerating mixed forest, sifting leaf litter & moss (1 sex undetermined, AFC); same data but 18.IX.2007 // in sphagnum and leaf litter at bottom of old tire depression (1 sex undetermined, AFC). **York Co.**, Canterbury, Browns Mtn. Fen, 45.8967°N, 67.6343°W, 2.V.2005, M. Giguère & R. Webster, coll. // Forested cedar fen, in litter at base of cedar (1 ♀, NBM); New Maryland, off Hwy 2, E of Baker Brook, 45.8760°N, 66.6252°W, 6.IV.2006, R.P. Webster, coll. // Old-growth cedar swamp, in moss & litter at base of cedar (1 sex undetermined, LFC); same data but 20.IV.2005 (1 ♂, LFC); New Maryland, Charters Settlement, 45.8331°N, 66.7410°W, 16.IV.2004, R.P. Webster, coll. // Mature red spruce & cedar forest, in moss & litter near small brook (1 ♀, CNC); New Maryland, Charters Settlement, 45.8428°N, 66.7279°W, 15.IV.2005, 20.IV.2005, R.P. Webster, coll. // Mixed forest, small sedge marsh in moist grass litter & sphagnum (1 ♂, CNC; 1 ♂, LFC); New Maryland, Charters Settlement, 45.8282°N, 66.7367°W, 9.IV.2005, R.P. Webster, coll. // *Carex* marsh, in moist sphagnum in *Carex* marsh (1 ♂, LFC); New Maryland, Charters Settlement, 45.8341°N, 66.7445°W, 22.IV.2005, R.P. Webster, coll. // Mature spruce & cedar forest, seepage area in saturated sphagnum & leaf litter (1 ♂, 1 ♀, NBM); New Maryland, Charters Settlement, 45.8428°N, 66.7235°W, 1.IV.2006, R.P. Webster, coll. (1 ♂, RWC); New Maryland, Charters Settlement, 45.8395°N, 66.7391°W, 29.III.2006, R.P. Webster, coll. // Mixed forest, under alders near small stream, in leaf litter (1 ♀, RWC); same data but 22.IV.2004 // Mixed forest, in leaf litter & moss near small shaded brook (1 ♂, LFC); New Maryland, Charters Settlement, 45.8342°N, 66.7450°W, 21.IV.2006, R.P. Webster, coll. // Mixed forest, margin of vernal pond in moist leaf litter (1 ♀, RWC); New Maryland, Charters Settlement, 45.8286°N, 66.7365°W, 11.VII.2006, R.P. Webster // Mature mixed forest, in gilled mushroom (1 ♂, RWC); Hwy 2 near Exit 271, 45.8986°N, 66.7918°W, 8.VI.2008, R.P. Webster, coll. // Mixed forest in leaf litter (sifting) (1 sex undetermined, 1 ♀, RWC); Kingsclear near Mazerolle Settlement, 45.8987°N, 66.7903°W, 9.IV.2006, R.P. Webster, coll. // Marsh with scattered alders, sifting grass & sphagnum at base of alders (1 ♀, LFC); 8.5 km W of Tracy, off Rt. 645, 45.6821°N, 66.7894°W, 6.V.2008, R.P. Webster // wet alder swamp, in leaf litter & grass on hummocks (1 ♀, RWC). **Ontario**, Alfred Bog, 17.VII.1982, L. LeSage, berl., litter, for., trail (1 ♂, 1 ♀, CNC).

######### Etymology.

This species is named for the short (brachyopterous, alternative spelling of brachypterous) elytra.

######### Description.

**Figures 34–41.**
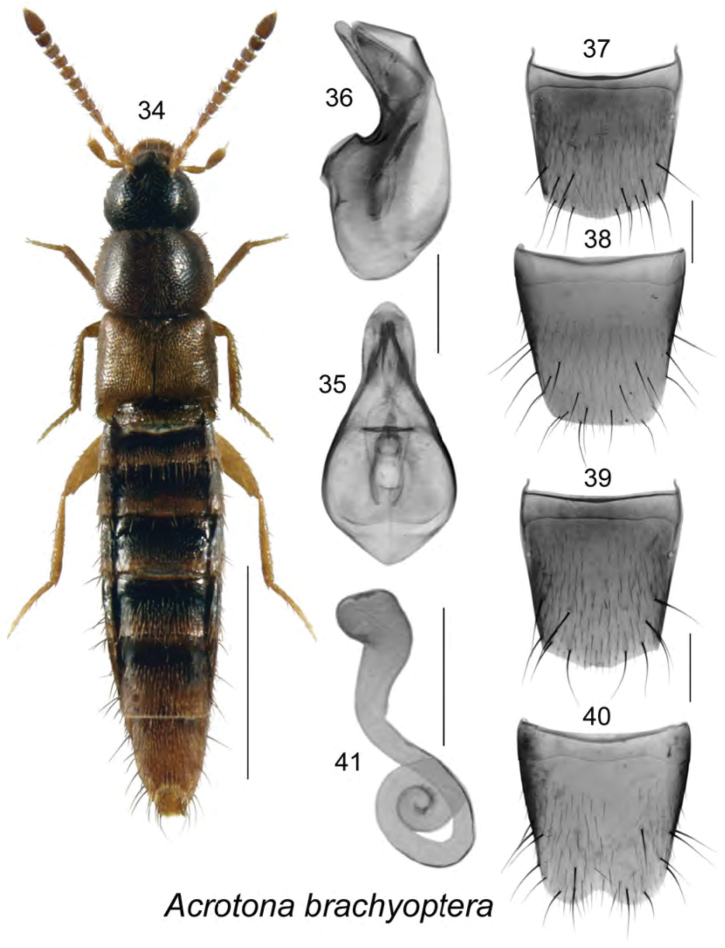
*Acrotona brachyoptera* Klimaszewski & Webster, sp. n.: **34** habitus in dorsal view **35** median lobe of aedeagus in dorsal view **36** median lobe of aedeagus in lateral view **37** male tergite VIII **38** male sternite VIII **39** female tergite VIII **40** female sternite VIII **41** spermatheca. Scale bar of habitus = 1 mm; remaining scale bars = 0.2 mm.

Body length 2.8–3.0 mm, very narrow, uniformly dark brown except for paler elytra, apex of abdomen, legs and basal antennal articles (Fig. 34); integument strongly glossy, moderately densely punctate and pubescent, pubescence short and adhering to body; head about one-sixth narrower than pronotum, rounded posteriorly with small eyes about three times shorter than postocular area; antennae with articles V–X transverse; pronotum broad, transverse, distinctly broader than elytra, posterolateral margin completely rounded; elytra shorter than pronotum; abdomen tapering apically. **Male.** Median lobe of aedeagus with bulbus broad, oval, tubus narrowly triangular in dorsal view (Fig. 35), arcuate ventrally in lateral view (Fig. 36); internal sac structures as illustrated (Figs 35, 36); tergite VIII slightly pointed apically (Fig. 37); sternite VIII elongate, truncate apically with base sinuate (Fig. 38). **Female.** Tergite VIII more apically produced than that of male (Fig. 39); sternite VIII deeply emarginate apically (Fig. 40); spermatheca with club-shaped capsule and coiled stem (Fig. 41).

######### Distribution.

Known from ON and NB, Canada.

######### Natural history.

In NB, this species was found in an old red maple (*Acer rubrum* L.) forest, mixed forests, a wet alder (*Alnus* sp.) swamp, a mature red spruce (*Picea* *rubens* Sarg.) and red maple forest, a rich Appalachian hardwood forest, in a *Carex* marsh, small sedge marsh, marsh with scattered alders, in old-growth eastern white cedar (*Thuja occidentalis* L.) swamps and forests, and in 8.5-year-old regenerating mixed forests. Adults occurred in moss and litter near brooks, in moss and litter at the base of cedar, in moss and litter in red spruce and cedar forests, in leaf litter under alders near a stream, in leaf litter and grass on hummocks in a wet alder swamp, in grass litter and sphagnum in marshes, moist leaves on the margin of a vernal pool, in sphagnum and leaf litter at the bottom of old tire depressions, and one specimen was collected from a gilled mushroom. Adults were collected during March, April, May, June, July, August, and September.

######### Comments.

This species has genitalic structures similar to those of *Acrotona subpygmaea* but differs by its narrower body, the pronotum broader than the elytra with posterolateral margin completely rounded near base, elytra shorter than pronotum, and its body is darker with paler, reddish-brown elytra and apical portion of the abdomen. In *Acrotona subpygmaea*, the posterolateral margin of pronotum is slightly angulate near the base and the body is uniformly dark brown.

######## Acrotona sphagnorum

Taxon classificationAnimaliaColeopteraStaphylinidae

Klimaszewski & Webster sp. n.

http://zoobank.org/20AB1664-C794-4041-8CBE-9F704D543D55

Figs 42–49

######### Holotype (male).

**Canada, New Brunswick**, New Maryland, Charters Settlement, 45.8285°N, 66.7365°W, 21.V.2006, R.P. Webster, coll. // Mature eastern white cedar & red spruce forest, in moss & litter (1 ♂, LFC). **Paratypes: Canada, New Brunswick, Charlotte Co.**, Hwy 3 at Deadwater Brook, 45.4745°N, 67.1225°W, 23.IV.2006, R.P. Webster, coll. // Black spruce forest, in sphagnum (1 ♀, LFC; 2 ♂, 3 ♀, RWC); same data but 3.VI.2005 // Black spruce forest, in moist sphagnum (1 ♂, RWC); S of Little Pocologan River, 45.15365°N, 66.62687°W, 7.V.2007, R.P. Webster coll. // Black spruce and tamarack bog, in litter and moss on “moose” trail (1 sex undetermined, LFC). **Restigouche Co.**, Jacquet River Gorge PNA, 47.8189°N, 65.9952°W, 25.VI.2008, R.P. Webster, coll. // Eastern white cedar swamp with black spruce, in moist sphagnum (1 ♀, RWC); NE of jct. Little Tobique R. & Red Br., 47.4501°N, 67.0577°W, 24.V.2007, R.P. Webster, coll. // Old-growth eastern white cedar swamp, in moist sphagnum (1 ♂, 1 ♀, RWC). **York Co.**, Manner’s Sutton, Upper Brockway, 45.5684°N, 67.0993°W, 23.IV.2006, R.P. Webster, coll. // Forested black spruce bog, in sphagnum (1 ♀, RWC).

######### Etymology.

*Sphagnorum* is a Latin adjective derived from the generic name of *Sphagnum* sp., a dominant plant in most of the habitats where this species was found.

######### Description.

**Figures 42–49.**
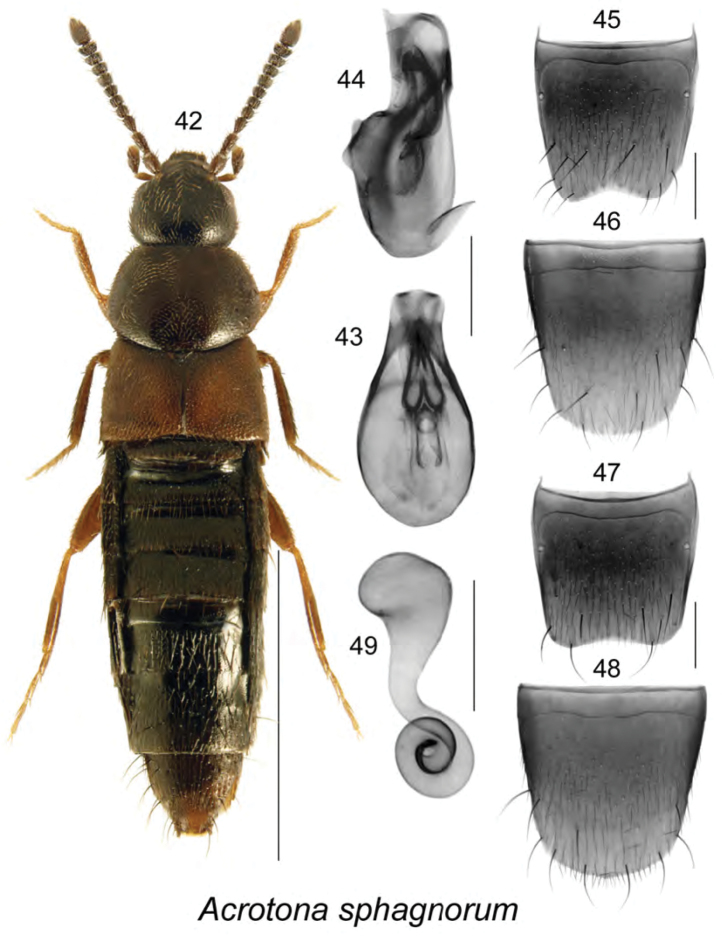
*Acrotona sphagnorum* Klimaszewski & Webster, sp. n.: **42** habitus in dorsal view **43** median lobe of aedeagus in dorsal view **44** median lobe of aedeagus in lateral view **45** male tergite VIII **46** male sternite VIII **47** female tergite VIII **48** female sternite VIII **49** spermatheca. Scale bar of habitus = 1 mm; remaining scale bars = 0.2 mm.

Body length 2.3 mm, moderately narrow, uniformly dark brown except for reddish legs and two small yellowish-red areas on each elytron near suture (Fig. 42); integument strongly glossy, densely punctate and pubescent, pubescence short and adhering to body; head round, about one-fourth narrower than pronotum, rounded posteriorly with eyes shorter than postocular area; antennae with articles V–X transverse; pronotum shield-shaped, transverse, much broader than elytra at base; elytra shorter than pronotum; abdomen tapering apically. **Male.** Median lobe of aedeagus with bulbus broad, oval, tubus narrowly triangular in dorsal view (Fig. 43), straight ventrally in lateral view (Fig. 44); internal sac structures as illustrated (Figs 43, 44); tergite VIII emarginate apically (Fig. 45); sternite VIII elongate, rounded apically, slightly sinuate at base (Fig. 46). **Female.** Tergite VIII slightly emarginate (Fig. 47); sternite VIII rounded apically (Fig. 48); spermatheca with club-shaped capsule and coiled stem (Fig. 49).

######### Distribution.

Known only from NB, Canada.

######### Natural history.

This species was found in moist sphagnum in forested black spruce bogs, and in eastern white cedar swamps and forests. One individual was found in moss and litter in a moose (*Alces alces*) trail through a black spruce and tamarack (*Larix laricina* (Du Roi) Koch) bog. Adults were collected during April, May, and June.

######### Comments.

This species is distinct externally because of its shield-shaped pronotum, which is slightly wider than the elytra, which contributes to a habitus that is somewhat similar to species of *Mocyta*. It may be distinguished from all other Nearctic *Acrotona*, by the unique shape of its genital structures, including male and female tergite VIII.

######## Acrotona subpygmaea

Taxon classificationAnimaliaColeopteraStaphylinidae

(Bernhauer, 1909)

Figs 50–58

Acrotona subpygmaea (For details, see Brunke et al. 2012)

######### Diagnosis.

**Figures 50–58.**
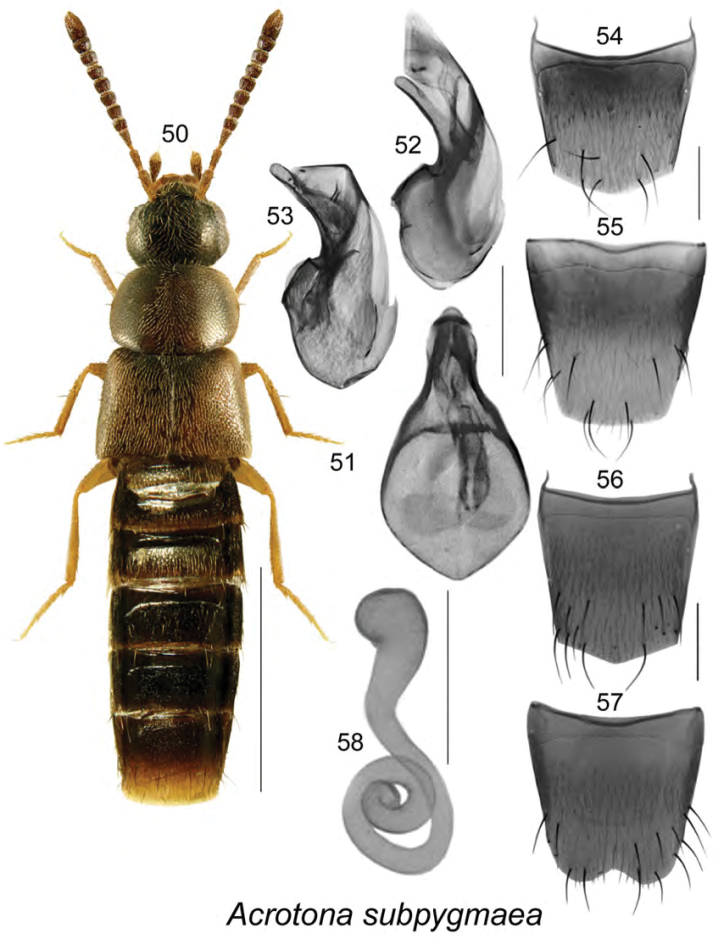
*Acrotona subpygmaea* (Bernhauer): **50**, habitus in dorsal view **51** median lobe of aedeagus in dorsal view **52, 53** median lobe of aedeagus in lateral view **54** male tergite VIII **55** male sternite VIII **56** female tergite VIII **57** female sternite VIII **58** spermatheca. Scale bar of habitus = 1 mm; remaining scale bars = 0.2 mm.

Body length 2.6–2.8 mm, moderately narrow, uniformly dark brown except for paler legs and basal antennal articles (Fig. 50); integument moderately glossy, densely punctate and pubescent, pubescence short and adhering to body; head about one-third narrower than pronotum, rounded posteriorly with eyes shorter than postocular area; antennae with articles V–X transverse; pronotum broad, transverse, as wide as elytra at base and posterolateral margin slightly angulate, not completely rounded; elytra as long as pronotum or slightly longer; abdomen subparallel for most of its length. **Male.** Median lobe of aedeagus with bulbus broad, oval, tubus narrowly triangular in dorsal view (Fig. 51), arcuate ventrally in lateral view (Figs 52, 53); internal sac structures as illustrated (Figs 51–53); tergite VIII slightly pointed apically (Fig. 54); sternite VIII elongate, truncate apically and bearing sinuate base (Fig. 55). **Female.** Tergite VIII more apically produced than that of male (Fig. 56); sternite VIII deeply emarginate apically (Fig. 57); spermatheca with club-shaped capsule and coiled stem (Fig. 58). This species has genitalic structures almost identical to those of *Acrotona brachyoptera*, but differs by its broader body, longer elytra, and darker and more uniform body color.

######### Material examined.

**New Brunswick, Northumberland Co.**,12 km SSE of Upper Napan, 46.8991°N, 65.3682°W, 7.VI.2006, R.P. Webster, coll. // Eastern white cedar swamp, in moist leaf litter (2 ♂, LFC); ca. 2.5 km W of Sevogle, 47.0876°N, 65.8613°W, 28.V.2013, R.P. Webster // Old jack pine forest, vernal pond margin, in leaf litter (1 ♂, AFC, 1 ♂, RWC). **Queens Co.**, Canning, Grand Lake near Scotchtown, 45.8762°N, 66.1817°W, 25.V.2006, R.P. Webster // Silver maple swamp near lake margin, margin of vernal pond in moist leaves (2 ♂, 1 ♀, RWC); ca. 3.5 km W of Lower Gagetown, 45.7497°N, 66.1846°W, 13.V.2008, R.P. Webster // old red oak/red maple forest, in moist leaves on margin of vernal pond (1 ♂, RWC); near Queenstown, 45.6904°N, 66.1455°W, 13.V.2008, R.P. Webster // old-growth hardwood forest, in leaf litter near seepage and brook (1 ♂, RWC). **Restigouche Co.**, Jacquet River Gorge P.N.A., 47.7491°N, 66.1114°W, 24.VI.2008, R.P. Webster // Hardwood forest, among moist leaves in dried snow-melt pool (1 ♀, RWC). **Sunbury Co.**, Maugerville, Portobello Creek N.W.A., 45.9031°N, 66.4268°W, 11.IX.2006, R.P. Webster, oak & red maple forest, on gilled mushrooms (1 ♂, LFC; 1 ♂, RWC); Acadia Research Forest, 46.0188°N, 66.3765°W, 18.VI.2007, R.P. Webster, coll. // Road 16 control, mature red spruce & red maple forest, sifting leaf litter & moss (2 ♂, AFC); same data but 14.V.2007 // sifting leaf litter (1, sex undetermined, AFC); Acadia Research Forest, 46.0173°N, 66.3741°W, 17.VIII.2007, 18.IX.2007, R.P. Webster, coll. // Road 16 Regenerating Forest, 8.5 year old regenerating mixed forest // in sphagnum and leaf litter at bottom of old tire depression (1 sex undetermined, 1 ♂, AFC). **York Co.**, trail to Browns Mtn. Fen, 45.9033°N, 67.6260°W, 2.V.2005, M. Giguère & R. Webster, coll. // Mixed forest with cedar, margin of vernal pond in moist leaf litter (1 ♂, CNC); Fredericton, Nashwaaksis River at Rt. 105, 45.9850°N, 66.6900°W, 6.V.2006, R.P. Webster // River margin, in flood debris on upper river margin (1 ♀, RWC); Charters Settlement, 45.8341°N, 66.7445°W, 22.IV.2005, 27.IV.2005, R.P. Webster, coll. // Mature mixed forest, margin of vernal pond among moist leaves (3 ♀, LFC); Charters Settlement, 45.8340°N, 66.7450°W, 29.V.2008, R.P. Webster // Mature mixed forest, margin of vernal pond among moist leaves (1 ♀, NBM, 1 ♀, RWC); same data but 1.IV.2007 // Mixed forest, under bark of stump sticking out of snow (1 ♀, LFC); Charters Settlement, 45.8286°N, 66.7365°W, Old-growth red spruce & cedar forest, in moss & litter at base of tree (1 ♂, LFC); 9.2 km W of Tracy, off Rt. 645, 45.6837°N, 66.8809°W, 22.V.2008, R.P. Webster, coll. // *Carex* marsh adjacent to slow (flowing) stream, in *Carex* hummock (1 ♀, LFC).

######### Natural history.

Most specimens of *Acrotona subpygmaea* from NB were found among moist leaves along margins of vernal ponds and snow-melt pools in various forest types. These included an old jack pine (*Pinus banksiana* Lamb.) forest, silver maple (*Acer saccharinum* L.) swamp, an old red oak (*Quercus rubra* L.)/red maple forest, hardwood forests, an eastern white cedar swamp, a mature red spruce and red maple forest, and a mature mixed forest. A few were found in leaf litter near a seepage and brook, in sphagnum and leaf litter at bottom of an old tire depression in a regenerating mixed forest, in leaf litter and moss, in flood debris on an upper river margin, in a *Carex* hummock in a *Carex* marsh, and in a gilled mushroom. One individual was found under bark of a stump sticking out of snow in early April. Most adults were collected in May, with a few in April, June, August, and September. Brunke et al. (2012) reported this species from similar habitats in ON and Majka and Klimaszewski (2010) reported it in bark of dead white pine in NS.

######### Distribution in Canada and Alaska.

ON, **NB**, NS (Majka and Klimaszewski 2010; Brunke et al. 2012; Bousquet et al. 2013); although previously reported from NB (Klimaszewski et al. 2005), this was a misidentification by V. Gusarov and is described here as *Acrotona brachyoptera*.

######## Alevonota gracilenta

Taxon classificationAnimaliaColeopteraStaphylinidae

(Erichson, 1839)†

Figs 59–67

Alevonota gracilenta (For diagnosis, see Klimaszewski et al. 2013a)

######### Material examined.

**New Brunswick, Carleton Co.**, Jackson Falls, “Bell Forest”, 46.2200°N, 67.7231°W, 21.VI-3.VII.2012, C. Alderson & V. Webster // Rich Appalachian hardwood forest with some conifers, Lindgren funnel trap in canopy of *Fraxinus americana* (1 ♀, RWC). **Restigouche Co.**, Dionne Brook P.N.A., 47.9030°N, 68.3503°W, 27.VI-14.VII.2011, M. Roy & V. Webster // Old-growth northern hardwood forest, Lindgren funnel trap (1 ♀, RWC). **York Co.**, Douglas, Currie Mountain, 45.9844°N, 66.7592°W, 24.VI-9.VII.2013, C. Alderson & V. Webster // Mixed forest with *Quercus rubra*, Lindgren funnel trap 1 m high under *Quercus rubra* (1 ♀, RWC); Douglas, Currie Mountain, 45.9832°N, 66.7564°W, 3–15.V.2013, C. Alderson & V. Webster // Old *Pinus strobus* stand, Lindgren funnel trap 1 m high under *Pinus strobus* (1 ♂, RWC).

######### Natural history.

Specimens of this adventive species in NB were captured in Lindgren funnel traps in hardwood forests, a mixed forest, and an old white pine (*Pinus strobus* L.) stand. In southern ON, specimens were captured in pitfall traps in and near agricultural fields (Brunke et al. 2012). In the western Palaearctic, most specimens were collected in passive traps in unforested habitats, but the true habitat remains unknown (Assing and Wunderle 2008).

######### Distribution in Canada and Alaska.

ON, **NB** (Brunke et al. 2012; Bousquet et al. 2013).

######### Comments.

*Alevonota gracilenta* was first reported from North America by Brunke et al. (2012) based on specimens collected in southern ON. They suggested that the introduction may have been recent.

**Figures 59–67.**
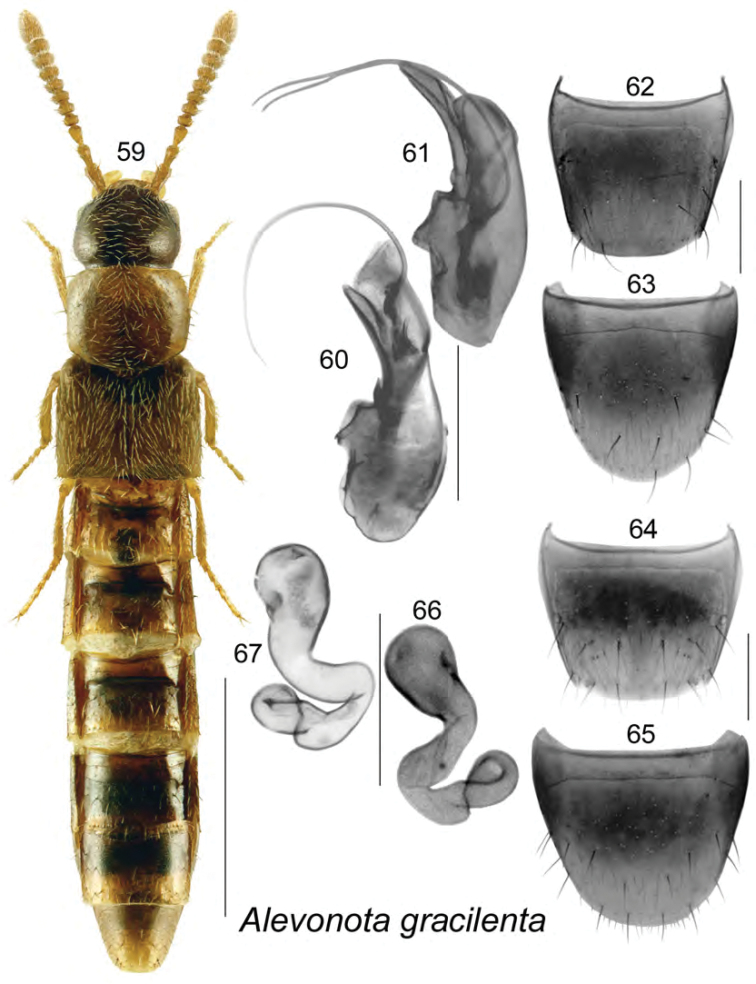
*Alevenota gracilenta* (Erichson): **59** habitus in dorsal view **60, 61** median lobe of aedeagus in lateral view **62** male tergite VIII **63** male sternite VIII **64** female tergite VIII **65** female sternite VIII **66, 67** spermatheca **60**, **67** after Assing and Wunderle (2008) **59, 61–63** after Brunke et al. (2012). Scale bar of habitus = 1 mm; remaining scale bars = 0.2 mm.

######## Atheta (Datomicra) whitehorsensis

Taxon classificationAnimaliaColeopteraStaphylinidae

Klimaszewski & Godin, 2012

Figs 68–75

Atheta (Datomicra) whitehorsensis (For diagnosis, see Klimaszewski et al. 2012)

######### Material examined.

**New Brunswick, York Co.**, Charters Settlement, 45.8395°N, 66.7391°W, 30.IX.2007, R.P. Webster, coll. // Mixed forest, in decaying (moldy) corncobs & cornhusks (1 ♂, RWC).

######### Natural history.

The only known specimen of *Atheta whitehorsensis* from NB was collected from a pile of decaying corncobs. In the YT, specimens were sifted from soil in a black spruce stand (Klimaszewski et al. 2012).

######### Distribution in Canada and Alaska.

YT, **NB** (Klimaszewski et al. 2012). The specimen from NB represents the first record of this species from eastern Canada, suggesting that *Atheta whitehorsensis* is transcontinental.

**Figures 68–75.**
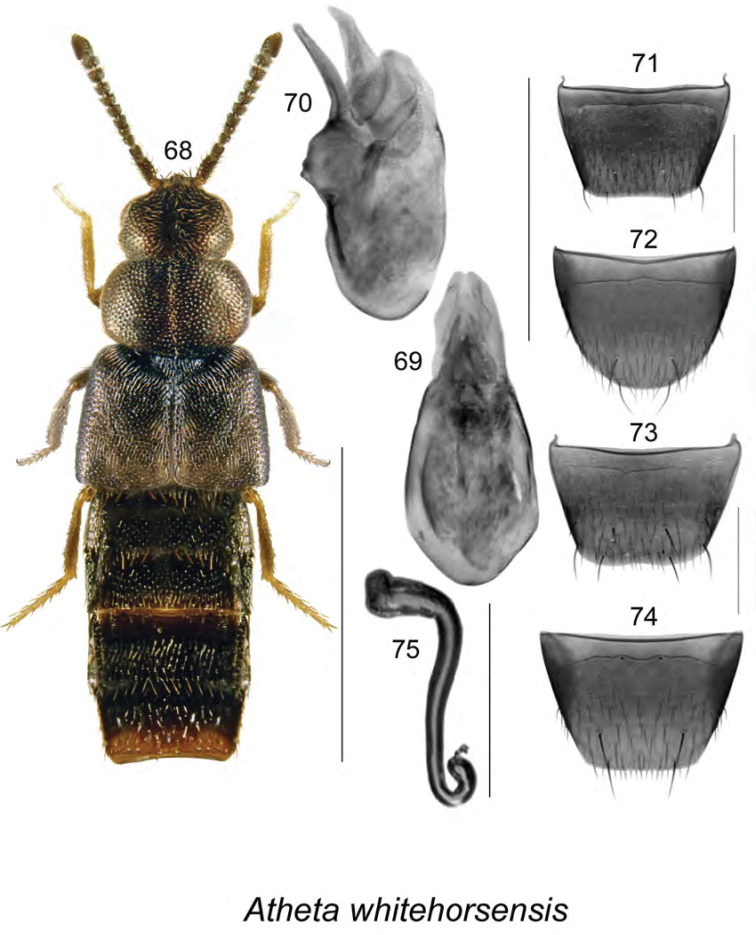
*Atheta whitehorsensis* Klimaszewski & Godin: **68**, habitus in dorsal view **69** median lobe of aedeagus in dorsal view **70** median lobe of aedeagus in lateral view **71** male tergite VIII **72** male sternite VIII **73** female tergite VIII **74** female sternite VIII **75** spermatheca **68**, **70–75** after Klimaszewski et al (2012). Scale bar of habitus = 1 mm; remaining scale bars = 0.2 mm.

######## Atheta (Dimetrota) alphacrenuliventris

Taxon classificationAnimaliaColeopteraStaphylinidae

Klimaszewski & Webster sp. n.

http://zoobank.org/30B80D83-4B95-4093-988D-4C10DC078D42

Figs 76–83

######### Holotype (male).

**Canada, New Brunswick, Northumberland Co.**, ca. 2.5 km W of Sevogle, 47.0876 N, 65.8613°W, 28.V.2013, R.P. Webster // old jack pine forest, vernal pond margin in coyote dung (LFC). **Paratype: Canada, New Brunswick, Restigouche Co.**, Mount Atkinson, 447 m elev., 47.8192 N, 68.2618°W, 23.VI.2010, R.P. Webster, coll. // boreal forest, small shaded spring-fed brook with mossy margin, sifting moss (1 ♀, RWC).

######### Etymology.

A prefix *alpha*- added to the specific name *crenuliventris*, a species very similar to the new species.

######### Description.

**Figures 76–83.**
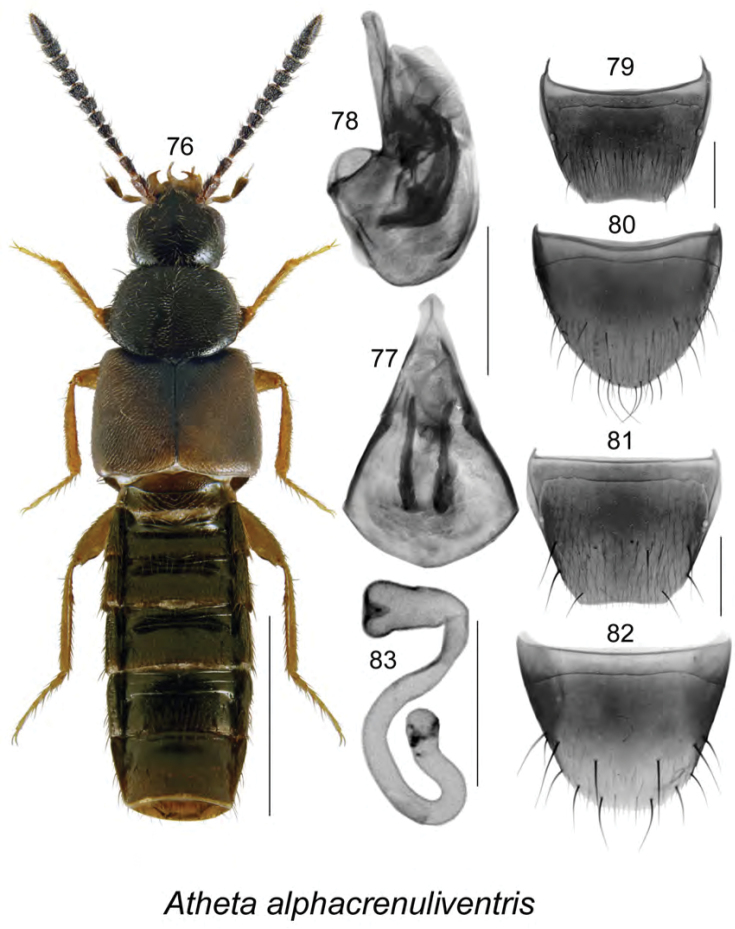
Atheta (Dimetrota) alphacrenuliventris Klimaszewski & Webster, sp. n.: **76** habitus in dorsal view **77** median lobe of aedeagus in dorsal view **78** median lobe of aedeagus in lateral view **79** male tergite VIII **80** male sternite VIII **81** female tergite VIII **82** female sternite VIII **83** spermatheca. Scale bar of habitus = 1 mm; remaining scale bars = 0.2 mm.

Body length 3.2–3.6 mm, moderately narrow, elongate; head, pronotum, and abdomen dark brown to nearly black, elytra yellowish brown with triangular apical dark section near suture, legs yellowish brown or reddish brown, and antennae dark brown (Fig. 76); integument moderately glossy; forebody with meshed microsculpture and minute and dense punctation and pubescence; head rounded and slightly angular posterolaterally, with large eyes, longer than postocular area in dorsal view; antennae with articles V–X subquadrate to slightly transverse; pronotum rounded, slightly transverse, wider than head and distinctly narrower than elytra, pubescence directed laterad from midline of disk; elytra transverse, with pubescence directed posterolaterad and forming waves posteromedially; abdomen subparallel, narrower than elytra. **Male.** Median lobe of aedeagus with bulbus broad, tubus triangular in dorsal view (Fig. 77), and broad, straight ventrally, with apical part broadly elongate in lateral view (Fig. 78); internal sac with complex structures (Figs 77, 78); tergite VIII shallowly emarginate apically and sinuate, lateral proximity with small tooth on each side (Fig. 79); sternite VIII broadly parabolic (Fig. 80). **Female.** Tergite VIII truncate apically (Fig. 81); sternite VIII broadly rounded apically (Fig. 82); spermatheca club shaped, with narrow sac-shaped capsule bearing narrow apical invagination, stem sinuate half-looped posteriorly (Fig. 83).

######### Distribution.

Known only from NB, Canada.

######### Natural history.

One specimen was found in coyote dung on the margin of a vernal pond in a jack pine forest and another from moss along a small shaded spring-fed brook in a boreal (spruce–fir) forest. Adults were collected during May and June.

######### Comments.

*Atheta alphacrenuliventris* is very similar externally and genitalically to *Atheta crenuliventris* Bernhauer and *Atheta pseudocrenuliventris* Klimaszewski. It may be distinguished from those two species by the absence of a crenulated apical margin on male tergite VIII (Fig. 79) and its differently shaped spermatheca (Fig. 83), and from *Atheta crenuliventris*,﻿ it differs by having the tubus of the median lobe of the aedeagus broader in lateral view (Fig. 78). Externally, its elytra are more reddish brown than those of *Atheta crenuliventris*, which are dark brown, and *Atheta pseudocrenuliventris*, which are light brown. The most reliable characters for distinguishing it from the other two species are genital characters (shape of the apical margin of male tergite VIII, shape of the median lobe of the aedeagus in lateral view, and shape of the spermatheca). (See Klimaszewski et al. 2011; Figs 112, 285a–c, 412 for characters for comparison of *Atheta pseudocrenuliventris* with *Atheta alphacrenuliventris*).

######## Atheta (Dimetrota) bubo

Taxon classificationAnimaliaColeopteraStaphylinidae

Klimaszewski & Webster sp. n.

http://zoobank.org/52F0D9B5-F397-46CB-8525-83978718B639

Figs 84–87

######### Holotype (male).

**Canada, New Brunswick, Westmorland Co.**, Sackville, near Ogden Mill, 45.92155°N, 64.38925°W, 12.V,2006, Scott Makepeace, coll. // black spruce forest, in nest contents of Great Horned Owl – *Bubo virginiensis* (LFC).

######### Etymology.

The species name *bubo* is the generic name of *Bubo virginensis*, the great horned owl, from the nest contents of which the holotype specimen was found, used in apposition.

######### Description.

**Figures 84–87.**
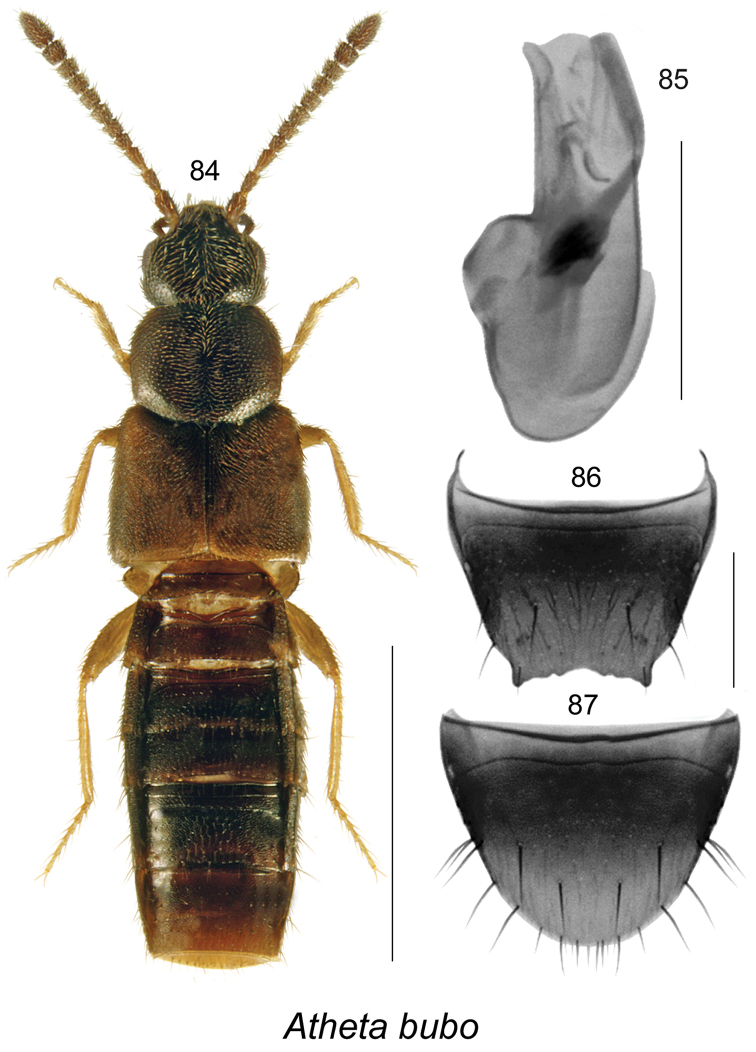
Atheta (Dimetrota) bubo Klimaszewski & Webster, sp. n.: **84** habitus in dorsal view **85** median lobe of aedeagus in lateral view **86** male tergite VIII **87** male sternite VIII. Scale bar of habitus = 1 mm; remaining scale bars = 0.2 mm.

Body length 2.8 mm, subparallel, moderately flattened, dark brown with darker head, pronotum, and central part of abdomen, elytra with darker scutellar region, legs yellowish brown (Fig. 84); integument moderately glossy and more so on abdomen, densely punctate and pubescent, except for head and abdomen; meshed microsculpture of forebody dense and strong with hexagonal sculpticells; head narrower than pronotum, angular posteriorly, eyes large and as long as postocular area dorsally; antennae with articles V–X subquadrate to slightly transverse; pronotum broadest in about middle of its length, rounded laterally and basally, slightly transverse, narrower than elytra, posterior shoulders angular; elytra wider and slightly longer than pronotum; abdomen subparallel. **Male.** Apical margin of tergite VIII with broadly V-shaped apical emargination with small crenulations and two large lateral teeth (Fig. 86); median lobe of aedeagus with bulbus moderately large, tubus moderately long, straight with apex slightly produced ventrally in lateral view, apex narrowly triangular and rounded (Fig. 85), internal sac structures pronounced at base of tubus (Fig. 85). **Female.** Unknown.

######### Natural history.

This species is known only from a single male found in the nest contents of a great horned owl (*Bubo virginensis*) in a black spruce forest in May.

######### Distribution.

Known only from NB, Canada.

######### Comments.

The body shape of this species is somewhat similar to species of *Atheta picipennis* species group, but the genitalia are unique in its form and are not close to any species of *Dimetrota*.

######## Atheta (Dimetrota) campbelli

Taxon classificationAnimaliaColeopteraStaphylinidae

(Lohse, 1990)

Figs 88–95

Atheta (Dimetrota) campbelli (For diagnosis, see Lohse et al. 1990, Klimaszewski et al. 2011)

######### Material examined.

**New Brunswick, Carleton Co.**, Jackson Falls, 46.2216°N, 67.7231°W, 8.V.2013, 31.V.2013, R.P. Webster // Meadow/hayfield, in dung in entrance to burrow of *Marmota monax* (5 ♂, 6 ♀, RWC; 1 ♀, NBM). **Kent Co.**, Kouchibouguac N.P., S. Kouchibouguac Campground, 46.8279°N, 64.9397°W, 21.V.2015, R.P. Webster // Margin field/Jack pine forest, in litter in entrance to *Marmota monax* burrow (1 ♂, NBM). **York Co.**, Keswick Ridge, 45.9962°N, 66.8781°W, 25.V.2015, R.P. Webster // Margin field/hardwood forest, in litter in entrance to *Marmota monax* burrow (1 ♂, RWC).

######### Natural history.

Specimens from NB were found in dung and litter at the entrance of a woodchuck burrow in a meadow, jack pine forest adjacent to a field, and a hardwood forest adjacent to a meadow. In NF, adults were captured in unbaited and carrion-baited pitfall traps in balsam fir forests and in rotting mushrooms in a mixed forest (Klimaszewski et al. 2011). In ON, it was collected in a hedgerow (Brunke et al. 2012). Lohse et al. (1990) reported the species from bear and caribou dung. Adults were collected during May in NB and June to August elsewhere (Klimaszewski et al. 2011, Lohse et al. 1990).

######### Distribution in Canada and Alaska.

AK, YT, ON, **NB**, LB, NF (Lohse et al. 1990; Klimaszewski et al. 2011; Brunke et al. 2012; Bousquet et al. 2013).

**Figures 88–95.**
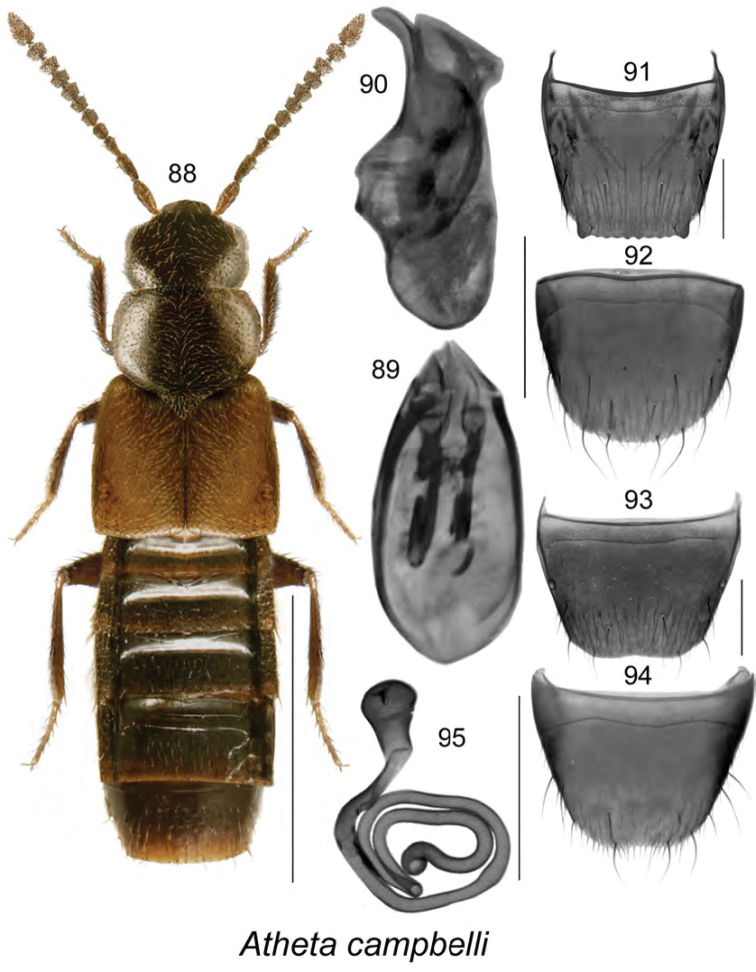
Atheta (Dimetrota) campbelli (Lohse): **88** habitus in dorsal view **89** median lobe of aedeagus in dorsal view **90** median lobe of aedeagus in lateral view **91** male tergite VIII **92** male sternite VIII **93** female tergite VIII **94** female sternite VIII **95** spermatheca. Scale bar of habitus = 1 mm; remaining scale bars = 0.2 mm.

######## Atheta (Dimetrota) chartersensis

Taxon classificationAnimaliaColeopteraStaphylinidae

Klimaszewski & Webster sp. n.

http://zoobank.org/E9A3C17C-2812-4E60-9A04-4CBE335E8F39

Figs 96–103

######### Holotype (male).

**Canada, New Brunswick, York Co.**, Charters Settlement, 45.8395°N, 66.7391°W, 26.V.2008, R.P. Webster, coll. // Mixed forest, in decaying moldy corncobs and cornhusks (LFC). **Paratypes: Canada, New Brunswick, Northumberland Co.**, ca. 2.5 km W of Sevogle, 47.0876°N, 65.8613°W, 28.V.2013, R.P. Webster // Old jack pine forest, in coyote dung (1 ♂, AFC; 1 ♂, RWC). **Saint John Co.**, Chance Harbour off Rt. 790, 45.1391°N, 66.3696°W, 16.IX.2008, R.P. Webster, coll. // Mixed forest, in decaying gilled mushroom (1 ♀, RWC). **York Co.**, New Maryland, Charters Settlement, 45.8395°N, 66.7391°W, 22.VIII.2006, 27.IV.2006, 5.IX.2006, 14.VI.2008, 20.VI.2008, 27.VIII.2008, R.P. Webster, coll. // Mixed forest, in pile of decaying (moldy) corncobs & cornhusks (4 ♂, 5 ♀, RWC); same data except 5.IX.2006 (1 ♀, NBM); same data except 5.VIII.2006, 22.VIII.2006, 6.IX.2006 (1 ♂, 2 ♀, CNC); same data except 27.IX.2005, 20.VIII.2006, 5.IX.2006 (1 ♂, 3 ♀, LFC).

######### Etymology.

This species is named after Charters Settlement, the locality where the holotype and most of the paratypes were collected.

######### Description.

**Figures 96–103.**
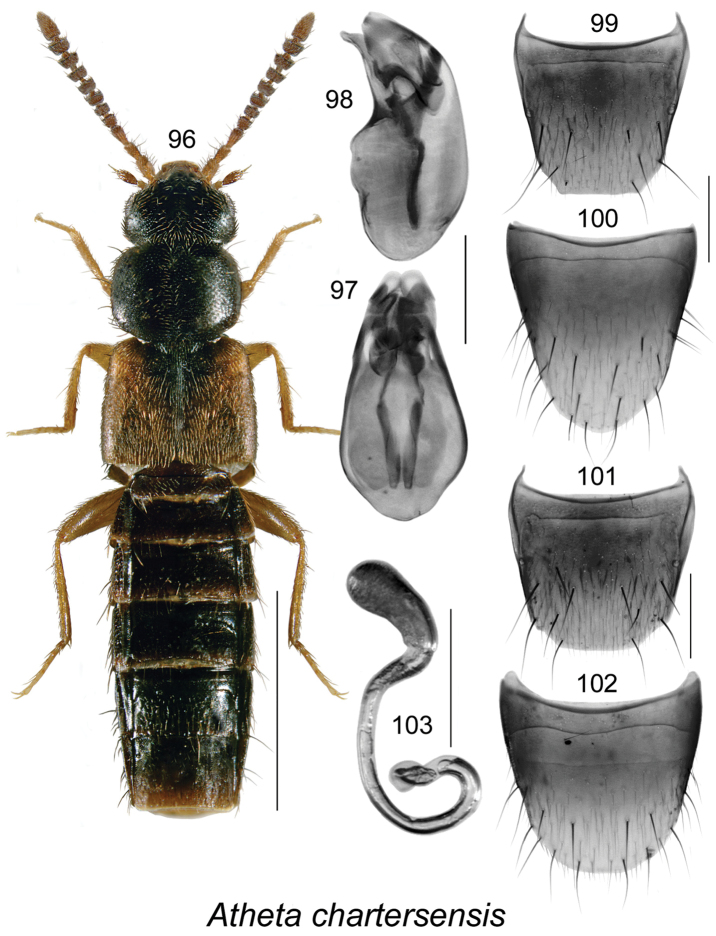
Atheta (Dimetrota) chartersensis Klimaszewski & Webster, sp. n.: **96** habitus in dorsal view **97** median lobe of aedeagus in dorsal view **98** median lobe of aedeagus in lateral view **99** male tergite VIII **100** male sternite VIII **101** female tergite VIII **102** female sternite VIII **103** spermatheca. Scale bar of habitus = 1 mm; remaining scale bars = 0.2 mm.

Body length 3.4–3.5 mm, narrowly elongate, subparallel; head, pronotum, and most of abdomen except for apical part black, elytra, legs, and antennae brown or light brown (Fig. 96); forebody with minute and sparse punctation, moderately glossy; head slightly narrower than pronotum, angular posteriorly, with small eyes, antennae with articles V–X strongly transverse and progressively more so toward apex; pronotum transverse, as broad as elytra and only slightly wider than head, pubescence directed outward laterally from midline of disk; elytra with pubescence directed posteriad; abdomen at middle as broad as elytra, broadly arcuate laterally. **Male.** Median lobe of aedeagus with bulbus broad, oval, tubus short, triangular in dorsal view (Fig. 97), and straight and strongly produced ventrally at apex in lateral view (Fig. 98); internal sac with two elongate sclerites in bulbus and complex structures in tubus (Figs 97, 98); tergite VIII bluntly truncate apically with angular lateral edges (Fig. 99); sternite VIII rounded apically and slightly pointed (Fig. 100). **Female.** Tergite VIII truncate apically (Fig. 101); sternite VIII broadly rounded apically (Fig. 102); spermatheca with elongate club-shaped capsule and arcuate stem looped and twisted posteriorly (Fig. 103).

######### Distribution.

Known only from NB, Canada.

######### Natural history.

Most adults were collected from a pile of decaying moldy corncobs and cornhusks near a composter adjacent to a mixed forest in a residential area. Two individuals were collected from coyote dung in an old jack pine forest; another from a decaying mushroom. Specimens were collected during April, May, June, August, and September.

######### Comments.

This species belongs to the *Modesta* group of Atheta (Dimetrota), with three currently known species: Atheta (Dimetrota) modesta (Melsheimer), Atheta (Dimetrota) pseudomodesta Klimaszewski, and the present new species. All three species share similar body characteristics, similar shape of the spermatheca, ventrally strongly produced apex of the median lobe of the aedeagus, and truncate apical margin of male tergite VIII with angular lateral edges forming more or less distinct teeth. *Atheta chartersensis* differs from *Atheta modesta* and *Atheta pseudomodesta* by narrower body (Fig. 96); elytra dark reddish brown mottled with black, which is slightly contrasting with the color of head and pronotum (elytra is light reddish yellow in the other two species and strongly contrasting with color of head and pronotum); by elytra equal in length to pronotum (elytra is longer than pronotum in the other two species), antennae are dark and II-III basal articles slightly paler and articles VII-X strongly transverse (slightly transverse or subquadrate in the other two species and articles I-III light yellowish red strongly contrasting with remaining dark brown articles), median lobe has narrower apex and internal sac structures are differently shaped (Figs 97, 98) than those in *Atheta modesta* and *Atheta pseudomodesta*. For illustrations of *Atheta modesta* and *Atheta pseudomodesta*, ﻿see Gusarov (2003a) and Klimaszewski et al. (2007), respectively.

######## Atheta (Dimetrota) cranberriensis

Taxon classificationAnimaliaColeopteraStaphylinidae

Klimaszewski & Webster sp. n.

http://zoobank.org/CC3E9825-C321-4B35-90F6-240D79B35D5B

Figs 104–111

######### Holotype (male).

**Canada, New Brunswick, Queens Co.**, Cranberry Lake P.N.A., 46.1125°N, 65.6075W, 21–27.V.2009, R. Webster & M.-A. Giguère, coll. // red oak forest, Lindgren funnel trap (LFC). **Paratypes: Canada, New Brunswick, Kent Co.**, Kouchibouguac N.P., near Callanders Beach, 46.8072°N, 64.9082°W, 21.V.2015, R.P. Webster // Margin field/Jack pine forest, in litter in entrance to *Marmota monax* burrow (1 ♂, 3 ♀, RWC). **Queens Co.**, Cranberry Lake P.N.A., 46.1125°N, 65.6075°W, 12–21.V.2009, R. Webster & M.-A. Giguère // Red oak forest, Lindgren funnel traps (1 ♂, 1 ♀, RWC); same data except 21–27.V.2009 (1 ♀, RWC); Jemseg, 45.8412°N, 66.1195°W, 14–28.V.2012, C. Alderson & V. Webster // Hardwood woodland near seasonally flooded marsh, Lindgren funnel trap 1 m high under *Quercus macrocarpa* (♂, RWC). **York Co.**, New Maryland, Charters Settlement, 45.8395°N, 66.7391°W, 17.V.2010, 8.V.2011, R.P. Webster // Mixed forest opening, collected with net during evening flight between 16:30 and 19:00 h (1 ♀, RWC); same data except 21.IV.2010 (1 ♀, LFC).

######### Etymology.

This species is named after Cranberry Lake P.N.A. (Protected Natural Area) where the type specimen and most paratypes were collected.

######### Description.

**Figures 104–111.**
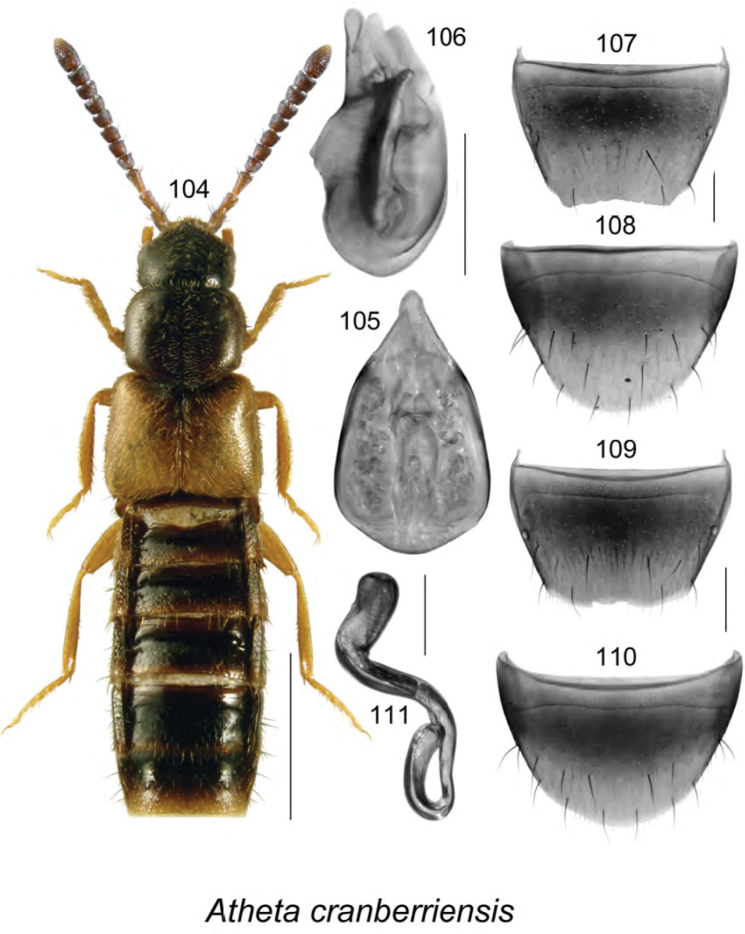
Atheta (Dimetrota) cranberriensis Klimaszewski & Webster, sp. n.: **104** habitus in dorsal view **105** median lobe of aedeagus in dorsal view **106** median lobe of aedeagus in lateral view **107** male tergite VIII **108** male sternite VIII **109** female tergite VIII **110** female sternite VIII **111** spermatheca. Scale bar of habitus = 1 mm; remaining scale bars = 0.2 mm.

Body length 3.2–3.8 mm, moderately narrow, subparallel (Fig. 104); antennae, head, pronotum, and most of abdomen except for apical part dark brown, elytra and legs light brown to yellowish brown; forebody with minute and sparse punctation, moderately glossy; head angular posteriorly, with moderately large eyes; antennae with articles V–X slightly transverse and progressively more so toward apex; pronotum angular posterolaterally and rounded anteriorly, transverse, wider than head and narrower than elytra, pubescence directed laterad from midline of disk; elytra with pubescence directed posterolaterad from midline of disk; abdomen at middle as broad as elytra, broadly arcuate laterally. **Male.** Median lobe of aedeagus with bulbus broad, oval, tubus short, triangular in dorsal view (Fig. 105), and straight with rounded apex in lateral view (Fig. 106); internal sac with structures not apparent (Figs 105, 106); tergite VIII truncate apically, slightly emarginate medially bearing some crenulation, with angular lateral edges (Fig. 107); sternite VIII rounded apically (Fig. 108). **Female.** Tergite VIII with apical margin arcuate (Fig. 109); sternite VIII broadly rounded apically (Fig. 110); spermatheca with elongate sac-shaped capsule and sinuate stem narrowly looped posteriorly (Fig. 111).

######### Distribution.

Known only from NB, Canada.

######### Natural history.

Most adults were captured in Lindgren funnel traps in a red oak forest and a hardwood woodland near a seasonally flooded marsh. Other individuals were collected with a net between 16:30 and 19:00 h in a mixed forest opening. Four individuals were collected from litter from the entrance of a groundhog burrow. It is possible that this species is associated with ground-nesting mammals, but more sampling from this habitat is required. All specimens were captured in May.

######### Comments.

This species is externally very similar to *Atheta alesi* Klimaszewski & Brunke, and has similar body coloration and pubescence pattern with the pubescence appearing soft, but has a much broader and more elongate body (body length 3.2–3.8 mm compared with 2.4–2.6 mm in *Atheta alesi* (Klimaszewski et al. 2012); has more robust antennae with articles VIII-X less transverse that those in *Atheta alesi*, has broader (almost as broad as base of elytra) and differently shaped pronotum with strongly angular posterior angles, and elytra less contrasting yellow. The genitalia are superficially similar in the two species, but the apical margin of male tergite VIII in *Atheta cranberriensis* has a more arcuate emargination (Fig. 107), and that of *Atheta alesi* has a more angular broadly V-shaped emargination.

######## Atheta (Dimetrota) giguereae

Taxon classificationAnimaliaColeopteraStaphylinidae

Klimaszewski & Webster sp. n.

http://zoobank.org/562BA62B-ACD7-4447-B334-EBE4BEBBC443

Figs 112–119

######### Holotype (male).

**Canada, New Brunswick, Charlotte Co.**, near New River, 45.21217°N, 66.61595°W, 7.V.2007, R.P. Webster, coll. // Mature eastern white cedar swamp/forest in moss and leaf litter near stream (LFC). **Paratypes: Canada, New Brunswick, Carleton Co.**, Meduxnekeag Valley Nature Preserve, 46.1935°N, 67.6825°W, 19.IV.2005, R.P. Webster, coll. // Mixed forest, in moist moss (1 sex undetermined, LFC; 1 sex undetermined, RWC). **Charlotte Co.**, S of Little Pocologan River, 45.1546°N, 66.6254°W, 7.V.2007, R.P. Webster, coll. // Mature eastern white cedar swamp/forest, in moss & leaf litter (1 ♂, RWC); near New River, 45.21217°N, 66.61595°W, 7.V.2007, R.P. Webster, coll. // Mature eastern white cedar swamp/forest, in moss & leaf litter near stream (1 ♂, RWC); Kent Island, WS, sweeping, 23.VII.2008, Meredith Steck (1 ♀, NSPM); Kent Island, Wet SF, beating, 15.VII.2008, white spruce, Meredith Steck (1 ♂, NSPM); Kent Island, 23.VII.2008, Meredith Steck (1 ♂, 1 ♀, NSPM); **Restigouche Co.**, Little Tobique River near Red Brook, 47.4462°N, 67.0689°W, 24.V.2007, R.P. Webster, coll. // Old-growth eastern white cedar swamp, in moss & leaf litter near brook (1 ♀, RWC); Dionne Brook P.N.A., 47.9030°N, 68.3503°W, 15–27.VI.2011, M. Roy & V. Webster // Old-growth northern hardwood forest, Lindgren funnel trap (1 ♂, CNC; 1 ♂, RWC). **York Co.**, Charters Settlement, 45.8331°N, 66.7410°W, 16.IV.2004, R.P. Webster, coll. // Mature red spruce & cedar forest, in moss & litter near small brook (1 sex undetermined, RWC); Charters Settlement, 45.8341°N, 66.7445°W, 22.IV.2005, R.P. Webster, coll. // Mature red spruce & cedar forest, in seepage area in saturated sphagnum & leaf litter (1 sex undetermined, LFC); New Maryland, off Hwy 2, E of Baker Brook, 45.8760°N, 66.6252°W, 6.IV.2005, R.P. Webster, coll. // Old-growth cedar swamp, in moss & leaf litter at base of cedar (1 ♂, 1 sex undetermined, RWC); Kingsclear, Mazorolle Settlement, 45.8717°N, 66.8273°W, 28.IV.2006, R.P. Webster, coll. // Eastern white cedar swamp, in moss & leaf litter near brook (1 ♂, 1 ♀, RWC; 1 ♀, LFC); Douglas, Currie Mountain, 45.9832°N, 66.7564°W, 3–15.V.2013, C. Alderson & V. Webster // Old *Pinus strobus* stand, Lindgren funnel trap 1 m high under *Pinus strobus* (1, AFC); Canterbury, near Browns Mtn. Fen, 45.89508°N, 67.63326°W, 1.VI.2005, M. Giguère & R. Webster, coll. (1 ♀, LFC). **Nova Scotia**, **Guysborough Co.**, Malay Lake, com. thin. mat. red spruce for., FIT, 2–15.VI.1997, DeLancey J. Bishop (1 ♂, NSPM); Malay Lake, red spruce (thin), 2–15.VI.1997, D.J. Bishop, 278, 688 (2 ♀, NSPM). **Halifax Co.**, Pockwock Lake, 2–25.VI.1997, DeLancy J. Bishop, pre-com. thin. red spruce for., FIT (1 ♀, NSPM); Halifax, Sandy Lake, Red Spruce (>120), 15–30.VI.1997, D.J. Bishop 827 (1 sex undetermined, NSPM); Halifax, Abraham’s Lake, red spruce (old), 14.V-2.VI.1997, D.J. Bishop 92 (2 ♀, NSPM); Margaret Bay, Big. St., red spruce (m), 29.VII-13.VII.1997, D.J. Bishop (1 ♂, NSPM); Soldier Lake, 13.VI.2005, SB Trap, J. Gordon (1 ♀, NSPM). **Hants Co.**, Leminster, mat. Red spr./hemlock forest FIT, 2–15.VI.1997, DeLancey J. Bishop (1 ♀, NSPM); Panuke Lake, red spruce (45), 15–30.VI.1997, D.J. Bishop, 795 (1 ♀, NSPM); Little Armstrong Lake, red spruce (75), 14.V-2.VI.1997, D.J. Bishop 222 (1 ♀, NSPM); Armstrong Lake, 75 yr fire origin red spruce FIT, 14.V-2.VI.1997, DeLancey J. Bishop (1 ♂, NSPM). **Lunenburg Co.**, Card Lake, red spruce/hemlock, 29.VII-13.VIII.1997, D. J. Bishop 1802 (1 ♀, NSPM). **Ontario, Northumberland Co.**, Peters Woods Nat. Res., 44°7'27"N, 78°2'21"W, forest, Lindgren funnel, 12–26, VII.2011, Brunke and Paiero, debu01147325 (1 ♀, LFC).

######### Etymology.

This species is dedicated to Marie-Andrée Giguère, wife of Reginald Webster, who has accompanied and assisted him on many collecting trips over the years and whose support made many of the new discoveries in New Brunswick possible.

######### Description.

**Figures 112–119.**
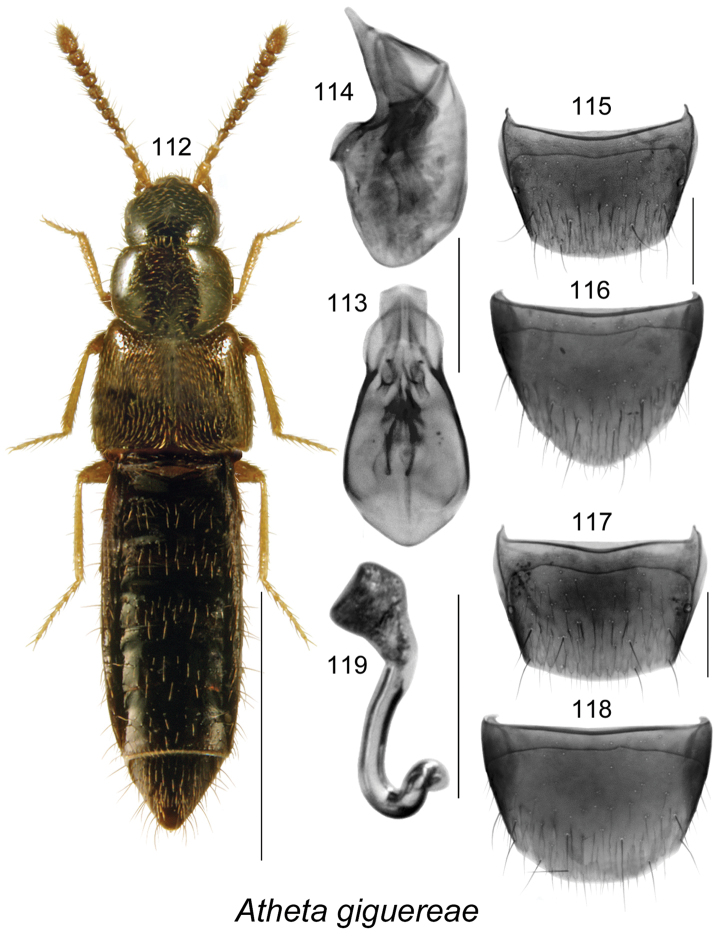
Atheta (Dimetrota) giguereae Klimaszewski & Webster, sp. n.: **112** habitus in dorsal view **113** median lobe of aedeagus in dorsal view **114** median lobe of aedeagus in lateral view **115** male tergite VIII **116** male sternite VIII **117** female tergite VIII **118** female sternite VIII **119** spermatheca. Scale bar of habitus = 1 mm; remaining scale bars = 0.2 mm.

Body length 2.7 mm, narrowly elongate; head, pronotum, elytra, and abdomen dark brown, legs and antennae light brown (Fig. 112); integument strongly glossy; forebody with minute and sparse punctation, and sparse pubescence; head rounded posteriorly, with moderately large eyes; antennae with articles V–X slightly transverse and progressively more transverse toward apex; pronotum rounded anteriorly and posterolaterally, transverse, wider than head and narrower than elytra, pubescence directed laterad from midline of disk; elytra slightly transverse, with pubescence directed posterolaterad and some with wavy pattern near posterior suture; abdomen subparallel, narrower than elytra. **Male.** Median lobe of aedeagus with bulbus narrowly oval, tubus broad, short, and rounded in dorsal view (Fig. 113), and produced ventrally and with apical part triangular in lateral view (Fig. 114); internal sac with complex structures (Figs 113, 114); tergite VIII truncate apically and broadly arcuate (Fig. 115); sternite VIII almost evenly rounded apically (Fig. 116). **Female.** Tergite VIII with apical margin arcuate (Fig. 117); sternite VIII broadly rounded apically (Fig. 118); spermatheca with broad pitcher-shaped capsule with large apical invagination and sinuate stem narrowly looped and twisted posteriorly (Fig. 119).

######### Distribution.

Known from ON, NB, and NS, Canada.

######### Natural history.

In NB, *Atheta giguereae* was found in mature and old-growth eastern white cedar swamps, a mixed forest, an old-growth northern hardwood forest, and an old white pine stand. Adults were sifted from moss and leaf litter near streams and brooks and from moist moss in these forests. A few individuals were captured in Lindgren funnel traps. Specimens from NS were captured in flight intercept traps in red spruce and red spruce–hemlock forests. Adults were collected from April to mid-August.

######### Comments.

We tentatively affiliated this species with the subgenus *Dimetrota*. The median lobe of the aedeagus of *Atheta giguereae* resembles that of *Atheta terranovae* Klimaszewski & Langor, in general morphology but the spermatheca is of a different type than any of the described Nearctic species.

######## Atheta (Dimetrota) makepeacei

Taxon classificationAnimaliaColeopteraStaphylinidae

Klimaszewski & Webster sp. n.

http://zoobank.org/2E754A90-9AB1-405C-81F9-3E6A8837EFB5

Figs 120–127

######### Holotype (male).

**Canada, New Brunswick, Carleton Co.**, Hay Settlement, 46.0339°N, 67.5797°W, 24.V.2007, S. Makepeace & R. Webster, coll. // nest box contents of Barred Owl (1 litre), moist smelly (urine smell) organic material (mostly wood chips), with small bones and insect parts (LFC). **Paratypes: Canada, New Brunswick, Carleton Co.**, Hay Settlement, 46.0339°N, 67.5797°W, 24.V.2007, S. Makepeace & R. Webster, coll. // nest box contents of Barred Owl (1 litre), moist smelly (urine smell) organic material (mostly wood chips), with small bones and insect parts (1 ♀, LFC); Benton, 45.99611°N, 67.58640°W, 24.V.2007, S. Makepeace & R. Webster, coll. // Nest contents of Barred Owl, young chicks present, moist smelly organic material and regurgitated pellets, feathers, fur, & small bones (1 ♀, RWC); Jackson Falls, “Bell Forest”, 46.2200°N, 67.7230°W, 7.VIII.2009, R.P. Webster // Rich Appalachian Hardwood Forest, on gilled mushroom (1 ♀, RWC). **Queens Co.**, Rees, near Grand Lake, 46.00164°N, 65.94656°W, 29.V.2007, S. Makepeace & R. Webster, coll. // Nest contents of Barred Owl, moist smelly organic material and regurgitated pellets, feathers, fur, & small bones (1 ♂, RWC).

######### Etymology.

This species is named in honor of Scott Makepeace who collected the contents from barred owl (*Strix varia* Barton) nests that contained most specimens of this species.

######### Description.

**Figures 120–127.**
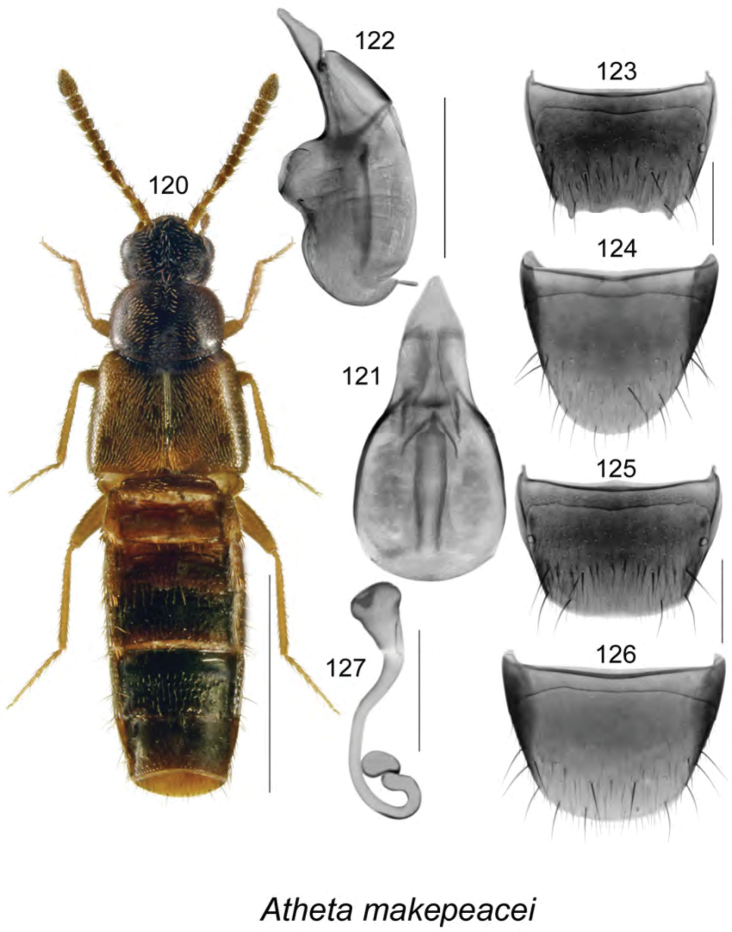
Atheta (Dimetrota) makepeacei Klimaszewski & Webster, sp. n.: **120** habitus in dorsal view **121** median lobe of aedeagus in dorsal view **122** median lobe of aedeagus in lateral view **123** male tergite VIII **124** male sternite VIII **125** female tergite VIII **126** female sternite VIII **127** spermatheca. Scale bar of habitus = 1 mm; remaining scale bars = 0.2 mm.

Body length 2.7 mm, moderately narrowly elongate; head, pronotum, and most of abdomen except for basal part black, antennae, legs brown, and elytra brown mottled with black (Fig. 120); integument moderately glossy; forebody with minute and moderately dense punctation and pubescence; head rounded posteriorly, with moderately large eyes, antenna with articles V–X slightly transverse and progressively more so toward apex; pronotum rounded anteriorly and posterolaterally, transverse, wider than head and narrower than elytra, pubescence directed laterad from midline of disk; elytra transverse, with pubescence directed posterolaterad and some with wavy pattern near suture; abdomen at base almost as broad as elytra, broadly arcuate laterally. **Male.** Median lobe of aedeagus with bulbus broad, oval, tubus long, narrowly triangular in dorsal view (Fig. 121), and produced ventrally, with apical part enlarged and narrowly triangular in lateral view (Fig. 122); internal sac with elongate structures (Figs 121, 122); tergite VIII with two apico-lateral teeth and sinuate apical margin (Fig. 123); sternite VIII rounded apically (Fig. 124). **Female.** Tergite VIII with apical margin broadly arcuate (Fig. 125); sternite VIII broadly rounded apically (Fig. 126); spermatheca with broad and elongate club-shaped capsule bearing large apical invagination and with sinuate stem narrowly looped and twisted posteriorly (Fig. 127).

######### Distribution.

Known only from NB, Canada.

######### Natural history.

Four of the adults were collected from the nest contents of barred owls (which nest in tree holes) that consisted of moist smelly organic material with regurgitated pellets, feathers, fur, and small bones. Another specimen was found in a gilled mushroom. It is possible that this species is associated with birds and other species that nest in tree holes. This species was found in old hardwood forests during May and August.

######### Comments.

The aedeagus of *Atheta makepeacei* is unique for the triangular apical part of the tubus and the narrow apex in lateral view (Fig. 122); the shape of the spermatheca is also different from the remaining Nearctic *Atheta* species known to us. We have tentatively affiliated this species with the subgenus *Dimetrota* based on external body characteristics.

######## Atheta (Dimetrota) mcalpinei

Taxon classificationAnimaliaColeopteraStaphylinidae

Klimaszewski & Webster sp. n.

http://zoobank.org/CCB32217-89A7-47AD-BB6A-191934EADADC

Figs 128–132

######### Holotype (male).

**Canada, New Brunswick, Northumberland Co.**, ca. 2.5 km W of Sevogle, 47.0876°N, 65.8613°W, 27.VIII.2013, R.P. Webster // Old jack pine forest, in rotting gilled mushroom (LFC). **Paratype. New Brunswick, Northumberland Co.**, ca. 2.5 km W of Sevogle, 47.0876°N, 65.8613°W, 27.VIII.2013, R.P. Webster // Old jack pine forest, in rotting gilled mushroom (1 ♂, RWC).

######### Etymology.

Named for Dr. Donald McAlpine, Curator and Head, Zoology Section of the New Brunswick Museum in recognition of his work studying and promoting research on the invertebrate and vertebrate fauna of NB.

######### Description.

**Figures 128–132.**
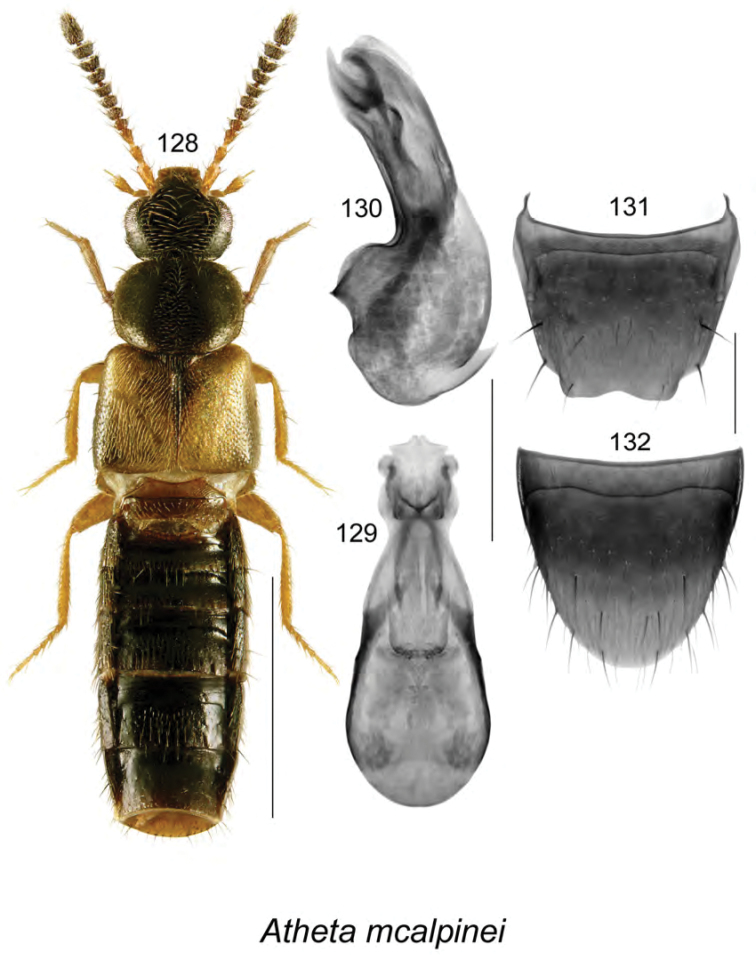
Atheta (Dimetrota) mcalpinei Klimaszewski & Webster, sp. n.: **128** habitus in dorsal view **129** median lobe of aedeagus in dorsal view **130** median lobe of aedeagus in lateral view **131** male tergite VIII **132** male sternite VIII. Scale bar of habitus = 1 mm; remaining scale bars = 0.2 mm.

Body length 2.9–3.0 mm, broadest at elytra; head, pronotum, and abdomen dark brown to nearly black, elytra yellowish brown mottled with dark brown, legs, bases of antennae, and maxillary palps yellowish brown (Fig. 128); integument moderately glossy with meshed microsculpture; forebody with fine and sparse punctation and pubescence except denser on elytra; head rounded and arcuate posterolaterally, with large eyes, each about as long as postocular area; antennae with articles V–X subquadrate to strongly transverse; pronotum transverse, rounded on sides, slightly wider than head and distinctly narrower than elytra, pubescence directed laterad from midline of disk; elytra transverse, with pubescence directed posterolaterad and forming waves posteriorly; abdomen gradually narrowed posteriad, narrower than elytra and arcuate laterally. **Male.** Median lobe of aedeagus with bulbus broad, narrowly oval, streamlined, tubus broad, triangular in dorsal view (Fig. 129), and slightly produced ventrally at apex, with apical part narrowly elongate in lateral view, venter approximately straight (Fig. 130); internal sac with distinct complex structures (Figs 129, 130); tergite VIII emarginate apically with sinuate margin and with two angular lateral processes (Fig. 131); sternite VIII elongate and rounded apically (Fig. 132). **Female.** Unknown.

######### Distribution.

Known only from NB, Canada.

######### Natural history.

Specimens were collected from rotting gilled mushrooms in a jack pine forest.

######### Comments.

This species bears some general resemblance to *Atheta remulsa* Casey from which it differs by less transverse antennal articles VII-X, darker elytra (Fig. 128), differently shaped median lobe of aedeagus with tubus straight and apex not strongly oriented ventrally (Fig. 130). For illustrations of *Atheta remulsa*,﻿ see Klimaszewski et al. (2011).

######## Atheta (Dimetrota) petitcapensis

Taxon classificationAnimaliaColeopteraStaphylinidae

Klimaszewski & Webster sp. n.

http://zoobank.org/45AFE2E2-EE52-441B-B395-E5BA0F6044D2

Figs 133–140

######### Holotype (male).

**Canada, New Brunswick, Westmorland Co**., Petit Cap, 46.1879°N, 64.1503°W, 17.VI.2014, M.-A. Giguère & R.P. Webster, coll. // sandy sea beach, under sea wrack and grass debris (LFC). **Paratypes**: Same data as the holotype (1 ♀, LFC; 3 ♀, RWC); same data except: 19.VI.2012, R.P. Webster & D. Sabine, coll. // sandy barrier sea beach, sifting drift material, mostly dried/decaying sea wrack (1 ♀, LFC; 1 ♂, 1 ♀, RWC)

######### Etymology.

Named after the village of Petit Cap where the holotype and paratypes were collected.

######### Description.

**Figures 133–140.**
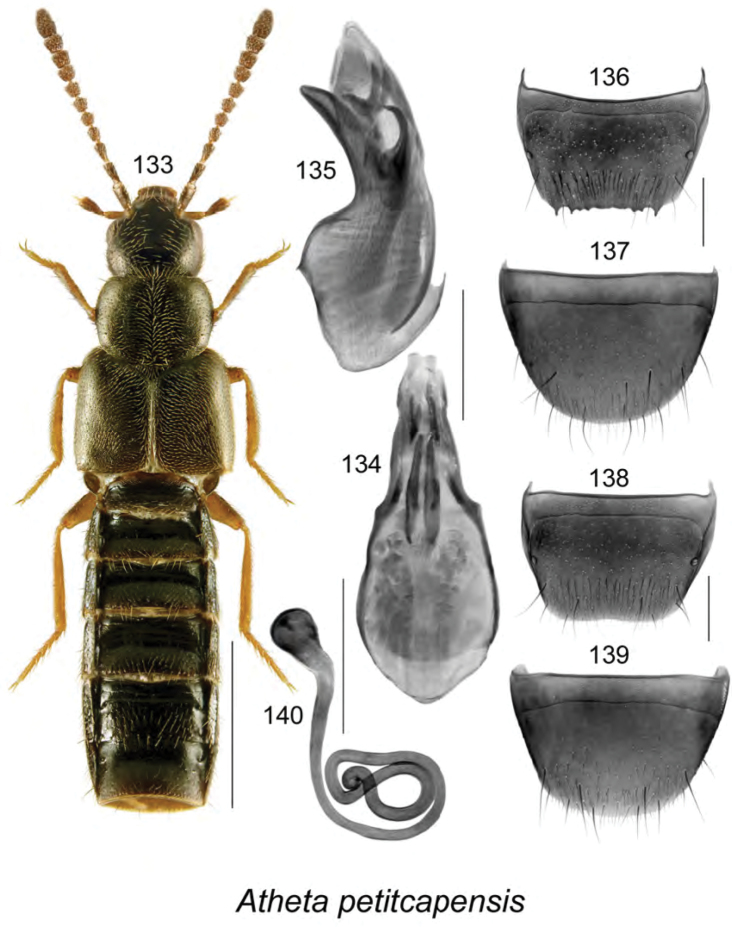
Atheta (Dimetrota) petitcapensis Klimaszewski & Webster, sp. n.: **133** habitus in dorsal view **134** median lobe of aedeagus in dorsal view **135** median lobe of aedeagus in lateral view **136** male tergite VIII **137** male sternite VIII **138** female tergite VIII **139** female sternite VIII **140** spermatheca. Scale bar of habitus = 1 mm; remaining scale bars = 0.2 mm.

Body length 2.7–2.9 mm, [narrow], narrowly elongate, broadest at elytra, dark brown to nearly black, with legs and last article of maxillary palps yellowish brown (Fig. 133); integument moderately glossy with strong meshed microsculpture; forebody with fine and dense punctation and pubescence; head elongate, rounded posterolaterally, with moderately large eyes, each about as long as postocular area; antennae with articles V–X subquadrate to moderately transverse; pronotum transverse, broadest at middle, arcuate on sides, slightly wider than head and distinctly narrower than elytra, pubescence directed laterad and apicad, forming arcuate lines from midline of disk; elytra transverse, with pubescence directed posterolaterad and forming waves posteriorly; abdomen subparallel. **Male.** Median lobe of aedeagus with bulbus narrowly oval, with apical processes angular, tubus narrowly elongate, triangular in dorsal view (Fig. 134), and slightly produced ventrally at apex, with apical part narrow and triangular in lateral view, venter broadly arcuate (Fig. 135); internal sac with two pairs of strongly sclerotized structures (Figs 134, 135); tergite VIII truncate apically and with two larger lateral teeth and several small ones between (Fig. 136); sternite VIII rounded apically (Fig. 137). **Female.** Tergite VIII truncate apically (Fig. 138); sternite VIII broadly rounded apically (Fig. 139); spermatheca with capsule small, club shaped, and with narrow and moderately deep apical invagination, stem thin and irregularly twisted posteriorly (Fig. 140).

######### Distribution.

Known only from NB, Canada.

**Natural History.** Adults of this species were found under sea wrack and grass debris on a sea beach in the upper intertidal zone.

######### Comments.

This species is superficially similar externally to species of the genus *Psammostiba* Yosii and Sawada, but has differently shaped mouth parts and genitalia. We include this species in the subgenus *Dimetrota* on the basis of body pubescence pattern, forebody punctation, and the type of genitalia.

######## Atheta (Dimetrota) sphagnicola

Taxon classificationAnimaliaColeopteraStaphylinidae

Klimaszewski & Webster sp. n.

http://zoobank.org/22C94495-44A0-40BD-8F4E-E6CFC415B283

Figs 141–145

######### Holotype (male).

**Canada, New Brunswick, York Co.**, Charters Settlement, 45.8267°N, 66.7343°W, 16.IV.2005, R.P. Webster, coll. // *Carex* marsh in *Sphagnum* hummocks (LFC). **Paratypes: Canada, New Brunswick, Queens Co.**, Upper Gagetown, bog adjacent to Hwy 2, 45.8316°N, 66.2346°W, 12.IV.2006, R.P. Webster, coll. // Tamarack bog, in sphagnum hummocks & litter at bog margin (1 ♂, RWC). **Saint John Co.**, Chance Harbour off Rt. 790, 45.1355°N, 66.3672°W, 15.V.2006, R.P. Webster, coll. // Calcareous fen, in sphagnum & litter in depression with *Carex* (1 ♂, RWC).

######### Etymology.

The specific name, *sphagnicola*, meaning “living on *Sphagnum*”, is in reference to the *Sphagnum* hummocks where the holotype was collected.

######### Description.

**Figures 141–145.**
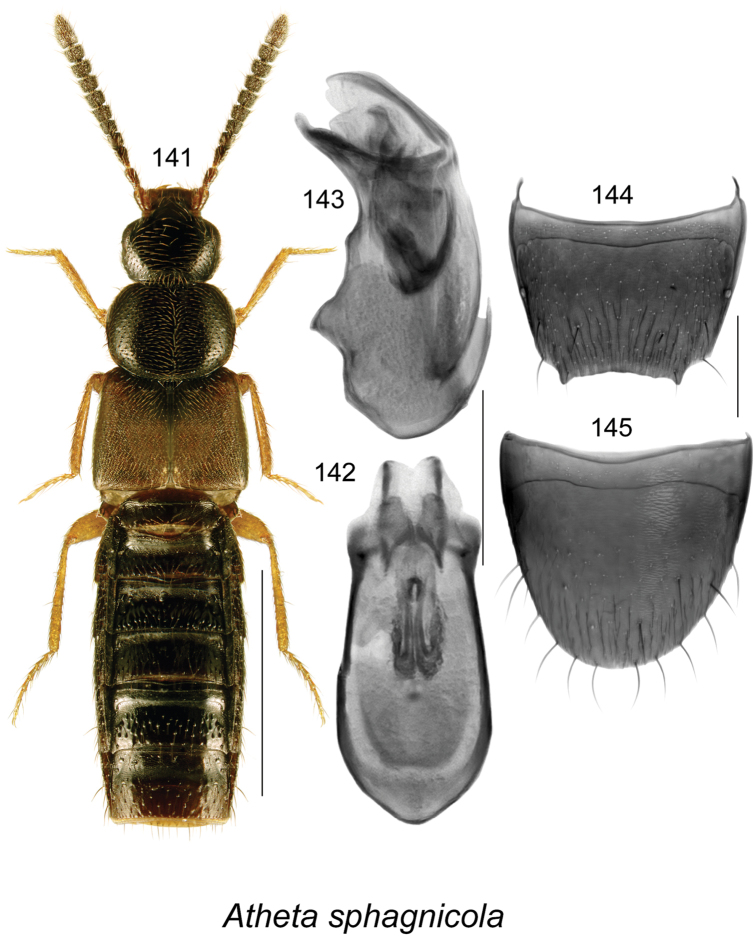
Atheta (Dimetrota) sphagnicola Klimaszewski & Webster, sp. n.: **141** habitus in dorsal view **142** median lobe of aedeagus in dorsal view **143** median lobe of aedeagus in lateral view **144** male tergite VIII **145** male sternite VIII. Scale bar of habitus = 1 mm; remaining scale bars = 0.2 mm.

Body length 3.4 mm, narrowly elongate, subparallel; head, pronotum, and abdomen dark brown to almost black, elytra yellowish reddish brown with base and scutellar area darker (Fig. 141); integument strongly glossy with strong meshed microsculpture; forebody with fine and moderately dense punctation and pubescence; head rounded and arcuate posterolaterally, with eyes moderately large, about as long as postocular area; antennae with articles V–X subquadrate to moderately transverse; pronotum transverse, rounded on sides, distinctly wider than head and narrower than elytra, pubescence directed laterad from midline of disk; elytra transverse, truncate apically, with pubescence directed posterolaterad; abdomen subparallel, narrower than elytra and arcuate laterally. **Male.** Median lobe of aedeagus with bulbus moderately narrowly oval, with rounded projections apicolaterally (Fig. 142), tubus with apical part narrowly triangular and sinuate in lateral view, venter arcuate (Fig. 143); internal sac with complex structures (Figs 142, 143); tergite VIII shallowly emarginate apically, with two small lateral teeth (Fig. 144); sternite VIII elongate and rounded apically (Fig. 145). **Female.** Unknown.

######### Distribution.

Known only from NB, Canada.

######### Natural history.

The three known individuals of this species were collected from sphagnum in a *Carex* marsh, a calcareous fen, and a tamarack bog. Adults were collected during April and May.

######### Comments.

This species is very distinct in its genital structures. It is superficially similar to Atheta (Dimetrota) venti (Lohse) in terms of having a broad tubus of the median lobe of aedeagus in dorsal view. For illustration of Atheta (Dimetrota) venti see Lohse et al. (1990).

######## Atheta (sensu lato) pseudoschistoglossa

Taxon classificationAnimaliaColeopteraStaphylinidae

Klimaszewski & Webster sp. n.

http://zoobank.org/BD630974-44D6-451E-8D1C-ACC6222858BA

Figs 146–153

######### Holotype (male).

**Canada, New Brunswick, York Co.**, Kingsclear Mazerolle Settlement, 45.8729°N, 66.8311°W, 28.IV.2006, R.P. Webster, coll. // Stream margin in grass litter on muddy soil (LFC). **Paratypes: Canada, British Columbia**, Monashee Mtn., near Cherryville, 1400–1600 m, 10.VIII.1982, leg. R. Baronowski (BWRS) (1 ♀, LUC); New Denver, 13.VIII.1982, leg. R. Baronowski (BWRS) (1 ♀, LUC); 15 km E New Denver, Zincton Summit, 13.VIII.1982, leg. R. Baronowski (BWRS) (1 ♀, LUC); **New Brunswick, Albert Co.**, Mary’s Point, 21.VIII.2003, salt marsh, C.G. Majka (1 ♀, LFC); Shepody N.W.A., Germantown Section, 45.7056°N, 64.7642°W, 17.V.2004, R.P. Webster, coll. // Cattail/sedge marsh, in marsh litter (1 ♀, NBM). **Carleton Co.**, Belleville, Meduxnekeag Valley Nature Preserve, 46.1935°N, 67.6825W, 19.IV.2005, R.P. Webster, coll. // Mixed forest, in moist leaves (1 ♂, 2 ♀, CNC); same locality but, 46.1931°N, 67.6825W, 31.V.2005, M.-A. Giguère & R. Webster, coll. // Upper river margin, under drift material (1 ♂, 1 ♀, LFC); same locality but 46.1888°N, 67.6762°W, 20.V.2005, R.P. Webster, coll. // River margin, in flood debris (1 ♂, LFC); same locality but, 46.1944°N, 67.6832°W, 2.VI.2008, R.P. Webster, coll. // River margin, under cobblestones in sand / gravel among scattered grasses (1 ♂, RWC); Jackson Falls, “Bell Forest Preserve”, 46.21456°N, 67.72056°W, 12.IV.2007, R.P. Webster, coll. // Upper river margin, in drift material in area without snow, adults very active (1 ♀, LFC; 1 ♀, NBM); same locality but, 46.2142°N, 67.7190°W, 1.VI.2005, R.P. Webster, coll. // Upper river margin, collected while [they were] in flight between 16:00 & 18:00 h (2 ♀, CNC; 1 ♂, 1 ♀, LFC). **Queens Co.**, Grand Lake near Scotchtown, 45.8762°N, 66.1817°W, 25.V.2006, R.P. Webster, coll. // Silver maple swamp near lake margin, margin of vernal pond in moist leaves (2 ♀, NBM; 1 ♂, 1 ♀, RWC); same data but 17.VI.2013 (1 ♀, RWC); same data but 5.VI.2004 // Lake margin, under drift material (1 ♂, LFC); same data but oak & maple forest, under bark of oak (1 ♀, LFC; 1 ♀, RWC); Jemseg, 45.8412°N, 66.1195°W, 25.V-12.VI.2012, C. Alderson, C. Hughes, & V. Webster // Hardwood woodland near seasonally flooded marsh, Lindgren funnel trap, 1 m high under *Quercus macrocarpa* (1 ♂, RWC); C.F.B. Gagetown, 45.7516°N, 66.1866°W, 17.VI-3.VII.2013, C. Alderson & V. Webster // Old mixed forest with *Quercus rubra*, Lindgren funnel trap in canopy of *Quercus rubra* (1 ♂, AFC). **Restigouche Co.**, Jacquet River Gorge P.N.A., 47.8200°N, 66.0015°W, 13.V.2010, R.P. Webster // Under alders in leaf litter & moss near small brook in *Carex* marsh (1 ♀, RWC). **Sunbury Co.**, Acadia Research Forest, 45.9816°N, 66.3374°W, 18.VII.2007, R.P. Webster, coll. // Road 7 Regenerating Forest, 8.5-year-old regenerating mixed forest, in sphagnum and leaf litter at bottom of old tire depression (1 ♂, RWC); Burton, near Sunpoke Lake, 45.7658°N, 66.5546°W, 3.VII.2008, R.P. Webster, coll. // red oak forest near flooded marsh, in leaf litter (2 ♀, RWC); same locality as previous but 45.7665°N, 66.5545°W, 15.V.2004, R.P. Webster, coll. // Old maple forest, in leaf litter (1 sex undetermined, 1 ♀, LFC). **York Co.**, New Maryland, Charters Settlement, 45.8267°N, 66.7343°W, 16.IV.2005, R.P. Webster, coll. // *Carex* marsh, in litter & sphagnum at base of tree (1 ♂, CNC); Rt. 645 at Beaver Brook, 45.6860°N, 66.8668°W, 6.V.2008, R.P., Webster, coll. // *Carex* marsh, in litter (rotten wood & debris) at base of dead red maple (1 ♀, RWC); 9.2 km W of Tracy off Rt. 645, 45.6837°N, 66.8809°W, 22.V.2008, R.P. Webster // *Carex* marsh adjacent to slow [flowing] stream in *Carex* hummock (1 ♀, LFC; 1 ♂, RWC); same data but 22.V.2008 (1 ♀, NBM); Fredericton, Nashwaaksis River at Rt. 105, 45.9850°N, 66.6900°W, 6.V.2006, R.P. Webster, coll. // River margin, in flood debris on upper river margin (1 ♂, LFC); Kingsclear, Mazorolle Settlement, 45.8729°N, 66.8311°W, 28.IV.2006, R.P. Webster, coll. // stream margin, in grass litter on muddy soil (1 ♂, LFC). **USA, Alaska**, -16 miles E Willow, 7.VIII.1988, leg. R. Baranowski, evening sweeping, gravel pit (1 ♂, 2 ♀, LUC). **Non-type material: USA, Alaska**, 8–16 miles E Willow, 7.VIII.1988, leg. R. Baranowski, evening sweeping gravel pit (LUC)

######### Etymology.

The specific name, *pseudoschistoglossa*, is an adjective derived from the generic name *Schistoglossa*, with the prefix *pseudo* added, reflecting the superficial similarities of this species to the members of the latter genus.

######### Description.

**Figures 146–153.**
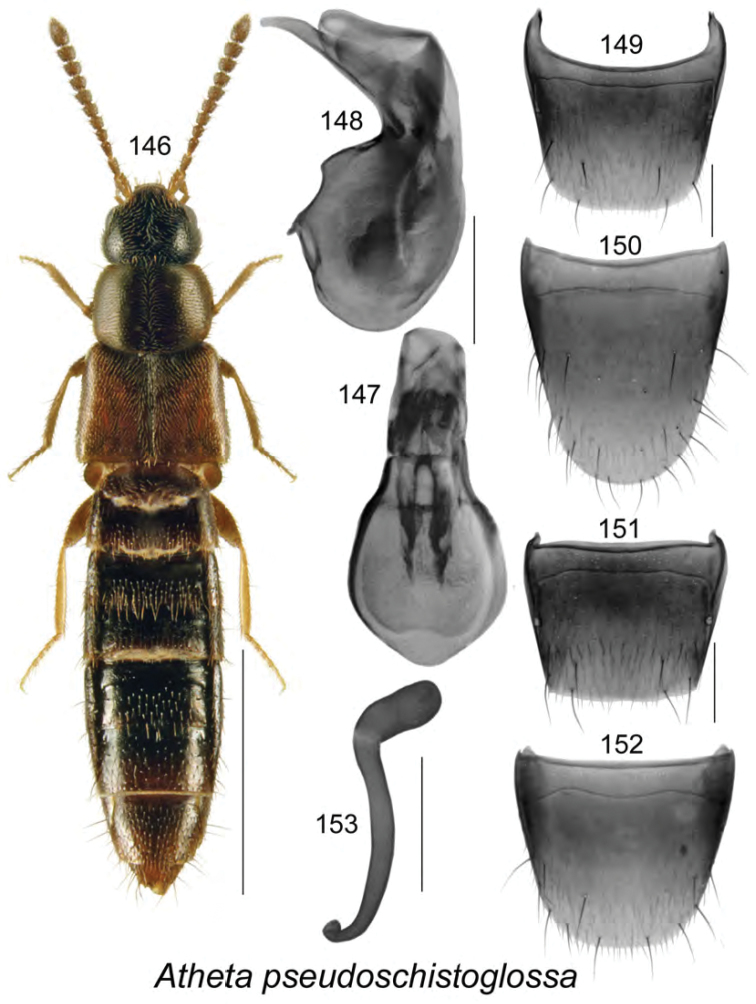
*Atheta* (*sensu lato*) *pseudoschistoglossa* Klimaszewski & Webster, sp. n.: **146** habitus in dorsal view **147** median lobe of aedeagus in dorsal view **148** median lobe of aedeagus in lateral view **149** male tergite VIII **150** male sternite VIII **151** female tergite VIII **152** female sternite VIII **153** spermatheca. Scale bar of habitus = 1 mm; remaining scale bars = 0.2 mm.

Body length 2.9 mm, narrowly elongate, subparallel; head, pronotum, and abdomen dark brown, elytra rust brown mottled with black, legs and antennae light brown (Fig. 146); integument strongly glossy; forebody with minute and sparse punctation and sparse pubescence; head rounded and slightly angular posterolaterally, with moderately large eyes, shorter than postocular area in dorsal view; antennae with articles V–X subquadrate to slightly transverse; pronotum rounded anterolaterally and posterolaterally, slightly transverse, insignificantly wider than head and slightly narrower than elytra, pubescence directed laterad from midline of disk; elytra slightly transverse, flattened, with pubescence directed posterolaterad; abdomen subparallel medially, narrower than elytra. **Male.** Median lobe of aedeagus with bulbus moderately broad, narrowly oval, tubus narrow subparallel, becoming triangular apically in dorsal view (Fig. 147), and strongly produced ventrally and with apical part narrowly elongate in lateral view (Fig. 148); internal sac with complex structures (Figs 147, 148); tergite VIII with apical margin truncate medially and broadly arcuate laterally (Fig. 149); sternite VIII strongly elongate and rounded apically (Fig. 150). **Female.** Tergite VIII with apical margin truncate (Fig. 151); sternite VIII evenly broadly rounded apically (Fig. 152); spermatheca with narrow sac-shaped capsule with weak apical indentation and sinuate stem narrowly hooked posteriorly (Fig. 153).

######### Distribution.

Known from AK, BC, and NB, most likely transcontinental in northern Canada.

######### Natural history.

Most adults of *Atheta pseudoschistoglossa* were found in or near wetland habitats. These included among cobblestones, drift material, and flood debris along river margins, moist leaves along vernal pond margin in a silver maple swamp, in leaf litter and moss along brook margins in alder swamps, and in litter at base of red maple, in *Carex* hummock in *Carex* marshes, in leaf litter in a red oak forest near a flooded seasonally flooded marsh, in a salt marsh, in marsh litter in a *Carex*–sedge marsh, and in litter and sphagnum at the base of a tree in a marsh. A few adults were captured in Lindgren funnel traps in a hardwood woodland near a seasonally flooded marsh and in an old mixed forest. Adults were collected from mid-April to August.

######### Comments.

The subgeneric position of this species is unsettled. It bears a superficial resemblance to members of the genus *Schistoglossa* but does not have the apical parts of the mandibles split. It does not belong to *Boreophilia* because of the very narrow body and different type of aedeagus and spermatheca. In *Boreophilia*, the median lobe of aedeagus is broad with the bulbus enlarged and broadly connected to tubus in dorsal view, the venter of tubus is approximately straight in lateral view, and the spermatheca is differently shaped (for illustrations of genitalia of Canadian *Borephilia* see Lohse et al. 1990). It is also similar to *Philhygra* but it has a large spermatheca similar in shape to those of *Schistoglossa*, whereas *Philhygra* have spermathecae that are minute, scarcely visible, and difficult to find. One specimen from AK agrees in all aspects of morphology with those from NB but is distinctly larger and therefore it is listed as a non-paratype.

######## Atheta (sensu lato) thujae

Taxon classificationAnimaliaColeopteraStaphylinidae

Klimaszewski & Webster sp. n.

http://zoobank.org/48A53E40-4216-49F5-9BBF-A5AFA9F251AA

Figs 154–160

######### Holotype (male).

**Canada, New Brunswick, Charlotte Co.**, 10 km NW of New River Beach, 45.2110 N, 66.6170°W, 17–31.V.2010, R. Webster & C. MacKay, coll. // old growth Eastern White Cedar forest, Lindgren funnel trap (LFC). **Paratypes**: **Canada, New Brunswick, Carleton Co.**, Jackson Falls, “Bell Forest”, 46.2200°N, 67.7231°W, 12–19.VI.2008, R.P. Webster, coll. // Rich Appalachian hardwood forest with some conifers, Lindgren funnel trap (1 ♂, RWC). **Charlotte Co.**, 10 km NW of New River Beach, 45.2110 N, 66.6170°W, 30.IV-17.V.2010, 17–31.V.2010, R. Webster & C. MacKay, coll. // old growth Eastern White Cedar forest, Lindgren funnel traps (4 ♀, RWC). **York Co.**, Charters Settlement, 45.8395°N, 66.7391°W, 26.V.2008, R.P. Webster coll. // mixed forest, in decaying moldy corncobs and cornhusks (1 ♀, LFC).

######### Etymology.

The specific name, *thujae*, is an adjective derived from the generic name *Thuja*, in reference to the dominant tree species, *Thuja occidentalis* L., where the holotype and most paratypes were collected.

######### Description.

**Figures 154–160.**
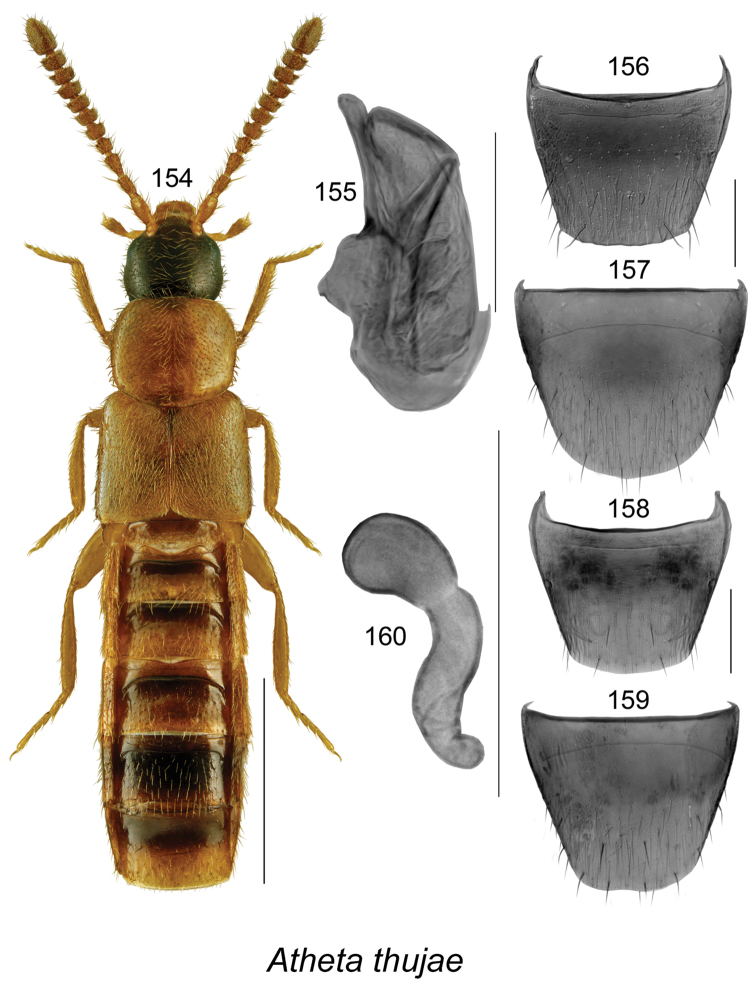
*Atheta* (*sensu lato*) *thujae* Klimaszewski & Webster, sp. n.: **154** habitus in dorsal view **155** median lobe of aedeagus in lateral view **156** male tergite VIII **157** male sternite VIII **158** female tergite VIII **159** female sternite VIII **160** spermatheca. Scale bar of habitus = 1 mm; remaining scale bars = 0.2 mm.

Body length 2.9–3.0 mm, narrowly subparallel; head, posterior part of abdomen, impressions of abdominal tergites, and medioapical parts of antennae dark brown, with remainder of body yellowish (Fig. 154); integument moderately glossy except strongly so on abdomen, with distinct meshed microsculpture; head slightly narrower than pronotum, elongate, gradually narrowed basally from posterior margin of eyes, eyes small, postocular area long and at least twice as long as diameter of eye; antennae with article V subquadrate and VI–X moderately to strongly transverse; pronotum slightly narrower than elytra, approximately rectangular, with sharp lateral margin, pubescence directed obliquely laterad from midline of disk; elytra slightly transverse with pubescence directed posteriad; abdomen subparallel with deep basal impression on first three visible tergites. **Male.** Median lobe of aedeagus with bulbus broad, oval, tubus short, triangular in dorsal view, short and straight in lateral view (Fig. 155); internal sac structures not apparent; tergite VIII with apex truncate, bearing traces of crenulation (Fig. 156); sternite VIII rounded apically (Fig. 157). **Female.** Tergite and sternite VIII arcuate apically (Figs 158, 159); spermatheca small with spherical capsule and short sinuate stem (Fig. 160).

######### Distribution.

Known only from NB, Canada.

######### Natural history.

Specimens were captured in Lindgren funnel traps in an old-growth eastern white cedar forest, a rich Appalachian hardwood forest with some conifers, and from decaying moldy corncobs and cornhusks in a mixed forest. Adults were collected during May and June.

######### Comments.

This species is unique in the shape of its genitalic features, and there are no closely related species as far as we know.

######## Atheta (Pseudota) klagesi

Taxon classificationAnimaliaColeopteraStaphylinidae

Bernhauer, 1909

Figs 161–169

Atheta (Pseudota) klagesi (For diagnosis, see Bernhauer 1909, Gusarov 2003)

######### Lectotype (male).

USA, Maine, Frost, 1654; 153; *klagesi* Brh., Cotypus; Fenyes; Chicago NHMus, M. Bernhauer Collection; FMNH 281916; Lectotype teste D.J. Clarke 2014, GDI Imaging Project; V.I. Gusarov paralectotype designation label 2000; designated by Gusarov 2003 (FMNH). **Paralectotypes**: Data same as for holotype (FMNH) 1 female; USA, Pennsylvania, Jeannette, H.G. Klages; *klagesi* Bernhauer, Typus, Fenyes; Chicago NHMus., M. Bernhauer Collection; lectotype designation label by V.I. Gusarov 2000 designated by Gusarov 2003 (FMNH).

######### Diagnosis.

**Figures 161–169.**
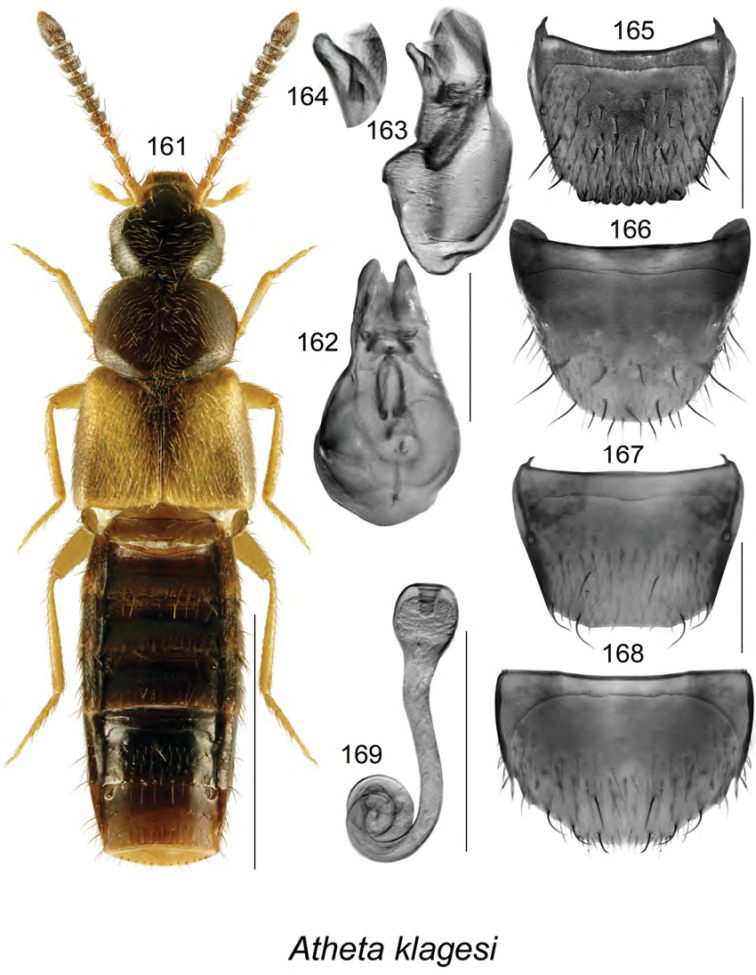
Atheta (Pseudota) klagesi Bernhauer: **161** habitus in dorsal view **162** median lobe of aedeagus in dorsal view **163** median lobe of aedeagus in lateral view **164** enlarged apical part of tubus in lateral view **165** male tergite VIII **166** male sternite VIII **167** female tergite VIII **168** female sternite VIII **169** spermatheca **162–166** based on lectotype. Scale bar of habitus = 1 mm; remaining scale bars = 0.2 mm.

*Atheta klagesi* is very similar to the next species, *Atheta pseudoklagesi*, and may be distinguished from it by the following combination of characters: body slightly smaller in size and more glossy, yellowish areas on elytra more intense, coloration of legs, bases of antennae and maxillary palps more intense yellowish, and overall body color more contrasting (Fig. 161); median lobe of aedeagus with tubus shorter, apex more arcuate and with slightly different shape (Figs 162–164); spermatheca very similarly shaped; females may be difficult to identify unless collected with males.

######### Distribution in Canada and Alaska.

Currently recorded from YT, BC, AB, SK, ON, QC, NB, NS, PE, LB and NF (Bousquet et al. 2013), but some of these may prove to be undetected specimens of *Atheta pseudoklagesi*.

######## Atheta (Pseudota) pseudoklagesi

Taxon classificationAnimaliaColeopteraStaphylinidae

Klimaszewski & Webster sp. n.

http://zoobank.org/7B5EE640-B0DF-49FA-B1B4-3FFB9D116FCD

Figs 170–177

######### Holotype (male).

**Canada, New Brunswick, York Co**., New Maryland, Charters Settlement, 45.8340°N, 66.7450°W, 11.VIII.2007, R.P. Webster, coll. // Mature mixed forest, in coral fungi on *Populus* log (LFC). **Paratypes: Canada, New Brunswick, Restigouche Co.**, Dionne Brook P.N.A., 47.9064°N, 68.3441°W, 23.VIII.2011, R.P. Webster // Old-growth white spruce & balsam fir forest, in decaying gilled mushroom (1 ♂, 3 ♀, RWC); same locality as previous but 31.V-15.VI.2011, 27.VI-14.VII.2011, M. Roy & V. Webster, coll. // Old-growth white spruce & balsam fir forest, flight intercept traps (1 ♂, 3 ♀, RWC); off Bellone Road, 47.7755°N, 68.2501°W, 24.VIII.2011, R.P. Webster & M. Turgeon // Old spruce & fir forest, mossy forest floor, in gilled mushrooms of various stages of decay (1 ♂, RWC). **Sunbury Co.**, Acadia Research Forest, 45.9799°N, 66.3394°W, 18.IX.2007, R.P. Webster, coll. // Road 7 control, mature red spruce & red maple forest, in gilled mushroom (1 ♂, RWC). **York Co**., New Maryland, Charters Settlement, 45.8286°N, 66.7365°W, 3.VI.2007, R.P. Webster, coll. // Mature red spruce forest, under bark of red spruce (1 ♂, RWC); 8.4 km W of Tracy, off Rt 645, 45.6821°N, 66.7894°W, 6.V.2008, R.P. Webster coll. // wet alder swamp, in fleshy polypore fungi base of dead standing *Populus* sp. (1 ♂, 1 ♀, CNC).

######### Etymology.

The name of this species derives from the species name *klagesi* and the prefix *pseudo*-, false, in allusion to its similarity to that species.

######### Description.

**Figures 170–177.**
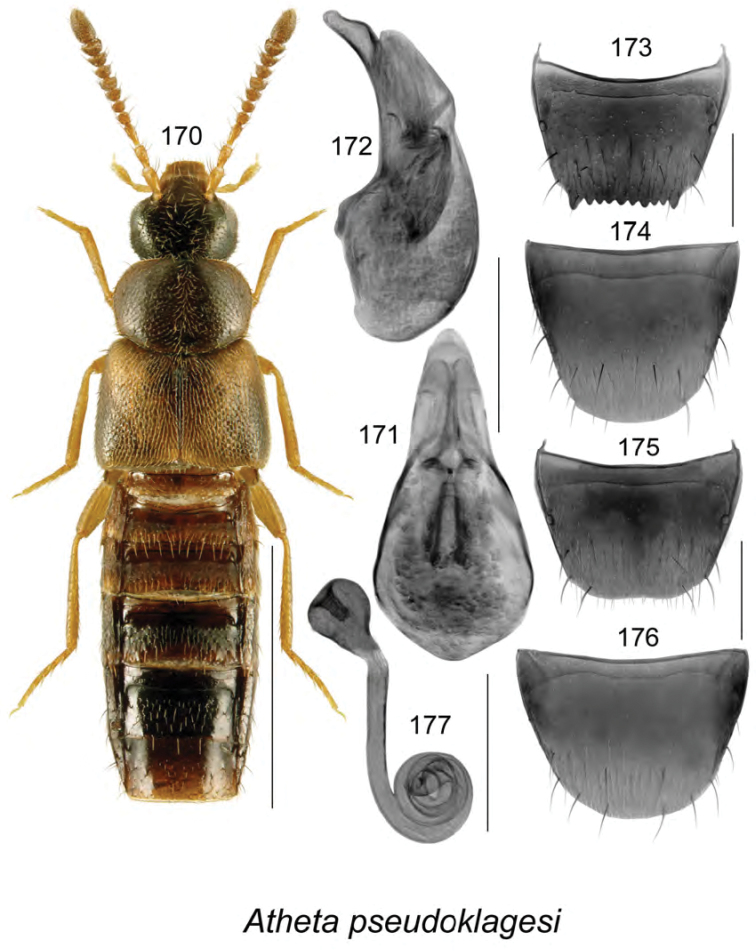
Atheta (Pseudota) pseudoklagesi Klimaszewski & Webster, sp. n.: **170** habitus in dorsal view **171** median lobe of aedeagus in dorsal view **172** median lobe of aedeagus in lateral view **173** male tergite VIII **174** male sternite VIII **175** female tergite VIII **176** female sternite VIII **177** spermatheca. Scale bar of habitus = 1 mm; remaining scale bars = 0.2 mm.

Body length 2.6–2.8 mm, narrowly oval; head, pronotum, and posterior part of abdomen dark brown to nearly black, elytra dark brown with two oblique yellowish-brown bands, each ranging from shoulder to lower elytral suture; legs, bases of antennae, maxillary palpi, and often basal part of abdomen yellowish brown (Fig. 170); integument strongly glossy with meshed microsculpture; forebody with punctation and pubescence minute and dense, less so on head; head rounded posterolaterally, with moderately large eyes, each about as long as postorbital area; antennae with articles V–X subquadrate to strongly transverse; pronotum arcuate laterally, broadest just anterior of middle of its length, slightly transverse, distinctly wider than head and distinctly narrower than elytra, pubescence directed laterad from midline of disk; elytra strongly transverse, with pubescence directed posterolaterad; abdomen subparallel, narrower than elytra. **Male.** Median lobe of aedeagus with bulbus moderately broad, narrowly oval, tubus narrowly elongate, triangular in dorsal view (Fig. 171), and long, straight for most of its length, with apical part strongly produced ventrally in lateral view (Fig. 172); internal sac with weak structures (Figs 171, 172); tergite VIII with apical margin truncate and serrate (Fig. 173); sternite VIII rounded apically (Fig. 174). **Female.** Tergite VIII with apical margin truncate (Fig. 175); sternite VIII broadly rounded apically (Fig. 176); spermatheca with narrow bulbus capsule and deep apical indentation, stem long, narrow, and coiled posteriorly (Fig. 177).

This is a sibling species of *Atheta klagesi* and was confused with the latter in collections. It may be distinguished from *Atheta klagesi* by the following combination of characters: size slightly larger, body less glossy, legs, bases of antennae, maxillary palps and bands on elytra less intensely yellowish in coloration, body color less contrasting overall; median lobe of aedeagus with tubus longer, with apex shaped slightly differently in lateral view; spermatheca very similarly shaped in the two species, and females may be difficult to identify without accompanying males.

######### Distribution.

Currently known only from NB, Canada, but because of confusion with *Atheta klagesi*, this species will undoubtedly prove to be more widespread.

######### Natural history.

Adults of this species were found in mature mixed forest, old-growth and old white spruce and balsam fir forests, a mature red spruce forest, and in a wet alder swamp. Specimens were collected from coral fungi on a *Populus* log, fleshy polypore fungi at base of a dead standing *Populus*, in decaying gilled mushrooms, in gilled mushrooms, and under bark of red spruce. Adults were collected from May to September.

######### Comments.

In the past, the two sibling species were mixed together and identified as *Atheta klagesi*. All material across Canada needs to be reexamined to understand the true distribution of the two species. In this paper, only NB specimens were reevaluated.

######## Dinaraea curtipenis

Taxon classificationAnimaliaColeopteraStaphylinidae

Klimaszewski & Webster, 2013

Figs 178–184

Dinaraea curtipenis (For diagnosis, see Klimaszewski et al. 2013b)

######### Material examined.

**Additional New Brunswick record. Restigouche Co.**, Dionne Brook P.N.A., 47.9064°N, 68.3441°W, 31.V-15.VI.2011, M. Roy & V. Webster, coll. // Old-growth white spruce & balsam fir forest, Lindgren funnel trap (1 ♀, RWC).

######### Distribution in Canada and Alaska.

NB (Klimaszewski et al. 2013b).

######### Comments.

Several females originally thought to possibly be *Dinaraea curtipenis* (Klimaszewski et al. 2013b) were later determined to be *Dinaraea subdepressa* (Bernhauer). However, we found another specimen from NB that proved to be a female of *Dinaraea curtipenis*. All external characters agree with those of the males. Here, we illustrate the female spermatheca, tergite, and sternite VIII for the first time (Figs 182–184).

**Figures 178–184.**
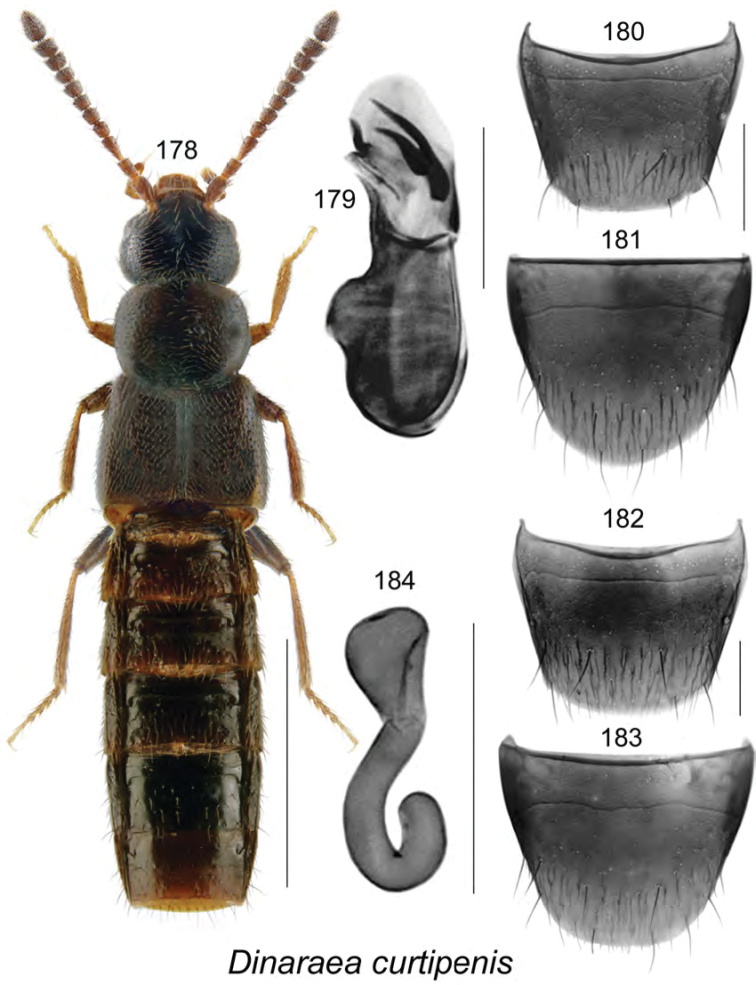
*Dinaraea curtipenis* Klimaszewski & Webster: **178** habitus in dorsal view **179** median lobe of aedeagus in lateral view **180** male tergite VIII **181** male sternite VIII **182** female tergite VIII **183** female sternite VIII **184** spermatheca. Scale bar of habitus = 1 mm; remaining scale bars = 0.2 mm.

######## Dinaraea longipenis

Taxon classificationAnimaliaColeopteraStaphylinidae

Klimaszewski & Webster, 2013

Figs 185–191

Dinaraea longipenis (For diagnosis, see Klimaszewski et al. 2013b)

######### Material examined.

**Additional New Brunswick records. York Co.**, 15 km W of Tracy, off Rt. 645, 45.6848°N, 66.8821°W, 26.IV-10.V.2010, R. Webster & C. MacKay, coll. // Old red pine forest, Lindgren funnel trap (1 ♀, RWC); Canterbury, Eel River P.N.A., 45.8966°N, 66.6345°W, 2–20.VI.2014, C. Alderson & V. Webster // Old-growth eastern white cedar swamp & fen, Lindgren funnel trap (1 ♀, LFC).

######### Distribution in Canada and Alaska.

NB (Bousquet et al. 2013; Klimaszewski et al. 2013b).

######### Comments.

A female externally very similar to males of *Dinaraea longipenis* was mentioned by Klimaszewski et al. (2013b) but was not included in the type series or description because of close similarity to specimens of *Dinaraea piceana* Klimaszewski & Jacobs. During 2014, we collected another female that is identical to the one mentioned above. After comparison with *Dinaraea piceana*, we concluded that these females are *Dinaraea longipenis*. *Dinaraea piceana* differs externally from *Dinaraea longipenis* in possessing stronger microsculpture on the pronotum and elytra (appears matte), with brighter coloration. Here, we illustrate the female spermatheca, tergite, and sternite VIII of *Dinaraea longipenis* for the first time (Figs 189–191).

**Figures 185–191.**
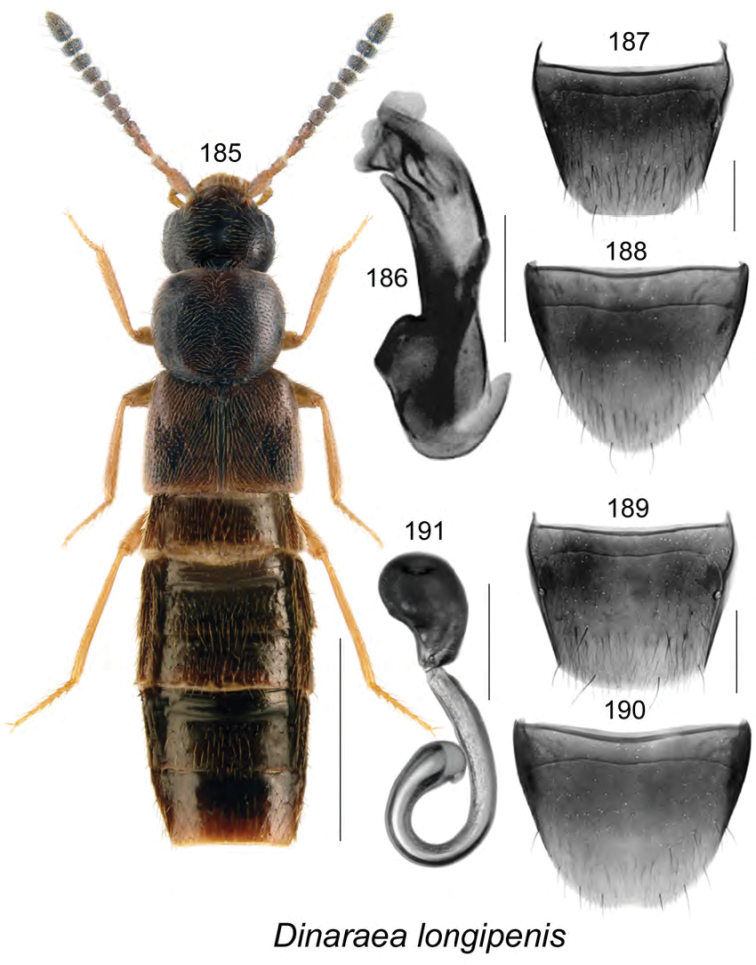
*Dinaraea longipenis* Klimaszewski & Webster: **185** habitus in dorsal view **186** median lobe of aedeagus in lateral view **187** male tergite VIII **188** male sternite VIII **189** female tergite VIII **190** female sternite VIII **191** spermatheca. Scale bar of habitus = 1 mm; remaining scale bars = 0.2 mm.

######## Dinaraea subdepressa

Taxon classificationAnimaliaColeopteraStaphylinidae

(Bernhauer, 1907)

Figs 192–198

Dinaraea subdepressa (For diagnosis, see Klimaszewski et al. 2013b)

######### Material examined.

**New Brunswick, Charlotte Co.**, 10 km NW of New River Beach, 45.2110°N, 66.6170°W, 17–31.V.2010, R. Webster & C. MacKay, coll. // Old-growth eastern white cedar forest, Lindgren funnel trap (1 ♀, LFC). **Northumberland Co.**, ca. 2.5 km W of Sevogle, 47.0876°N, 65.8613°W, 11–26.VI.2013, 27.VIII-4.IX.2013, 27.V-11.VI.2014, C. Alderson & V. Webster // Old jack pine forest, Lindgren funnel traps (1 ♂ 1 ♀, LFC; 1 ♂, 2 ♀, RWC); Upper Graham Plains, 47.1001°N, 66.8154°W, 28.V-10.VI.VII.2014, C. Alderson & V. Webster // Old black spruce forest, Lindgren funnel trap (1 ♀, RWC). **Queens Co.**, Cranberry Lake P.N.A., 46.1125°N, 65.6075°W, 3–13.V.2011, 7–22.VI.2011, M. Roy & V. Webster // Red oak forest, Lindgren funnel traps (1 ♀, LFC, 2 ♀, RWC). **Restigouche Co.**, Jacquet River Gorge P.N.A., 47.8257°N, 66.0764°W, 29.V-10.VI.2014, C. Alderson & V. Webster // Old *Populus balsamifera* stand near river, Lindgren funnel trap 1 m high under trees (2 ♂, RWC). **York Co.**, 15 km W of Tracy, off Rt. 645, 45.6848°N, 66.8821°W, 4–16.VI.2010, R. Webster & C. MacKay, coll. // Old red pine forest, Lindgren funnel trap (1 ♀, RWC); Keswick Ridge, 45.9962°N, 66.8781°W, 22.V-4.VI.2014, C. Alderson & V. Webster // Mixed forest, Lindgren funnel trap, 1 m high under trees (1 ♀, RWC).

######### Natural history.

All specimens of *Dinaraea subdepressa* from NB were captured in Lindgren funnel traps in the following forest types: an old jack pine forest, a red pine forest, an old-growth eastern white cedar forest, an old black spruce forest, mixed forests, a red oak forest, and an old balsam poplar forest near a river. Little is known about the biology and microhabitat requirements of this species. Other members of the genus live in subcortical habitats and may play a role as natural enemies of bark beetles and other subcortical insects (Klimaszewski et al. 2013b). This species presumably has a similar biology.

######### Distribution in Canada and Alaska.

**NB (New Canadian record).**

######### Comments.

**Figures 192–198.**
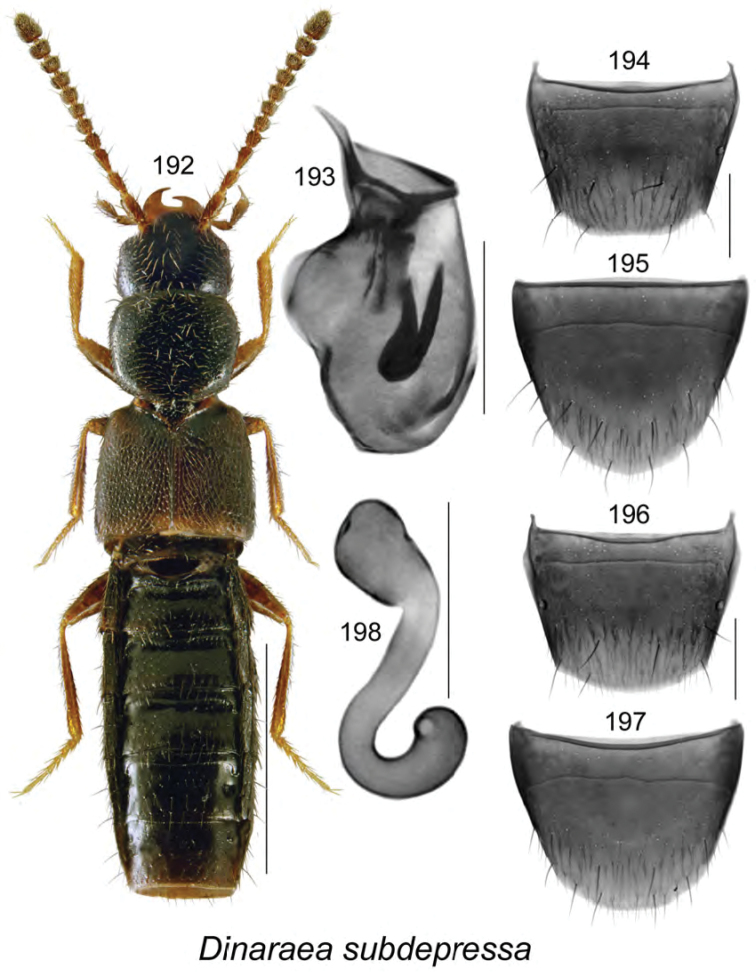
*Dinaraea subdepressa* (Bernhauer): **192** habitus in dorsal view **193** median lobe of aedeagus in lateral view **194** male tergite VIII **195** male sternite VIII **196** female tergite VIII **197** female sternite VIII **198** spermatheca. Scale bar of habitus = 1 mm; remaining scale bars = 0.2 mm.

*Dinarea subdepressa* (Bernhauer) was previously known only from NH in the USA (Bernhauer 1907). Females were previously unknown and are illustrated for the first time in this publication (Figs 196–198). This species is externally very similar to *Dinaraea curtipenis* Klimaszewski & Webster but differs in having the posterolateral angles of the pronotum very sharp, with the margin strongly depressed from the angle to the middle of the base, forming a groove (Fig. 192). In *Dinaraea curtipenis*, the posterior angle is rounded and the margin is not strongly depressed (Fig. 178).

######## Paragoniusa myrmicae

Taxon classificationAnimaliaColeopteraStaphylinidae

Maruyama & Klimaszewski, 2004

Figs 199–205

Paragoniusa myrmicae (For diagnosis, see Maruyama and Klimaszewski 2004, 2006)

######### Material examined.

**New Brunswick, Restigouche Co.**, ca. 3 km SE of Simpsons Field, 47.5277°N, 66.5142°W, 25.VI-10.VII.2015, C. Alderson & V. Webster // Old cedar & spruce forest with *Populus balsamifera* & *Populus tremuloides*, Lindgren funnel trap (1 ♀, RWC).

######### Natural history.

Females of this myrmecophilous species were collected from nests of *Myrmica alaskensis* Wheeler, the only known host ant species (Maruyama and Klimaszewski 2004, 2006). Additional specimens were collected from window traps and a pitfall trap in a burned forest (Maruyama and Klimaszewski 2006, Klimaszewski et al. 2011). The specimen from NB was captured in a Lindgren funnel trap in an old cedar and spruce forest with *Populus balsamifera* & *Populus tremuloides*.

######### Distribution in Canada and Alaska.

BC, AB, QC, **NB**, LB (Bousquet et al. 2013).

**Figures 199–205.**
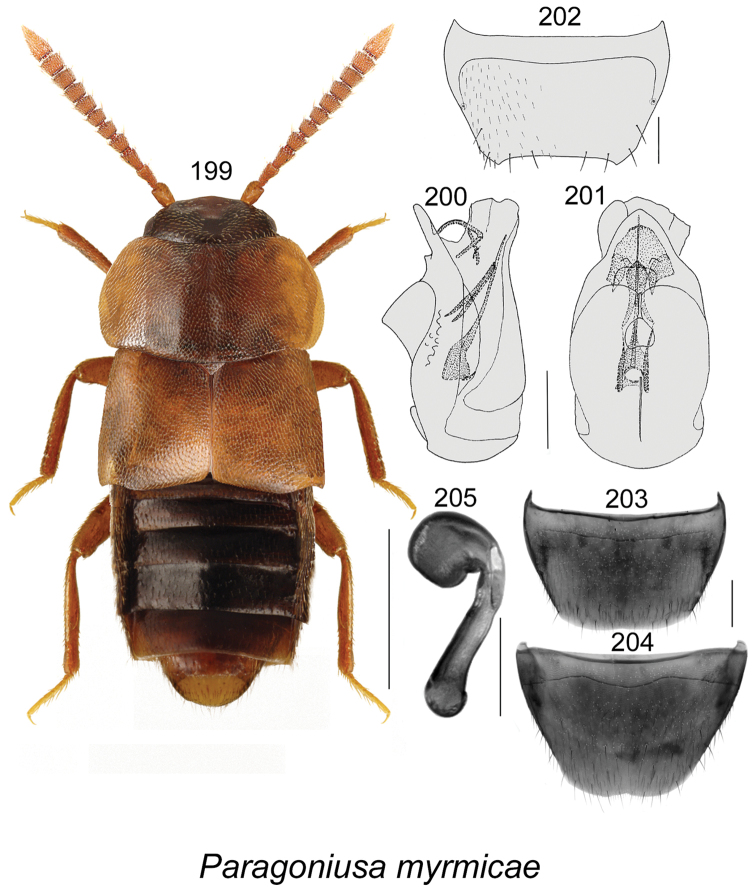
*Paragoniusa myrmicae* Maruyama & Klimaszewski: **199** habitus in dorsal view **200** median lobe of aedeagus in lateral view **201** aedeagus in ventral view **202** male tergite VIII **203** female tergite VIII **204** female sternite VIII **205** spermatheca **200–201** modified from Maruyama and Klimaszewski (2006). Scale bar of habitus = 1 mm; remaining scale bars = 0.2 mm.

######## Philhygra atypicalis

Taxon classificationAnimaliaColeopteraStaphylinidae

Klimaszewski & Webster sp. n.

http://zoobank.org/D62A01FC-8C9C-4F10-94A4-A625E780DA3C

Figs 206–218

######### Holotype (male).

**Canada, New Brunswick, Queens Co.**, C.F.B. Gagetown, 45.7516°N, 66.1866°W, 16.VIII.2013, R.P. Webster // Old mixed forest with *Quercus rubra*, in decaying mushroom (LFC). **Paratypes: Canada, New Brunswick, Northumberland Co.**, ca. 2.5 km W of Sevogle, 47.0876°N, 65.8613°W, 21.VIII.2013, R.P. Webster // Old jack pine forest, in rotten *Boletus* mushroom (1 ♀, RWC); same data except 27.VIII.2013 // In rotten gilled mushroom (1 ♂, 2 ♀, LFC; 1 ♂, RWC). **Queens Co.**, C.F.B. Gagetown, 45.7516°N, 66.1866°W, 31.VII.2013, R.P. Webster // Old mixed forest with *Quercus rubra*, in decaying mushroom (1 ♂, RWC); same data except 16.VIII.2013 (1 ♀, LFC; 1 ♀, RWC); same data except 28.VIII.2013 (1 ♂, LFC; 2 ♂, RWC).

######### Etymology.

*Atypicalis* is a Latin adjective meaning not typical, in reference to the atypical shape of the median lobe of the aedeagus of this species, for *Philhygra*.

######### Description.

**Figures 206–218.**
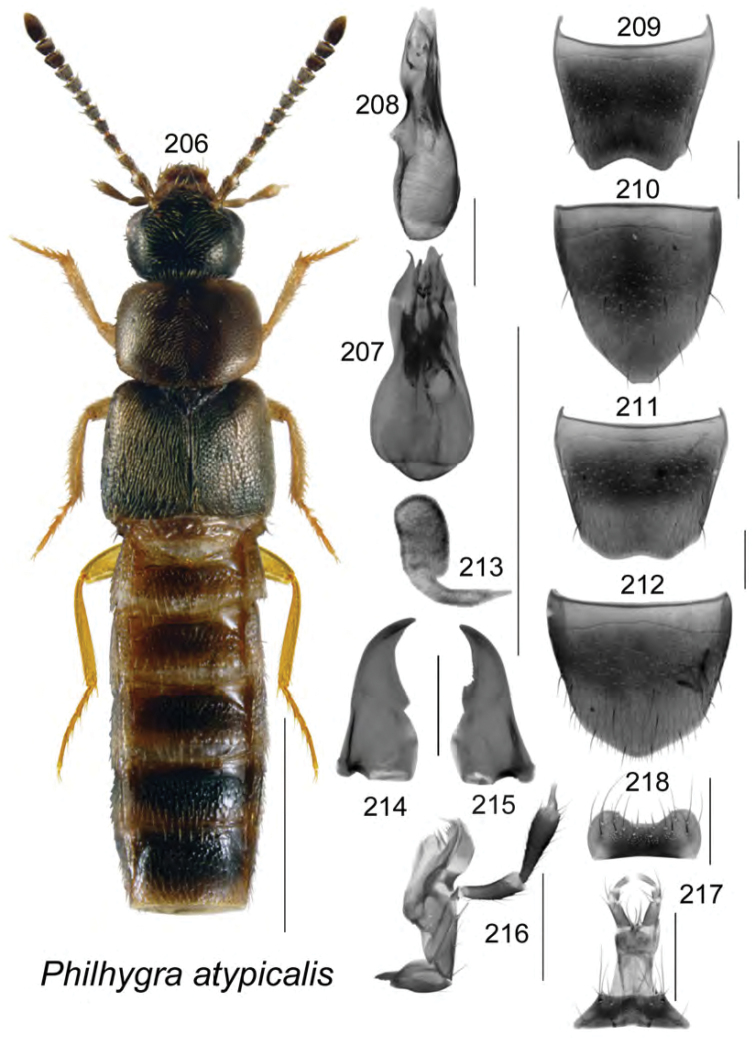
*Philhygra atypicalis* Klimaszewski & Webster, sp. n.: **206** habitus in dorsal view **207** median lobe of aedeagus in dorsal view **208** median lobe of aedeagus in lateral view **209** male tergite VIII **210** male sternite VIII **211** female tergite VIII **212** female sternite VIII **213** spermatheca **214, 215**, mandibles **216** maxilla **217** mentum and labium **218** labrum. Scale bar of habitus = 1 mm; remaining scale bars = 0.2 mm.

Body length 3.2 mm, narrow, subparallel, antennae, head, pronotum, and posterior abdomen dark brown, legs and elytra rust brown, latter mottled with black (Fig. 206); integument moderately glossy; forebody with minute and dense punctation and dense pubescence; head rounded and slightly angular posterolaterally, eyes large, mouthparts as illustrated (Figs 214–218); antennae with articles 5–10 subquadrate to slightly transverse; pronotum transverse, slightly wider than head and about as wide as elytra, with all angles moderately narrowly rounded, pubescence directed obliquely posteriad from midline of disk; elytra slightly transverse, with pubescence directed posterolaterad; abdomen subparallel, narrower than elytra. **Male.** Median lobe of aedeagus with bulbus narrowly oval, tubus moderately wide, triangular in dorsal view (Fig. 207), sinuate ventrally with apical part narrowly elongate in lateral view (Fig. 208); internal sac with complex structures (Figs 207, 208); tergite VIII deeply and broadly emarginate apically (Fig. 209); sternite VIII with apical margin strongly produced to narrowly truncate apex (Fig. 210). **Female.** Tergite VIII with apical margin shallowly emarginate in middle one-third (Fig. 211); sternite VIII slightly produced and obtusely angulate apically (Fig. 212); spermatheca with short sac-shaped capsule without apical invagination and with short and narrow stem (Fig. 213).

######### Distribution.

Known only from NB, Canada.

######### Natural history.

Adults of *Philhygra atypicalis* were collected from rotten bolete mushrooms in an old jack pine forest and from decaying mushrooms in an old mixed forest with *Quercus rubra*. Specimens were collected during July and August.

######### Comments.

*Philhygra atypicalis* externally agrees with all characteristics of the genus *Philhygra* but does not have the typical shape of the median lobe of the aedeagus (Figs 207, 208). In typical forms, the median lobe has an unusually enlarged tubus of complex forms. Interestingly, all specimens of this species were found among decaying mushrooms, an atypical habitat for *Philhygra*, which are typically associated with wetland and riparian habitats.

######## Philhygra hygrotopora

Taxon classificationAnimaliaColeopteraStaphylinidae

(Kraatz, 1856)

Figs 219–225

Philhygra hygrotopora (For description, see Strand and Vik 1964)

######### Material examined.

**New Brunswick, Carleton Co.**, Jackson Falls, 46.2257°N, 67.7437°W, 12.IX.2009, R.P. Webster, coll. // River margin near waterfall, splashing moss near splash zone of waterfall (1 ♂, RWC); Belleville, Meduxnekeag Valley Nature Preserve, 46.1897°N, 67.6761°W, 31.VII.2009, R.P. Webster, coll. // Rich Appalachian Hardwood Forest, in gravel on margin of shaded spring-fed brook near small waterfall (1 ♂, RWC). **Madawaska Co.**, Gagné Brook at First Lake, 47.6077°N, 68.2534°W, 23.VI.2010, M. Turgeon & R. Webster // northern hardwood forest, shaded brook, among gravel on gravel bar, splashing and turning gravel (1 ♂, RWC). **Restigouche Co.**, Jacquet River Gorge P.N.A., 47.8010°N, 66.0968°W, 14.VIII.2010, R.P. Webster // Cold shaded brook, in gravel (1 ♀, RWC). **Saint John Co.**, Saint John, Taylor’s Island, 45.2238°N, 66.1265°W, 24.VIII.2004, R.P. Webster, coll. // Sea beach, under decaying seaweed (1 ♂, 1 ♀, LFC; 1 ♂, RWC).

######### Diagnosis.

**Figures 219–225.**
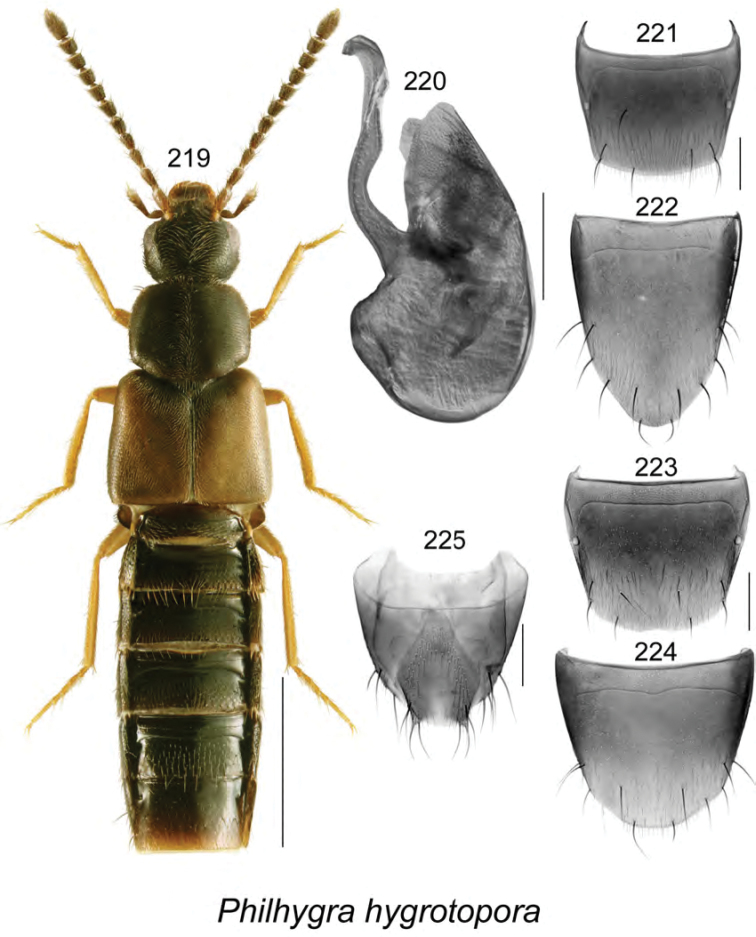
*Philhygra hygrotopora* (Kraatz): **219** habitus in dorsal view **220** median lobe of aedeagus in lateral view **221** male tergite VIII **222** male sternite VIII **223** female tergite VIII **224** female sternite VIII **225** female pygidium. Scale bar of habitus = 1 mm; remaining scale bars = 0.2 mm.

Body length 3.4 mm, narrow, subparallel; antennae, head, pronotum, and abdomen dark brown, legs and elytra yellowish brown (Fig. 219); integument not glossy; forebody with minute and dense punctation and dense pubescence; head rounded posterolaterally, with large eyes; antennae with articles V–X slightly elongate to subquadrate; pronotum transverse, slightly wider than head and slightly narrower than elytra, rounded anteriorly, with slight indentations posterolaterally, making the hind angles appear more angular, pubescence directed laterad on arcuate lines from midline of disk; elytra slightly transverse, with pubescence directed posterolaterad in waves; abdomen subparallel, narrower than elytra. **Male.** Median lobe of aedeagus with bulbus large, oval, tubus narrow, long and sinuate in lateral view (Fig. 220); internal sac with complex structures (Fig. 220); tergite VIII truncate apically (Fig. 221); sternite VIII parabolic (Fig. 222). **Female.** Tergite VIII with apical margin shallowly emarginate medially (Fig. 223); sternite VIII slightly produced apically (Fig. 224); spermatheca very small and not illustrated; pygidium as in Fig. 225.

######### Natural history.

In NB, *Philhygra hygrotopora* were found by splashing moss near the splash zone of a waterfall, in gravel on the margin of a shaded spring-fed brook near a waterfall, among gravel on a gravel bar along a shaded brook in a northern hardwood forest, and in gravel along a cold shaded brook. A few individuals were found under decaying seaweed on a sea beach. Adults were collected during June, July, August, and September.

######### Distribution in Canada and Alaska.

**NB (New North American record).** This is the first record of this species in North America.

######### Comments.

It is unclear if this is an adventive species in North America or a Holarctic one. The habitats that this species was found in are rarely sampled in North America and are not typical for adventive species.

######## Philhygra larsoni

Taxon classificationAnimaliaColeopteraStaphylinidae

Klimaszewski & Langor, 2011

Figs 226–233

Philhygra larsoni (For diagnosis, see Klimaszewski et al. 2011)

######### Material examined.

**New Brunswick, Albert Co.**, Caledonia Gorge P.N.A., 45.7985°N, 64.7755°W, 18.VIII.2012, R.P. Webster // Crooked Creek near Caledonia Brook, splashing sun-exposed moss covered rocks (1 ♂, NBM). **Charlotte Co.**, near New River, 45.21176°N, 66.61790°W, 7.VII.2006, R.P. Webster, coll. // Mixed forest, margin of small pond, treading *Carex* hummock into water (1 ♂, NBM). **Kings Co.**, Rt. 102 near Mill Brook, 45.5993°N, 66.0583°W, 13.V.2008, R.P. Webster, coll. // Red oak forest, in leaf litter near brook (1 ♂, RWC). **Queens Co.**, Cambridge, W of Jemseg at “Trout Creek” 45.8227°N, 66.1240°W, 9.V.2004, R.P. Webster, coll. // Silver maple swamp, sifting litter at base of large tree (2 ♂, NBM); same locality but 45.8255°N, 66.1174°W, 1.VII.2008, R.P. Webster, coll. // Seasonally flooded marsh, treading vegetation near pond margin (1 ♂, RWC); Canning, Grand Lake near Scotchtown, 45.8762°N, 66.1816°W, 12.V.2004, R.P. Webster, coll. // Lake shore, under drift material (2 ♂, RWC); Bayard near Nerepis River, 45.4442°N, 66.3292°W, 25.V.2008, R.P. Webster, coll. // Pond margin, in moist grass litter on mud (1 ♂, RWC). **Sunbury Co.**, Burton, SW of Sunpoke Lake, 45.7575°N, 66.5736°W, 17.IV.2005, R.P. Webster, coll. // Red maple swamp, in leaf litter near margin of slow [flowing] stream (1 ♂, NBM). **York Co.**, Charters Settlement, 45.8428°N, 66.7279°W, 9.V.2004, 19.V.2004, 23.VI.2004, 13.VIII.2004, R.P. Webster, coll. // Mixed forest, small sedge marsh, in moist grass litter (3 ♂, 2 ♀, NBM; 2 ♀, RWC); same locality but, 45.8395°N, 66.7391°W, 10.VI.2007, R.P. Webster, coll. // Mixed forest, u.v. light (1 ♀, NBM); 8.5 km W of Tracy, off Rt. 645, 45.6821°N, 66.7894°W, 6.V.2008, R.P. Webster, coll. // Wet alder swamp, in leaf litter & grass on hummock [near vernal pools] (1 ♂, 1 ♀, RWC); 9.2 km W of Tracy, off Rt. 645, 45.6837°N, 66.8809°W, 22.V.2008, R.P. Webster, coll. // *Carex* marsh adjacent to slow [flowing] stream, in *Carex* hummock (1 ♀, RWC).

######### Natural history.

In NB, *Philhygra larsoni* was found mostly in wetland habitats. Adults were found by splashing sun-exposed moss-covered rocks in a small river, treading *Carex* hummocks into water along pond margins, treading vegetation near a small pond in a seasonally flooded marsh, sifting moist grass litter near stream and pond margins, a red maple swamp, and sedge marshes, sifting grass and leaf litter on a hummock in a wet alder swamp, and sifting drift material on a lake margin. Nothing was previously known about the habitat associations of this species. In NF, *Philhygra larsoni* was collected from May to August without specific habitat data (Klimaszewski et al. 2011); in NB, adults were captured from mid-April to mid-August.

######### Distribution in Canada and Alaska.

**NB**, NF (Klimaszweski et al. 2011; Bousquet et al. 2013).

**Figures 226–233.**
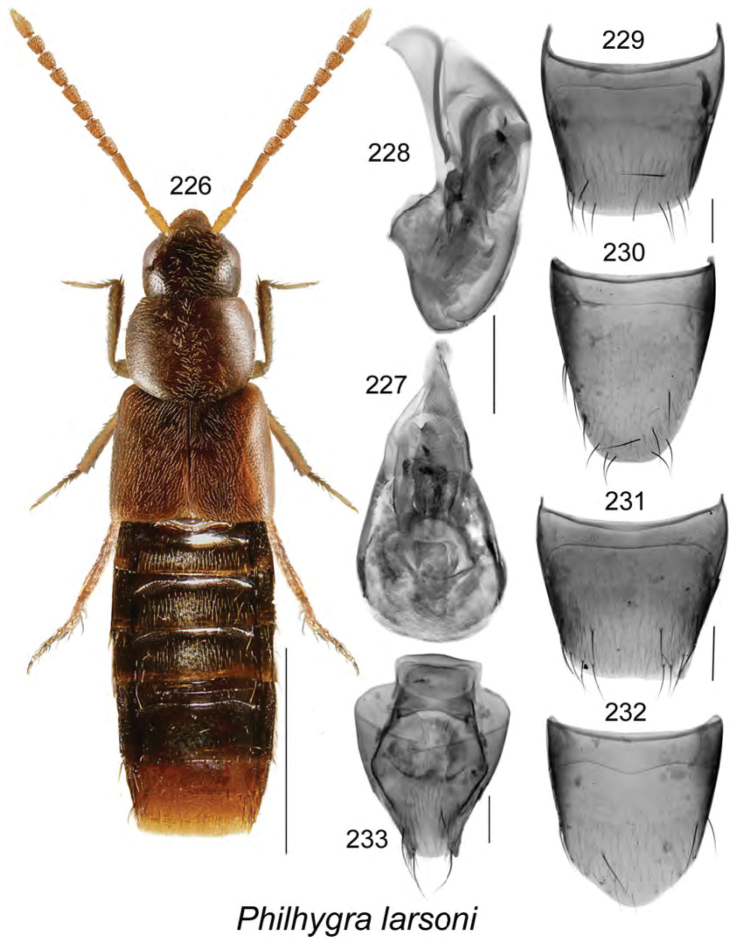
*Philhygra larsoni* Klimaszewski & Langor: **226** habitus in dorsal view **227** median lobe of aedeagus in dorsal view **228** median lobe of aedeagus in lateral view **229** male tergite VIII **230** male sternite VIII **231** female tergite VIII **232** female sternite VIII **233** female pygydium. Scale bar of habitus = 1 mm; remaining scale bars = 0.2 mm.

######## Philhygra proterminalis

Taxon classificationAnimaliaColeopteraStaphylinidae

(Bernhauer, 1907)

Figs 234–240

Philhygra proterminalis (For diagnosis, see Brunke et al. 2012)

######### Material examined.

**New Brunswick, Queens Co.**, W of Jemseg near “Trout Creek”, 45.8255°N, 66.1174°W, 1.VII.2008, R.P. Webster, coll. // Seasonally flooded marsh, treading vegetation near margin of pool (4 ♂, 2 ♀, RWC). **Sunbury Co.**, Burton, near Sunpoke Lake, 45.7658°N, 66.5546°W, 3.VII.2008, R.P. Webster, coll. // Red oak forest near flooded marsh, in leaf litter (1 ♂, 1 ♀, RWC); Gilbert Island, 45.8770°N, 66.2954°W, 8–21.VIII.2012, C. Alderson, C. Hughes, & V. Webster // Hardwood forest, Lindgren funnel trap 1 m high under *Tilia americana* (1 ♂, AFC). **York Co.**, Charters Settlement, 45.8340°N, 66.7450°W, 29.V.2008, R.P. Webster, coll. // Mature mixed forest, margin of vernal pond among moist leaves (1 ♂, 1 ♀, RWC); Douglas, Currie Mountain, 45.9844°N, 66.7592°W, 3–15.V.2013, C. Alderson & V. Webster // Mixed forest with *Quercus rubra*, Lindgren funnel trap 1 m high under *Quercus rubra* (1 ♂, RWC).

######### Natural history.

*Philhygra proterminalis* was found in various wetland habitats in NB. Adults were collected by treading vegetation near a vernal pool margin in a seasonally flooded marsh, sifting leaf litter in a red oak forest near a flooded seasonally flooded marsh, and by sifting moist leaves along a vernal pond margin in a mixed forest. Two individuals were captured in Lindgren funnel traps in a hardwood and mixed forest. Brunke et al. (2012) reported on specimens from a Lindgren funnel trap and from a madicolous spring in ON. Otherwise, nothing was previously known about the habitat associations of this species. Adults were collected during May, June, July, and August in NB and ON.

######### Distribution in Canada and Alaska.

ON, **NB** (Brunke et al. 2012; Bousquet et al. 2013). Brunke et al. (2012) reported this species for the first time for Canada from ON.

**Figures 234–240.**
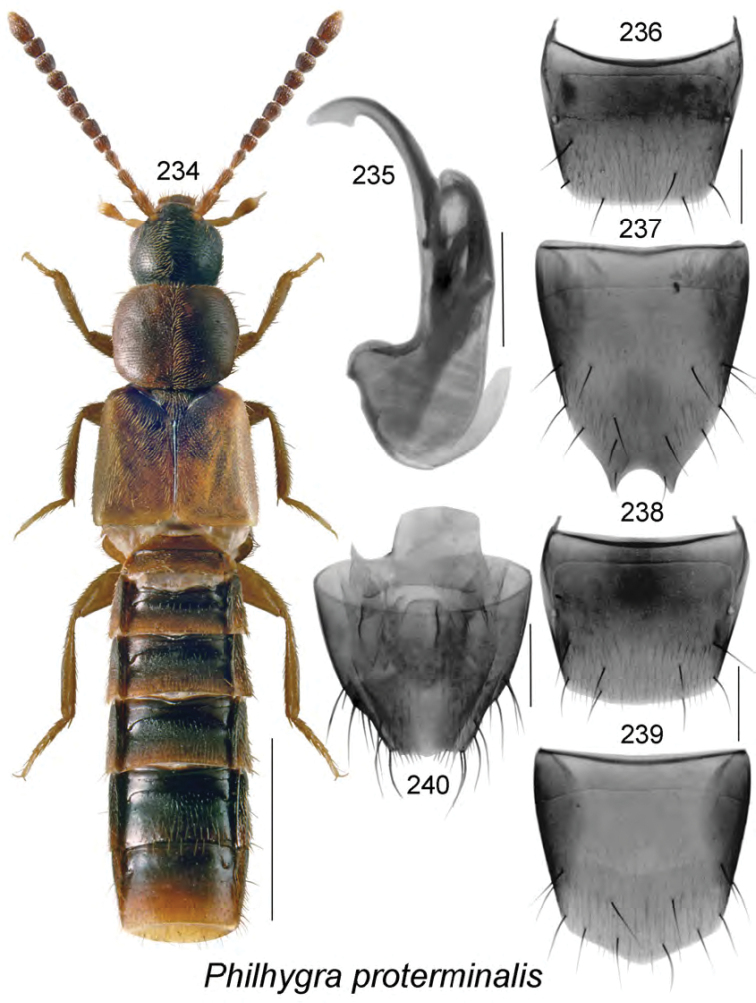
*Philhygra proterminalis* (Bernhauer): **234** habitus in dorsal view **235** median lobe of aedeagus in lateral view **236** male tergite VIII **237** male sternite VIII **238** female tergite VIII **239** female sternite VIII **240** female pygydium. Scale bar of habitus = 1 mm; remaining scale bars = 0.2 mm.

######## Philhygra pseudolarsoni

Taxon classificationAnimaliaColeopteraStaphylinidae

Klimaszewski & Godin, 2012

Figs 241–247

Philhygra pseudolarsoni (For diagnosis, see Klimaszewski et al. 2012)

######### Material examined.

**New Brunswick, Northumberland Co.**, Goodfellow Brook P.N.A., 46.8943°N, 65.3796°W, 23.V.2007, R.P. Webster, coll. // Old-growth eastern white cedar swamp, in litter, grasses & moss on hummocks near water (1 ♂, RWC). **Restigouche Co.**, Summit Lake, 47.7825°N, 68.3199°W, 7.VI.2011, R.P. Webster // Lake margin, *Carex* marsh, treading *Carex* hummocks and emergent vegetation (1 ♂, RWC); Wild Goose Lake, 420 m elev., 47.8540°N, 68.3219°W, 7.VI.2011, 20.VI.2011, R.P. Webster // Lake margin with emergent *Carex* and grasses, treading *Carex* and grasses (2 ♂, RWC). **Saint John Co.**, ca. 2 km NE of Maces Bay, 45.1161°N, 66.4560°W, 8.V.2006, R.P. Webster, coll. // Eastern white cedar swamp, in sphagnum and litter near brook (1 ♀, RWC). **York Co.**, Charters Settlement, 45.8395°N, 66.7391°W, 17.V.2010, R.P. Webster // Mixed forest opening, collected with net during evening flight between 16:30 and 18:00 h (1 ♂, RWC).

######### Natural history.

Most specimens of *Philhygra pseudolarsoni* from NB were found in wetland habitats. Adults were sifted from litter, grasses, and moss on hummocks near water and sifting sphagnum and litter near a brook in eastern white cedar swamps, and treading *Carex* hummocks and emergent vegetation in a *Carex* marsh along lake margins. One individual was collected with a net between 16:30 and 18:00 h in a mixed forest opening during a warm evening. The type and paratypes from the YT were sifted from soil litter from deciduous and mixed forests (Klimaszewski et al. 2012), otherwise little was previously known about the habitat associations of this species. Adults were collected during May and June.

######### Distribution in Canada and Alaska.

YT, **NB** (Klimaszewski et al. 2012; Bousquet et al. 2013). *Philhygra pseudolarsoni* was described from the YT (Klimaszewski et al. 2012). The data presented here suggest that this species has a transcontinental distribution in Canada.

**Figures 241–247.**
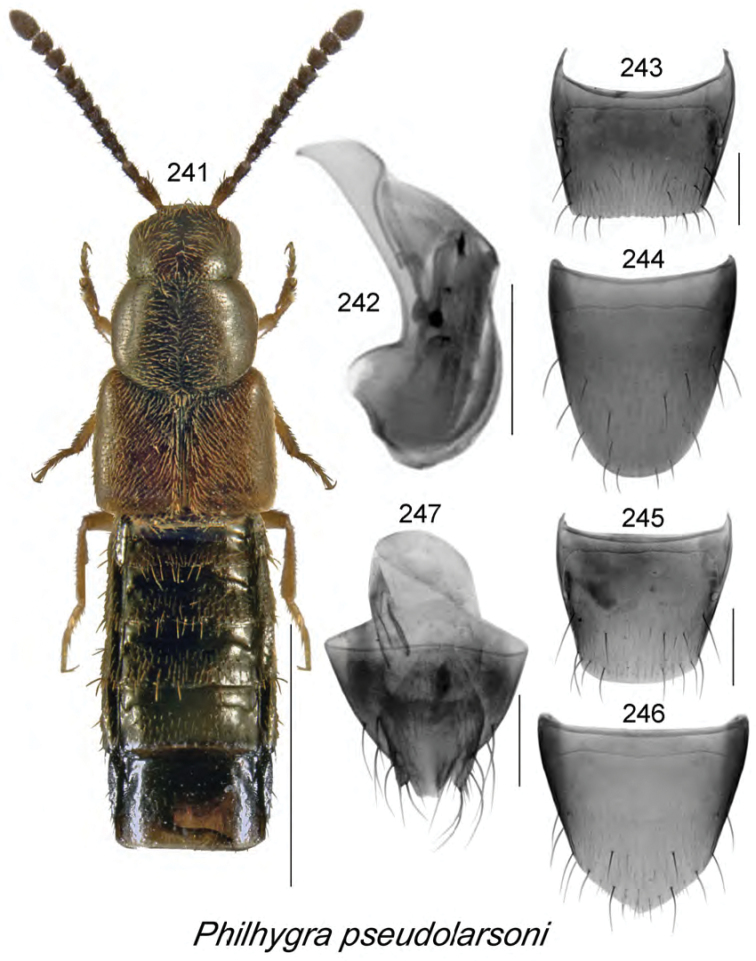
*Philhygra pseudolarsoni* Klimaszewski & Godin: **241** habitus in dorsal view **242** median lobe of aedeagus in lateral view **243** male tergite VIII **244** male sternite VIII **245** female tergite VIII **246** female sternite VIII **247** female pygydium. Scale bar of habitus = 1 mm; remaining scale bars = 0.2 mm.

######## Philhygra terrestris

Taxon classificationAnimaliaColeopteraStaphylinidae

Klimaszewski & Godin, 2012

Figs 248–254

Philhygra terrestris (For diagnosis, see Klimaszewski et al. 2012)

######### Material examined.

**New Brunswick, Madawaska Co.**, Third Lake, 47.7786°N, 68.3783°W, 21.VI.2010, R.P. Webster // Partially shaded brook, gravel/clay margin under alders (1 ♂, RWC); Jalbert Brook, 262 m elev., 47.6470°N, 68.3026°W, 23.VI.2010, R.P. Webster // Old-growth mixed forest, shaded brook, on clay/fine sand bar, collected by splashing (2 ♂, 2 ♀, RWC). **Restigouche Co.**, Jacquet River Gorge P.N.A., 47.8204°N, 66.0833°W, 14.VI.2009, R.P. Webster, coll. // River margin, splashing drift material (mostly small sticks and conifer bud debris) (1 ♂, RWC); Kedgwick Forks, 47.9085°N, 67.9057°W, 22.VI.2010, R.P. Webster // River margin, on clay/sand under alders (3 ♂, 1 ♀, RWC).

######### Natural history.

Most adults of *Philhygra terrestris* from NB were collected from shaded sites along brook and river margins. Specimens were found among gravel and clay under alders, by splashing clay and fine sand on sand bars along shaded brooks, and splashing drift material consisting of small sticks and conifer bud debris along a river margin. The type specimen from the YT was sifted from litter in a mixed forest during late May (Klimaszewski et al. 2012). Adults from NB were collected during June.

######### Distribution in Canada and Alaska.

YT, SK, **NB** (Bousquet et al. 2013, Klimaszewski et al. 2015a). *Philhygra terrestris* was described from the YT (Klimaszewski et al. 2012). The data presented here indicate that this species has a transcontinental distribution in Canada.

**Figures 248–254.**
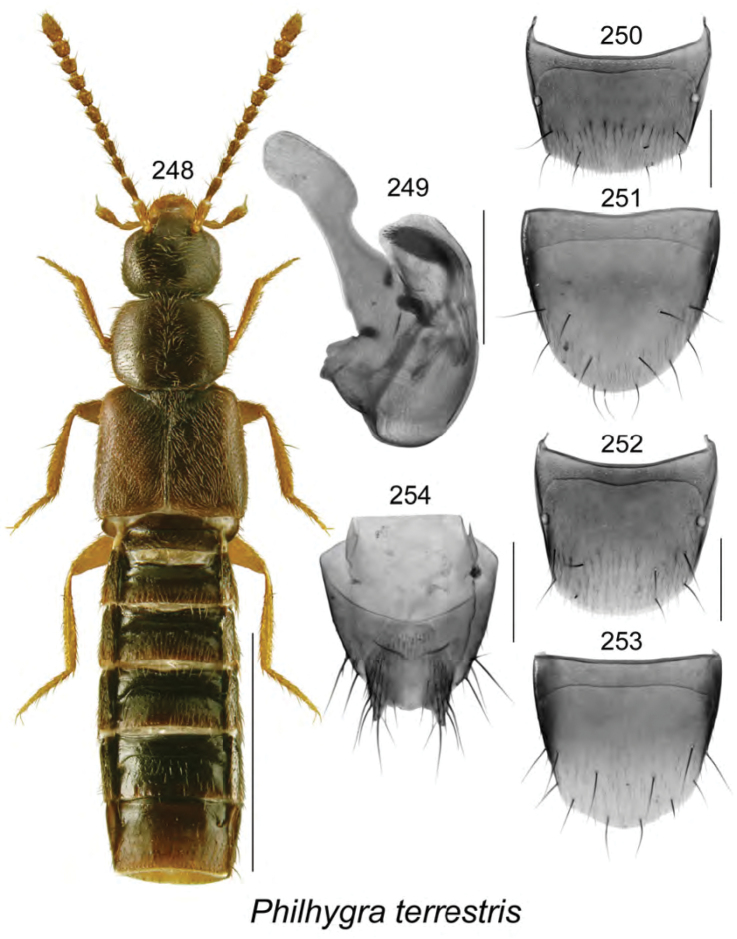
*Philhygra terrestris* Klimaszewski & Godin: **248** habitus in dorsal view **249** median lobe of aedeagus in lateral view **250** male tergite VIII **251** male sternite VIII **252** female tergite VIII **253** female sternite VIII **254** female pygydium. Scale bar of habitus = 1 mm; remaining scale bars = 0.2 mm.

######## Schistoglossa (Schistoglossa) pelletieri

Taxon classificationAnimaliaColeopteraStaphylinidae

Klimaszewski & Webster sp. n.

http://zoobank.org/26BE6B94-6CF0-494D-8F8A-53FC4C271F00

Figs 255–262

######### Holotype (male).

**Canada, New Brunswick, Albert Co.**, Caledonia Gorge P.N.A., 45.7930°N, 64.7764°W, 1.VII.2011, R.P. Webster, coll. // small rocky clear-cold river (Caledonia Creek), sifting drift material, tree bud material, in eddy area (LFC). **Paratypes: Canada, New Brunswick**, same data as for holotype (2 ♂, 1 ♀, RWC). **Restigouche Co.**, Jacquet River Gorge P.N.A., 47.8204°N, 66.0833°W, 14.VI.2009, R.P. Webster // Jacquet River, river margin among cobblestones (1 ♀, LFC; 1 ♀, RWC).

######### Etymology.

This species is named for our colleague Georges Pelletier (LFC) who participated in many of our entomology projects.

######### Description.

**Figures 255–262.**
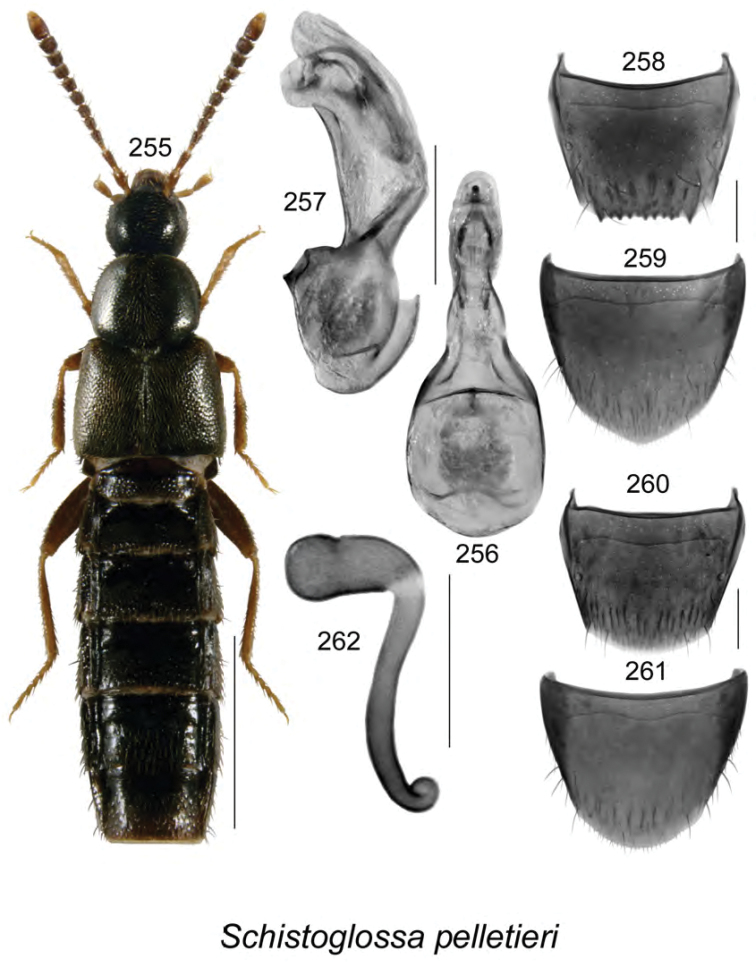
Schistoglossa (Schistoglossa) pelletieri Klimaszewski & Webster, sp. n.: **255** habitus in dorsal view **256** median lobe of aedeagus in dorsal view **257** median lobe of aedeagus in lateral view **258** male tergite VIII **259** male sternite VIII **260** female tergite VIII **261** female sternite VIII **262** spermatheca. Scale bar of habitus = 1 mm; remaining scale bars = 0.2 mm.

Body length 3.3–3.5 mm, narrowly oval, uniformly dark piceous with tibiae, tarsi, and base of antennae and mouthparts reddish brown (Fig. 255); integument glossy, pubescence short, except slightly longer on head and abdomen, yellowish brown in artificial light, sparse; head small, distinctly narrower than pronotum and elytra, approximately round with protruding apical part, feebly carinate basally, tempora as long as approximately three times maximal diameter of eye as seen from above; mandibles with apex split; antennae slim with articles V–X elongate or subquadrate to slightly transverse; pronotum slightly transverse, distinctly narrower than elytra, broadly arcuate laterally and posteriorly, broadest near base, strongly converging apically, pubescence directed posteriad on midline of disk and obliquely laterad elsewhere; elytra moderately transverse, subparallel, hind margin truncate, pubescence directed slightly obliquely posteriad; abdomen arcuate laterally, three basal tergites strongly impressed basally. **Male.** Median lobe of aedeagus with large bulbus in dorsal view (Fig. 256), venter of tubus arcuate, and apex slightly produced ventrally in lateral view (Fig. 257), structures of internal sac as illustrated (Figs 256, 257); tergite VIII with apical margin truncate and crenulate, with two moderate lateral teeth (Fig. 258); sternite VIII broadly parabolic, obtusely angulate apically (Fig. 259). **Female.** Tergite VIII broadly arcuate apically (Fig. 260); sternite VIII broadly rounded apically (Fig. 261); spermatheca S-shaped, with capsule tubular, angularly connected to stem, which is sharply curled at base (Fig. 262).

######### Distribution.

Known only from NB, Canada.

######### Natural history.

Adults of *Schistoglossa pelletieri* were sifted from drift material (tree bud material) along the margin of a small clear-cold river in an eddy area and found among cobblestones along a fast-flowing river. Specimens were collected during June and July.

######### Comments.

This species is readily distinguishable from other members of the subgenus by its large (3.3–3.5 mm long) dark piceous body, small head, and distinctively shaped genitalia (Figs 256–262). For other species of the genus in Canada, see Klimaszewski et al. (2009a).

######## Seeversiella globicollis

Taxon classificationAnimaliaColeopteraStaphylinidae

(Bernhauer, 1907)

Figs 263–270

Seeversiella globicollis (For diagnosis, see Klimaszewski et al. 2011)

######### Material examined.

**New Brunswick, Restigouche Co.**, Dionne Brook P.N.A., 47.9030°N, 68.3503°W, 25.V.2011, R.P. Webster // Old-growth northern hardwood forest, in moose dung (1 ♀, RWC); Dionne Brook P.N.A., 47.9064°N, 68.3441°W, 15–27.VI.2011, M. Roy & V. Webster // Lindgren funnel trap, Old-growth white spruce and balsam fir forest (1 ♀, RWC).

######### Natural history.

One NB specimen was collected from moose dung in an old-growth northern hardwood forest; another was captured in a Lindgren funnel trap in an adjacent old-growth white spruce and balsam fir forest. In NF, adults were collected from pitfall traps in fir and riparian forests (Klimaszewski et al. 2011). Gusarov (2003) reports the species from leaf litter, often near water. Specimens from NB were captured during May and June.

######### Distribution in Canada and Alaska.

BC, AB, SK, ON, QC, **NB**, NS, NF (Gusarov 2003b; Majka and Klimaszewski 2008b; Klimaszewski et al. 2011; Bousquet et al. 2013, Klimaszewski et al. 2015a).

**Figures 263–270.**
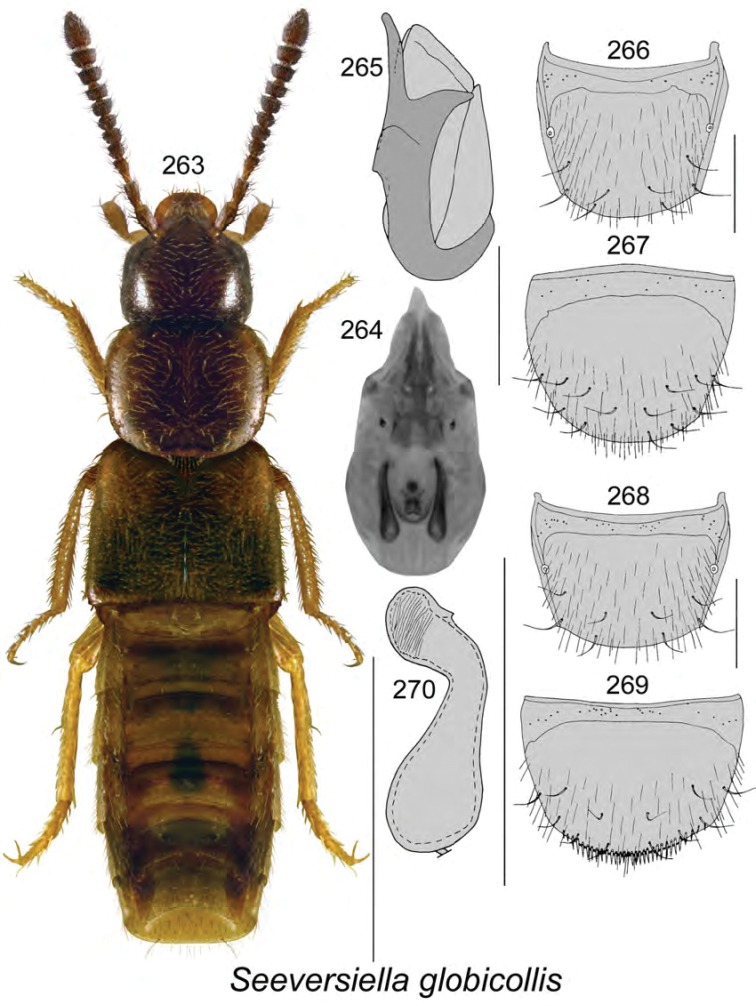
*Seeversiella globicollis* (Bernhauer): **263** habitus in dorsal view **264** median lobe of aedeagus in dorsal view **265** median lobe of aedeagus in lateral view **266** male tergite VIII **267** male sternite VIII **268** female tergite VIII **269** female sternite VIII **270** spermatheca. Scale bar of habitus = 1 mm; remaining scale bars = 0.2 mm.

######## Strigota ambigua

Taxon classificationAnimaliaColeopteraStaphylinidae

(Erichson, 1839)

Figs 271–278

Strigota ambigua (For diagnosis, see Klimaszewski et al. 2011, 2013a)

######### Material examined.

**New Brunswick, Queens Co.**, Canning, Grand Lake, Goat Island, 46.0110°N, 66.0133°W, 8.VIII.2007, R.P. Webster, coll. // Lake shore on cobblestone beach, under cobblestone on moist sand (1 ♀, RWC).

######### Natural history.

The single specimen from NB was found under a cobblestone on moist sand on a lake margin. Elsewhere, specimens have been found in various open habitats (Brunke et al. 2012 and references therein).

######### Distribution in Canada and Alaska.

YT, ON, QC, **NB**, NS, PE, LB, NF (Majka et al. 2008a; Klimaszewski et al. 2011; Brunke et al. 2012; Bousquet et al. 2013).

**Figures 271–278.**
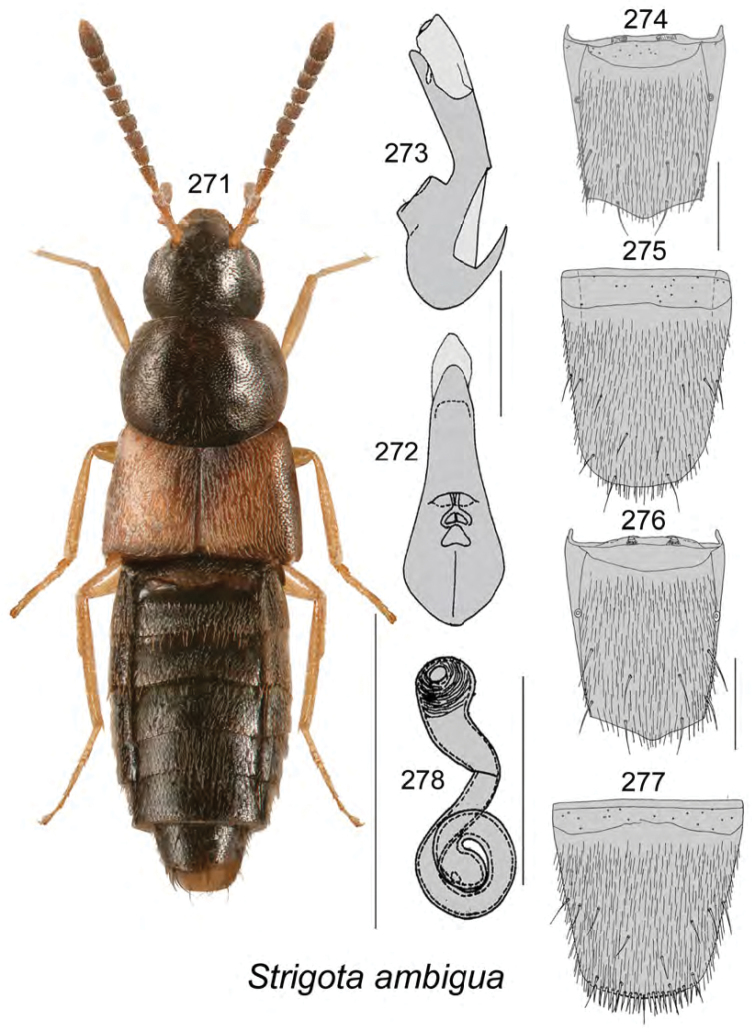
*Strigota ambigua* (Erichson): **271** habitus in dorsal view **272** median lobe of aedeagus in ventral view **273** median lobe of aedeagus in lateral view **274** male tergite VIII **275** male sternite VIII **276** female tergite VIII **277** female sternite VIII **278** spermatheca. Scale bar of habitus = 1 mm; remaining scale bars = 0.2 mm.

######## Strigota obscurata

Taxon classificationAnimaliaColeopteraStaphylinidae

Klimaszewski & Brunke, 2012

Figs 279–286

Strigota obscurata (For diagnosis and illustrations, see Brunke et al. 2012)

######### Material examined.

**New Brunswick, Northumberland Co.**, ca. 2.5 km W of Sevogle, 47.0876°N, 65.8613°W, 26.VI-6.VII.2013, C. Alderson & V. Webster // Old jack pine forest, in Lindgren funnel trap (1 ♀, RWC). **York Co.**, Fredericton, at Saint John River, 45.9588°N, 66.6254°W, 7.VI.2005, R.P. Webster, coll. // River margin, in flood debris (1 ♀, RWC); Charters Settlement, 45.8395°N, 66.7391°W, 5.X.2005, R.P. Webster, coll. // Residential lawn, on soil at base of grass (1 ♀, RWC).

######### Natural history.

In NB, *Strigota obscurata* were found in flood debris on a river margin, on soil at the base of grass in a residential lawn, and captured in a Lindgren funnel trap in an old jack pine forest. Brunke et al. (2012) reported this as the most common species in southern ON soybean fields, often occurring in open habitats with *Strigota ambigua*.

######### Distribution in Canada and Alaska.

ON, **NB** (Bousquet et al. 2013). Although previously known only from ON at the time of description, Brunke et al. (2012) expected that the species would occur widely in northeastern North America. It was cited from QC in Bousquet et al. (2013), based on information submitted by G. Pelletier (LFC, pers. comm.), who indicated that it was verified by Klimaszewski; Klimaszewski (pers. comm.) was unable to find specimens at LFC from QC, and it is therefore provisionally removed from QC.

**Figures 279–286.**
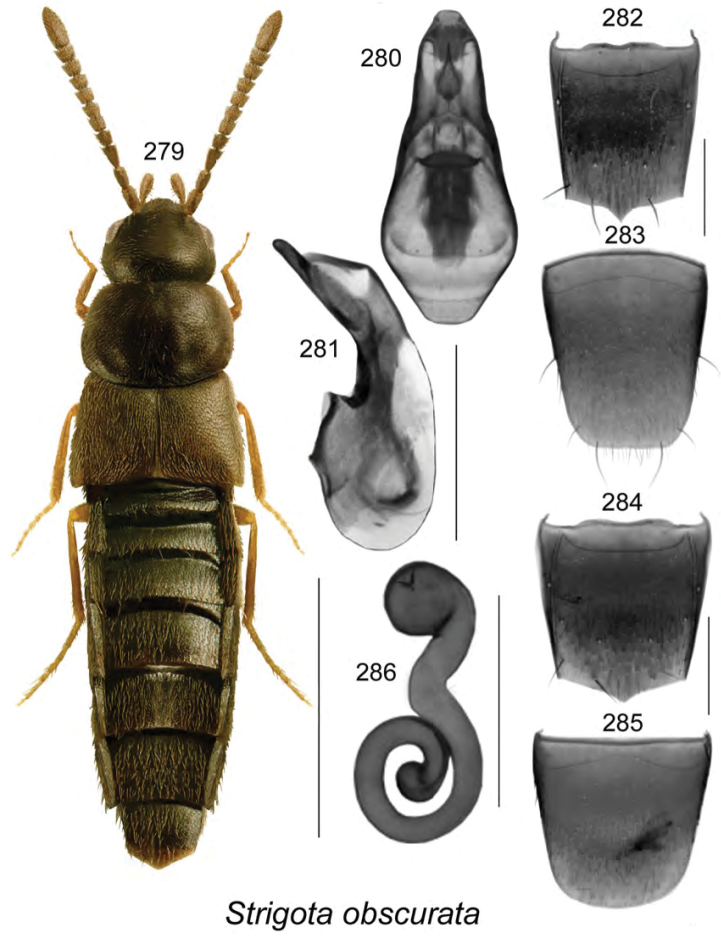
*Strigota obscurata* Klimaszewski & Brunke: **279** habitus in dorsal view **280** median lobe of aedeagus in dorsal view **281** median lobe of aedeagus in lateral view **282** male tergite VIII **283** male sternite VIII **284** female tergite VIII **285** female sternite VIII **286** spermatheca. Scale bar of habitus = 1 mm; remaining scale bars = 0.2 mm.

######## Trichiusa hirsuta

Taxon classificationAnimaliaColeopteraStaphylinidae

Casey, 1906

Figs 287–294

Trichiusa hirsuta (For diagnosis see Brunke et al. 2012)

######### Material examined.

**New Brunswick, Carleton Co.**, Jackson Falls, “Bell Forest”, 46.2200°N, 67.7231°W, 2–12.VI.2008, 12–19.VI.2008, 5–12.VII.2008, R.P. Webster, coll. // Rich Appalachian hardwood forest with some conifers, Lindgren funnel traps (2 ♂, 2 ♀, LFC); same data but 1–8.VI.2009, 8–16.VI.2009, 21–28.VI.2009, R. Webster & M.-A. Giguère, coll. // Rich Appalachian hardwood forest with some conifers, Lindgren funnel traps (3 ♂, 2 ♀, RWC). **Charlotte Co.**, 5.2 km NW of Pomeroy Ridge, 45.3087°N, 67.4362°W, 16.VI.2008, R.P. Webster, coll. // Red maple swamp, in sphagnum with grasses near vernal pond (1 ♂, LFC; 1 ♀, RWC). **Northumberland Co.**, ca, 2.5 km W of Sevogle, 47.0876°N, 65.8613°W, 28.V-11.VI.2013, C. Alderson & V. Webster // Old *Pinus banksiana* stand, Lindgren funnel trap (3 sex undetermined, AFC); ca. 1.5 km NW of Sevogle, 47.0939°N, 65.8387°W, 28.V-11.VI.2013, C. Alderson & V. Webster // *Populus tremuloides* stand with a few conifers, Lindgren funnel trap 1 m high under *Populus tremuloides* (1 sex undetermined, AFC). **Saint John Co.**, Chance Harbour off Rt. 790, 45.1355°N, 66.3672°W, 12.V.2008, R.P. Webster, coll. // Calcareous fen, in sphagnum & litter in depressions with *Carex* (1 ♂, RWC). **Sunbury Co.**, Acadia Research Forest, 45.9866°N, 66.3841°W, 19–25.V.2009, 25.V-2.VI.2009, 2–16.VI.2009, R. Webster & M.-A. Giguère, coll. // Red spruce forest with red maple and balsam fir, Lindgren funnel traps (2 ♂, AFC; 1 ♀, RWC). **York Co.**, 15 km W of Tracy, off Rt. 645, 45.6848°N, 66.8821°W, 1–8.VI.2009, 28.VI-7.VII.2009, R. Webster & M.-A. Giguère, coll. // Red pine forest, Lindgren funnel traps (1 ♂, 1 ♀, RWC).

######### Natural history.

Most adults from NB were captured in Lindgren funnel traps in the following forest types: rich Appalachian hardwood forest, old jack pine stand, trembling aspen (*Populus tremuloides* Michx.) stand, red spruce forest with red maple and balsam fir, and a red pine (*Pinus resinosa* Ait.) forest. Specimens with microhabitat data were sifted from sphagnum and grasses near a vernal pond in a red maple swamp, and sphagnum and litter in depressions with *Carex* in a calcareous fen. In ON, Brunke et al. (2012) reported *Trichiusa hirsuta* from upland forest or semi-forest habitats on sandy soil. Adults were collected during May and June in both ON (Brunke et al. 2012) and NB.

######### Distribution in Canada and Alaska.

ON, **NB** (Brunke et al. 2012; Bousquet et al. 2013). Brunke et al. (2012) reported *Trichiusa hirsuta* for the first time for Canada from ON. This species is widespread in NB.

**Figures 287–294.**
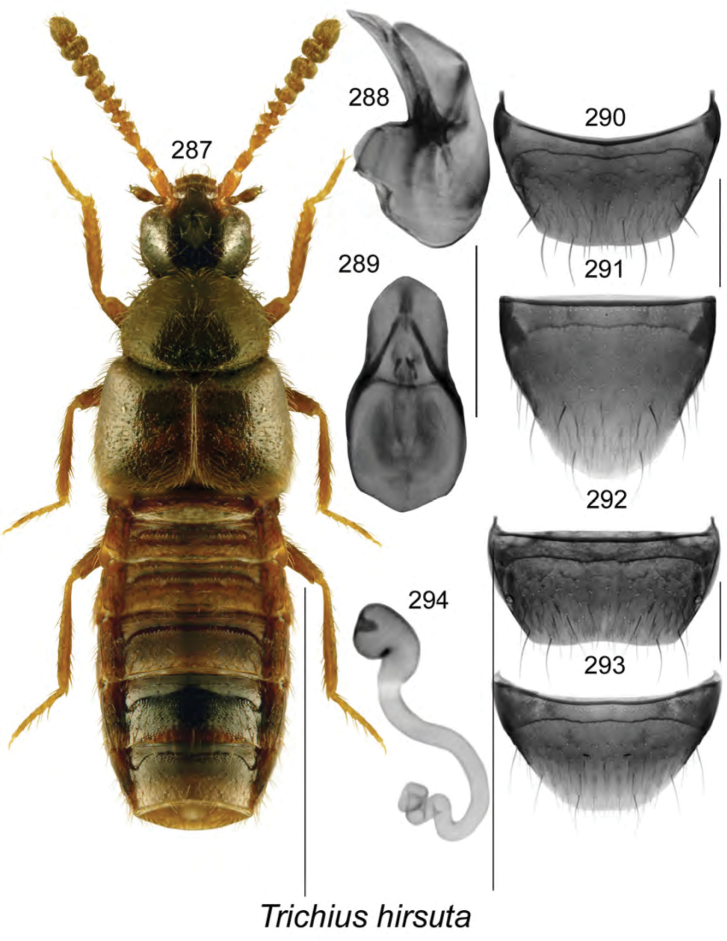
*Trichiusa hirsuta* Casey: **287** habitus in dorsal view **288** median lobe of aedeagus in lateral view **289** median lobe of aedeagus in dorsal view **290** male tergite VIII **291** male sternite VIII **292** female tergite VIII **293** female sternite VIII **294** spermatheca. Scale bar of habitus = 1 mm; remaining scale bars = 0.2 mm.

######## Trichiusa pilosa

Taxon classificationAnimaliaColeopteraStaphylinidae

Casey, 1894

Figs 295–302

Trichiusa pilosa (For diagnosis, see Klimaszewski et al. 2015a)

######### Material examined.

**New Brunswick, Queens Co.**, Grand Lake at Youngs Cove, 45.96358°N, 65.99793°W, 4.VIII.2005, R.P. Webster, coll. // Lake margin, cobblestone beach, under cobblestones (2 ♀, RWC). **Sunbury Co.**, Gilbert Island, 45.8770°N, 66.2954°W, 12–29.VI.2012, C. Alderson, C. Hughes & V. Webster // Hardwood forest, Lindgren funnel trap in canopy of *Populus tremuloides* (1 ♂, RWC).

######### Natural history.

Two individuals were collected from under cobblestones along a lakeshore in August, another was captured in a Lindgren trap in the canopy of a trembling aspen in a hardwood forest in June. In Alberta, one female was collected with a window trap and in British Columbia, specimens were found in bison dung (Klimaszewski et al. 2015a).

######### Distribution in Canada and Alaska.

BC, AB, ON, **NB**, NS (Klimaszewski et al. 2015a).

######### Comments.

Klimaszewski et al. (2015a) synonomized this species with *Trichiusa atra* Casey, *Trichiusa monticola* Casey, *Trichiusa parviceps* Casey, and *Trichiusa postica* Casey. *Trichiusa pilosa* was previously reported from NS and ON (as *Trichiusa postica*) by Majka and Klimaszewski (2010) and Casey (1906) [type loc.], respectively.

**Figures 295–302.**
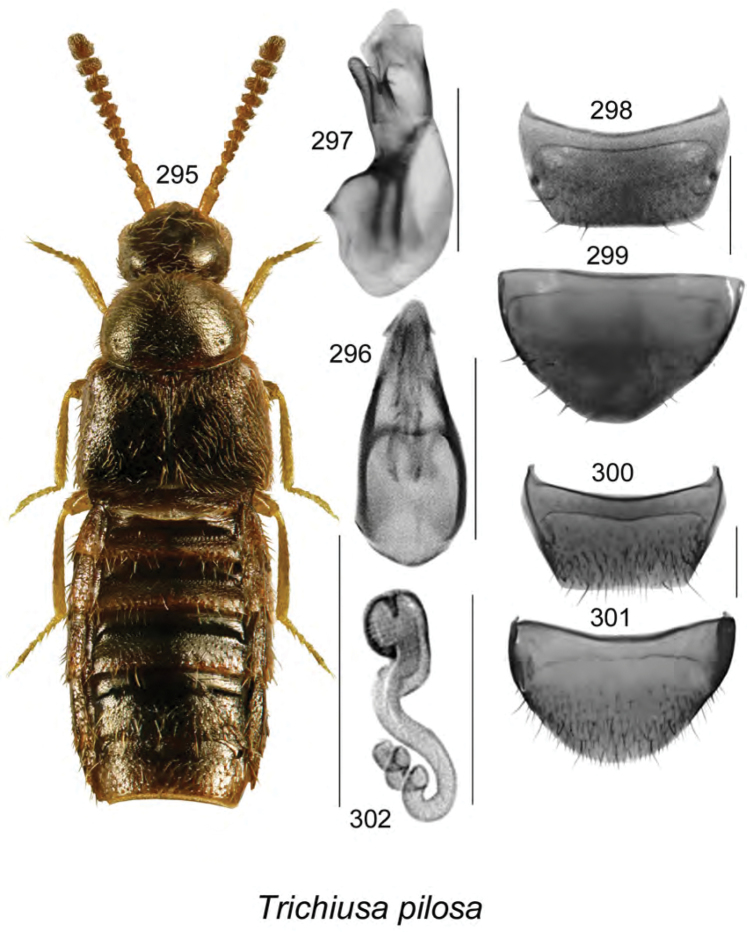
*Trichiusa pilosa* Casey: **295** habitus in dorsal view **296** median lobe of aedeagus in dorsal view **297** median lobe of aedeagus in lateral view **298** male tergite VIII **299** male sternite VIII **300** female tergite VIII **301** female sternite VIII **302** spermatheca. Scale bar of habitus = 1 mm; remaining scale bars = 0.2 mm.

######## Trichiusa robustula

Taxon classificationAnimaliaColeopteraStaphylinidae

Casey, 1894

Figs 303–309

Trichiusa robustula (For diagnosis, see Brunke et al. 2012)

######### Material examined.

**New Brunswick, York Co.**, Fredericton, at Saint John River, 45.9588°N, 66.6254°W, 4.VII.2004, R.P. Webster // Margin of river, in drift material, mostly maple seeds (1 sex undetermined, LFC); Charters Settlement, 45.8395°N, 66.7391°W, 6.IX.2005, 16.IX.2005, 25.IX.2005, 27.IX.2005, 28.IX.2005, 23.IV.2008, 27.IV.2008, R.P. Webster, coll. // Mixed forest, in compost (decaying vegetable matter) (4 ♂, 1 ♀, 3 sex undetermined, LFC; 3 ♂, 3 ♀, 3 sex undetermined, RWC).

######### Natural history.

Most individuals of *Trichiusa robustula* from NB were sifted from compost near a mixed forest during April and September. The species was very common at this site. One individual from a river margin was sifted during July from drift material consisting mostly of maple seeds. Brunke et al. (2012) reported this species from debris along lakeshores and from a grass pile and leaves near a lakeshore in ON.

######### Distribution in Canada.

ON, **NB** (Bousquet et al. 2013). Brunke et al. (2012) reported *Trichiusa robustula* for the first time for Canada from ON.

**Figures 303–309.**
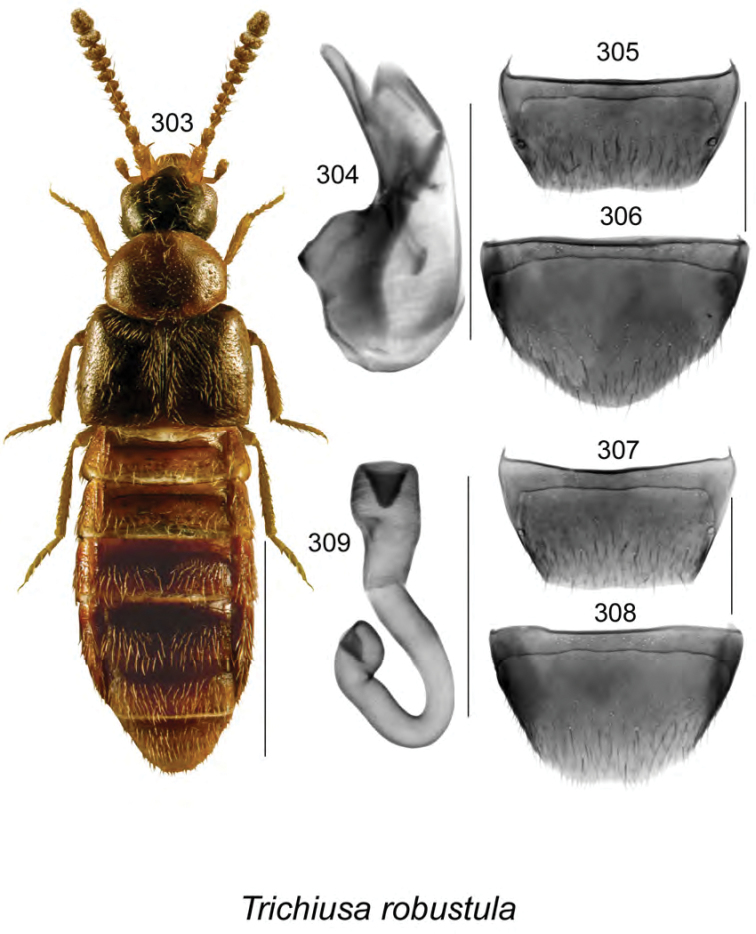
*Trichiusa robustula* Casey: **303** habitus in dorsal view **304** median lobe of aedeagus in lateral view **305** male tergite VIII **306** male sternite VIII **307** female tergite VIII **308** female sternite VIII **309** spermatheca. Scale bar of habitus = 1 mm; remaining scale bars = 0.2 mm.

####### Subtribe Thamiaraeina Fenyes, 1921

######## Thamiaraea claydeni

Taxon classificationAnimaliaColeopteraStaphylinidae

Klimaszewski & Webster sp. n.

http://zoobank.org/F5E42DB7-110A-4729-8633-27609765365C

Figs 310–316

######### Holotype (male).

**Canada, New Brunswick, Queens Co.**, Jemseg, 45.8412°N, 66.1195°W, 21.VIII-7.IX.2012, C. Hughes & K. Van Rooyen // hardwood woodland near seasonally flooded marsh, Lindgren funnel trap in canopy of *Quercus macrocarpa* (LFC). **Paratypes: Canada, New Brunswick, Queens Co**., Jemseg, 45.8412°N, 66.1195°W, 2–14.V.2012, C. Hughes & R. Webster // hardwood woodland near seasonally flooded marsh, Lindgren funnel trap 1 m under *Quercus macrocarpa* (1 ♂, RWC); Grand Lake meadows P.N.A., 45.8227°N, 66.1209°W, 31.V-15.VI.2010, R. Webster & C. MacKay, coll. // Old silver maple forest with green ash and seasonally flooded marsh, Lindgren funnel trap (1 ♀, LFC); same data but 15–31.V.2010 (1 ♂, 1 ♀, RWC); same data but 29.VI-12.VII.2010, R. Webster, C. MacKay, M. Laity & R. Johns, coll. (1 ♂, RWC). **Sunbury Co.**, Burton, Sunpoke Lake, 45.7665°N, 66.5545°W, 15.V.2004, R.P. Webster, coll. // Old maple forest, in leaf litter (1 ♂, RWC). **York Co.**, Fredericton, at Saint John River, 45.9588°N, 66.6254°W, 22.VIII.2006, R.P. Webster, coll. // River margin, in decaying (moist) grass (1 ♀, RWC); 8.5 km W of Tracy, off Rt. 645, 45.6821°N, 66.7894°W, 6.V.2008, R.P. Webster, coll. // wet alder swamp, in leaf litter & grass on hummocks (1 ♂, RWC).

######### Etymology.

Named for Dr. Stephen Clayden, Curator and Head, Botany and Mycology Section of the New Brunswick Museum, whose collaboration in a joint project studying Coleoptera and lichens in old-growth eastern white cedar forests in NB resulted in the discovery of a number of new species.

######### Description.

**Figures 310–316.**
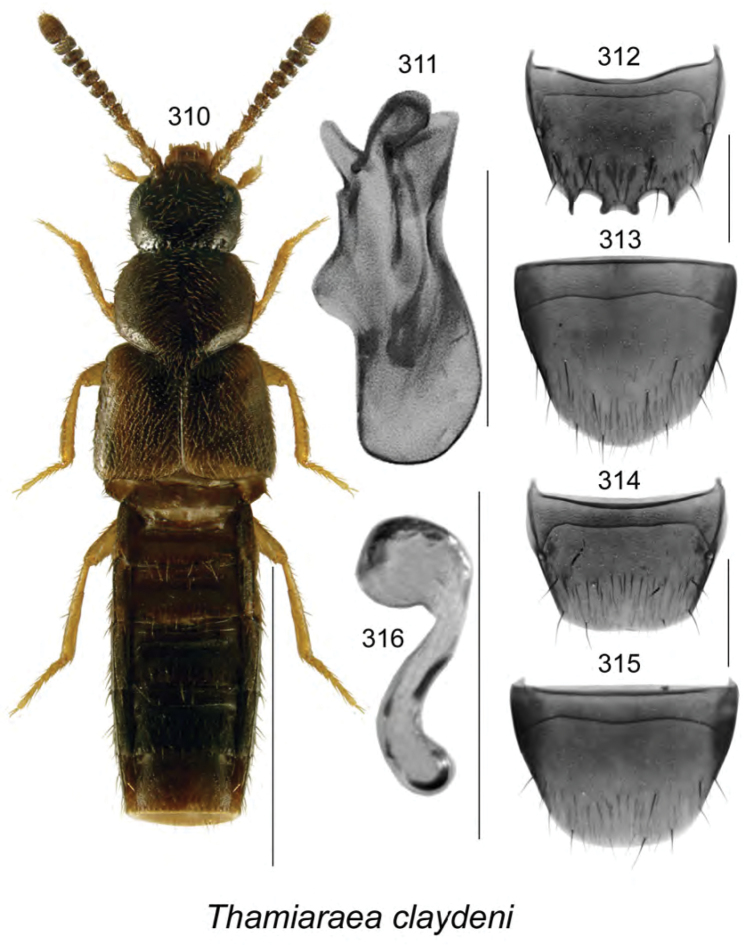
*Thamiaraea claydeni* Klimaszewski & Webster, sp. n.: **310** habitus in dorsal view **311** median lobe of aedeagus in lateral view **312** male tergite VIII **313** male sternite VIII **314** female tergite VIII **315** female sternite VIII **316** spermatheca. Scale bar of habitus = 1 mm; remaining scale bars = 0.2 mm.

Body length 2.5–2.7 mm, narrowly subparallel, uniformly dark piceous brown except posterior part of elytra near suture and basal tergal impressions slightly paler, legs, maxillary palpi and bases of antennae light yellowish brown (Fig. 310); integument glossy with meshed microsculpture, pubescence short, dense on pronotum and elytra and sparse on head and abdomen; head narrower than pronotum and elytra, approximately round, tempora about as long as eye seen from above; antennae with articles V–X slightly to strongly transverse; pronotum transverse, margined laterally and basally, narrower than elytra, obtusely angular posterolaterally, broadest at middle of its length, pubescence directed lateroposteriad forming arcuate lines; elytra moderately short, moderately transverse, subparallel, hind margin straight laterally, inwardly arcuate toward suture, pubescence directed obliquely posteriad; abdomen parallel-sided, three basal tergites strongly impressed basally. **Male.** Median lobe of aedeagus with large bulbus and short tubus, venter of tubus with tooth medially, apex narrow, produced ventrally in lateral view, sclerites of internal sac not pronounced except for strong apical folds (Fig. 311); apical margin of tergite VIII emarginate, with two spine-like lateral teeth and two diverging, more rounded ones at middle (Fig. 312); sternite VIII rounded apically (Fig. 313). **Female.** Tergite VIII truncate apically (Fig. 314); sternite VIII broadly rounded apically (Fig. 315); spermatheca S-shaped, with broad, spherical capsule, and short, broad, sinuate stem (Fig. 316).

######### Distribution.

Known only from NB, Canada.

######### Natural history.

This species occurs in very similar habitats to *Thamiaraea corverae*; in silver maple and maple forests near seasonally flooded marshes, a river margin, and in a wet alder swamp. Adults were found in moist leaf litter and moist decaying grass along a river margin. Other specimens were captured in Lindgren funnel traps. Adults were collected from May to September.

######## Thamiaraea corverae

Taxon classificationAnimaliaColeopteraStaphylinidae

Klimaszewski & Webster sp. n.

http://zoobank.org/958C873E-3A06-4FAA-AAB9-A9674EFFCBB6

Figs 317–323

######### Holotype (male).

**Canada, New Brunswick, Sunbury Co.**, Gilbert Island, 45.8770°N, 66.2954°W, 29.VI-11.VII.2012, C. Alderson & V. Webster, coll. // hardwood forest, Lindgren funnel trap 1 m high under *Tilia americana* (LFC). **Paratypes: Canada, New Brunswick, Charlotte Co.**, 5 km NW of Pomeroy Ridge, 45.3059°N, 67.4343°W, 5.VI.2008, R.P. Webster, coll. // red maple and eastern white cedar swamp, in moss and leaf litter near small vernal pools (1 ♀, LFC). **Queens Co.**, Grand Lake Meadows P.N.A., 45.8227°N, 66.1209°W, 4–19.V.2010, R. Webster & C. MacKay, coll. // Old silver maple forest with green ash and seasonally flooded marsh, Lindgren funnel traps (2 ♂, RWC); Jemseg, 45.8412°N, 66.1195°W, 14–28.V.2012, C. Alderson, C. Hughes & V. Webster // Hardwood woodland near seasonally flooded marsh, Lindgren funnel trap 1 m high under *Quercus rubra* (1 ♂, RWC). **York Co.**, Prince William, near Magaguadavic Lake, 45.7268°N, 66.1852°W, 1.V.2004, D. Sabine & R. Webster, coll. // Red spruce & hemlock forest, in moist litter under leather-leaf (1 ♂, 1 ♀, RWC); Fredericton, at Saint John River, 45.9588°N, 66.6254°W, 22.VIII.2006, R.P. Webster, coll. // River margin, in decaying grass (1 ♂, RWC).

######### Etymology.

The first author of the species, Jan Klimaszewski, would like to dedicate this species to his wife, Patricia Corvera Gandullia, for her love of nature and enthusiasm for entomology.

######### Description.

**Figures 317–323.**
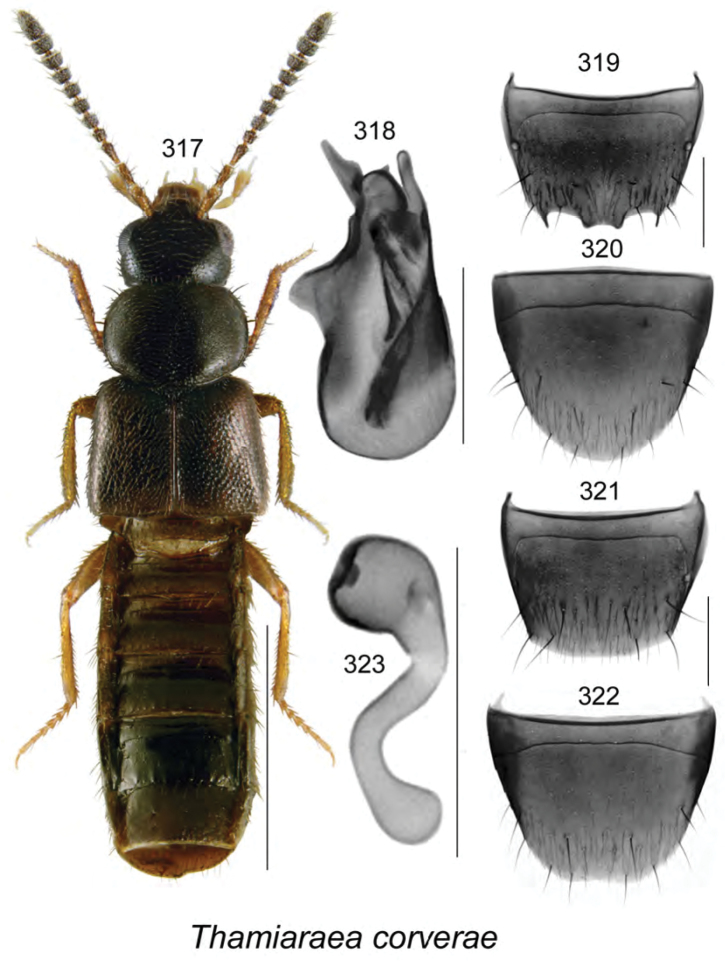
*Thamiaraea corverae* Klimaszewski & Webster, sp. n.: **317** habitus in dorsal view **318** median lobe of aedeagus in lateral view **319** male tergite VIII **320** male sternite VIII **321** female tergite VIII **322** female sternite VIII **323** spermatheca. Scale bar of habitus = 1 mm; remaining scale bars = 0.2 mm.

Body length 2.8–3.1 mm, narrowly subparallel, most of antennae, head, and posterior part of abdomen dark piceous brown, pronotum slightly paler, elytra yellowish light brown, legs, maxillary palpi, and bases of antennae yellowish (Fig. 317); integument glossy with meshed microsculpture, pubescence short, dense on pronotum and elytra and sparse on head and abdomen; head slightly narrower than pronotum, approximately round, tempora slightly shorter than eye seen from above; antennae with articles V–X slightly to strongly transverse; pronotum transverse, margined laterally, slightly narrower than elytra, broadly arcuate laterally, broadest at middle of its length, pubescence directed lateroposteriad forming arcuate lines; elytra short, moderately transverse, subparallel, hind margin approximately straight, pubescence directed obliquely posteriad; abdomen parallel-sided, three basal tergites strongly impressed basally. **Male.** Median lobe of aedeagus with bulbus large and tubus short, triangular in dorsal view, venter of tubus arcuate, and apex narrow, triangularly produced ventrally in lateral view, sclerites of internal sac not pronounced (Fig. 318); apical margin of tergite VIII emarginate, with two spine-like teeth near lateral margin, and two rounded ones forming median projection (Fig. 319); sternite VIII semicircularly rounded apically (Fig. 320). **Female.** Tergite VIII broadly arcuate apically (Fig. 321); sternite VIII broadly shallowly emarginate apically (Fig. 322); spermatheca S-shaped, with spherical capsule, and short, sinuate stem which broadens basally (Fig. 323).

######### Distribution.

Known only from NB, Canada.

######### Natural history.

This species was found in or near seasonally flooded silver maple forests and marshes, an eastern white cedar swamp, a river margin, and a wetland dominated by leather-leaf, *Chamaedaphne calyculata* (L.). Adults were found in moss and leaf litter, moist litter under leather-leaf, and decaying grass along a river margin. Other specimens were captured in Lindgren funnel traps. Adults were collected from May to August.

######### Comments.

*Thamiaraea corverae* may be easily separated from *Thamiaraea claydeni* by darker and broader body, less transverse antennal articles VII-X (Figs 310, 317), median teeth of male tergite VIII directed posteriad (Fig. 319) and not diverging laterad as in *Thamiaraea claydeni* (Fig. 312), and spermatheca with more sinuate stem (Fig. 323) than that of *Thamiaraea claydeni* (Fig. 316). From the remaining three Nearctic *Thamiaraea* species, the two species described here may be distinguished by the shape of the median lobe of aedeagus, shape of male tergite VIII and the shape of spermathecae. For illustrations of the other species, see Hoebeke 1988, 1994.

###### Tribe Falagriini Mulsant & Rey, 1873

####### Myrmecopora vaga

Taxon classificationAnimaliaColeopteraStaphylinidae

(LeConte, 1866)

Figs 324–330

######## Material examined.

**New Brunswick, Westmorland Co.**, Petit-Cap, 46.1836°N, 64.1468°W, 19.VI.2012, R.P. Webster & D. Sabine // Sandy barrier beach, sifting drift material (mostly dried/decaying sea wrack) (1 ♀, LFC; 1 ♂, 1 ♀, RWC); same data but 17.VI.2014, M.-A. Giguère (2 sex undetermined, RWC).

######## Natural history.

*Myrmecophora vaga* was sifted from drift material consisting mostly of dried and decaying sea wrack on a sandy barrier sea beach. Majka et al. (2008a) reported this species from a similar habitat (flotsam on small beach) from NS. A number of western Palaearctic species of *Myrmecopora* also live in beach drift on coastal sea beaches (Assing 1997).

######## Distribution in Canada and Alaska.

NS, **NB** (Bousquet et al. 2013). Majka et al. (2008a) reported this species for the first time for Canada from NS. The species has not been identified again in the Lake Superior region since the original description; although Ahn and Ashe (1995: 151) examined specimens in their phylogenetic study, they did not specify any locality data.

######## Comments.

*Myrmecopora vaga* bears superficial resemblance to European *Myrmecopora uvida* (Erichson) but has differently shaped median lobe of aedeagus with shorter and straight ventral part of tubus in lateral view which is longer and sinuate in *Myrmecopora uvida*, and by the shorter and broader stem of spermatheca. For illustrations of genitalia of *Myrmecopora uvida* see Assing 1997.

**Figures 324–330.**
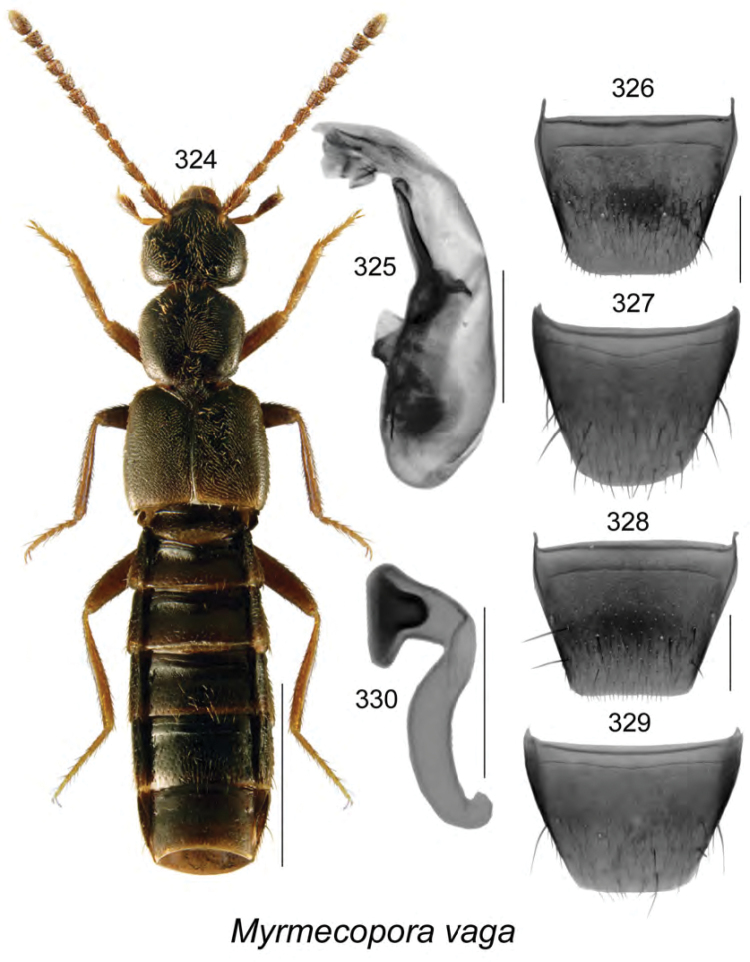
*Myrmecopora vaga* (LeConte): **324** habitus in dorsal view **325** median lobe of aedeagus in lateral view **326** male tergite VIII **327** male sternite VIII **328** female tergite VIII **329** female sternite VIII **330** spermatheca. Scale bar of habitus = 1 mm; remaining scale bars = 0.2 mm.

###### Tribe Homalotini Heer, 1839

####### Subtribe Bolitocharina C.G. Thomson, 1859

######## Pleurotobia bourdonae

Taxon classificationAnimaliaColeopteraStaphylinidae

Klimaszewski & Webster sp. n.

http://zoobank.org/44DF6F6C-9FDC-4F05-A082-5503A38A05FB

Figs 331–337

######### Holotype (male).

**Canada, New Brunswick, Carleton Co.**, Jackson Falls, “Bell Forest”, 46.2200°N, 67.7231°W, 18.VIII.2008, R.P. Webster, coll. // rich Appalachian hardwood forest in *Hapalopilus nidulans* on dead standing beech tree (LFC). **Paratypes: Canada, New Brunswick, Carleton Co.**, same data as holotype (1 ♀, LFC; 3 ♂, 2 ♀, RWC); same data except 20.IX.2008 (1 ♂, RWC). **York Co.**, Canterbury, near “Browns Mtn. Fen”, 45.8876°N, 67.6560°W, 3.VIII.2006, R.P. Webster, coll. // Hardwood forest, on *Pleurotus* sp. on sugar maple (1 ♂, LFC). **Quebec, Bellechasse Co.**, St. Raphael, 46.8078°N, 70.7344°W, 15.VII.2006, R.P. Webster, coll. // Mixed forest, on decaying fleshy polypore on dead standing poplar (1 ♀, RWC).

######### Etymology.

This species is named for Caroline Bourdon (LFC) who works with us on many projects and has produced many images.

######### Description.

**Figures 331–337.**
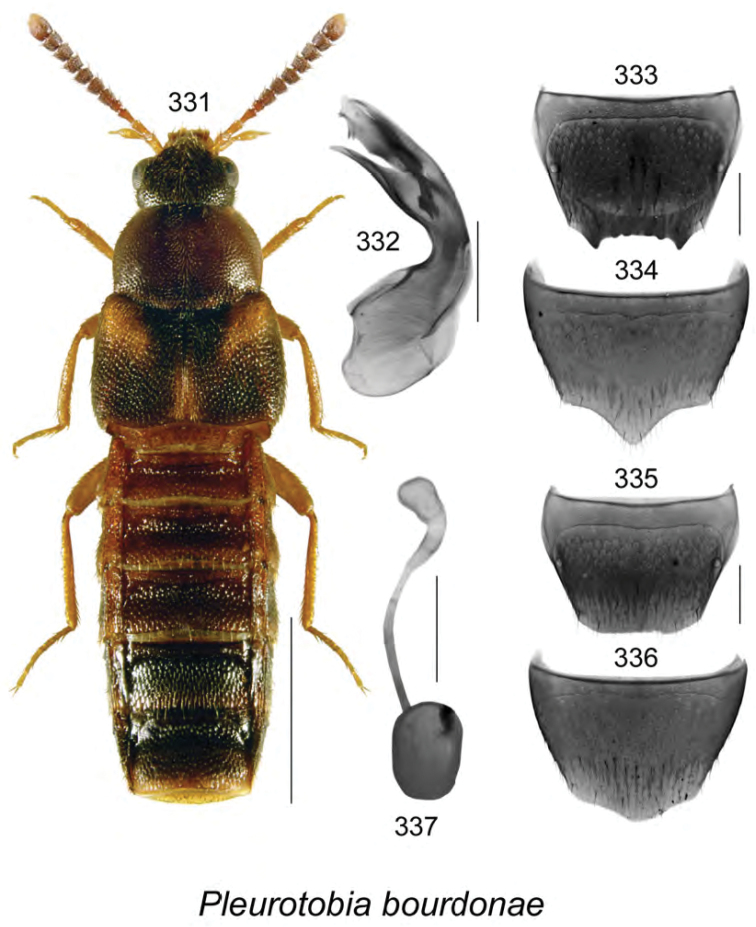
*Pleurotobia bourdonae* Klimaszewski & Webster, sp. n.: **331** habitus in dorsal view **332** median lobe of aedeagus in lateral view **333** male tergite VIII **334** male sternite VIII **335** female tergite VIII **336** female sternite VIII **337** spermatheca. Scale bar of habitus = 1 mm; remaining scale bars = 0.2 mm.

Body length 3.8–4.0 mm, narrowly oval, robust, head, pronotum, most of elytra and posterior part of abdomen dark brown, elytra with a yellowish-red area or spot extending obliquely from each shoulder and a narrow one along suture in posterior half, base of abdomen, legs, antennae and maxillary palps yellowish brown (Fig. 331); integument moderately glossy, densely and coarsely punctate, especially on elytra and in tergal impressions; head much narrower than pronotum with large eyes, longer than temples, antennae with articles V–X increasingly broadening toward apex; pronotum sinuate basally and rounded laterally, broadest at middle and then abruptly narrowed apicad; elytra with prominent shoulders, broader than pronotum; abdomen subparallel, three basal tergites with deep impressions, each coarsely punctate. **Male.** Median lobe of aedeagus with bulbus moderately large, oval, tubus long, strongly produced ventrally, its ventral margin slightly sinuate, apex thin, narrow and acutely pointed in lateral view (Fig. 332); tergite VIII with apical margin broadly emarginate between two large lateral teeth, emargination weakly crenulate (Fig. 333); sternite VIII strongly, triangularly produced apically (Fig. 334). **Female.** Tergite VIII broadly truncate apically (Fig. 335); sternite VIII obtusely produced apically, with apex rounded (Fig. 336); spermatheca with capsule short, widely club shaped, stem narrow, curved (Fig. 337).

This species is externally similar to *Pleurotobia brunswickensis*, but its body is broader, more coarsely punctate, and less glossy, the integument is more reddish brown, and the median lobe of the aedeagus is shaped differently, with the venter less strongly sinuate in lateral view (Figs 318, 332).

######### Distribution.

Known from QC and NB, Canada.

######### Natural history.

*Pleurotobia bourdonae* was found in hardwood and mixed forests. Adults were found in *Hapalopilus nidulans* (Fr.) Kar. (Polyporaceae) on standing dead American beech (*Fagus grandifolia* Ehrh.) trees, in a *Pleurotus* sp. (Tricholomataceae) on a live sugar maple (*Acer saccharum* Marsh.), and in a decaying fleshy polypore (probably *Hapalopilus nidulans*) on a dead standing poplar. A description of the larva and biology of *Pleurotobia tristigmata* (Er.) [error for *Pleurotobia tristigma* Casey = *Pleurotobia trimaculata* (Er.)] is provided by Ashe (1990).

######### Comments.

The genus *Pleurotobia* Casey was previously represented in North America by one species, *Pleurotobia trimaculata* (Erichson) and its three synonyms, *Pleurotobia suturalis* Casey, *Pleurotobia tristigma* Casey, and *Pleurotobia texana* Casey (Ashe 1992). The illustration of the median lobe of the aedeagus and spermatheca of *Pleurotobia trimaculata* is provided by Ashe (1992). The two new species described in this paper are easily distinguishable from *Pleurotobia trimaculata* by the differently shaped median lobe of the aedeagus and the weak crenulation of the apical margin of male tergite VIII between two large lateral teeth (Figs 333, 340).

######## Pleurotobia brunswickensis

Taxon classificationAnimaliaColeopteraStaphylinidae

Klimaszewski & Webster sp. n.

http://zoobank.org/284F6D53-474C-43C7-A723-A48E44F78D7F

Figs 338–344

######### Holotype (male).

**Canada, New Brunswick, York Co.**, Canterbury near Browns Mtn. Fen, 45.8876°N, 67.6560°W, 3.VIII.2006, R.P. Webster, coll. // Hardwood forest, on slightly dried *Pleurotus* sp. on sugar maple (LFC). **Paratype: Canada, New Brunswick, Sunbury Co.**, Maugerville, Portobello Creek N.W.A., 45.8992°N, 66.4245°W, 18.VII.2004, R.P. Webster, coll. // Silver maple forest, on fleshy fungi (1 ♀, RWC).

######### Etymology.

This species name derives from the Canadian province of New Brunswick where the types were found.

######### Description.

**Figures 338–344.**
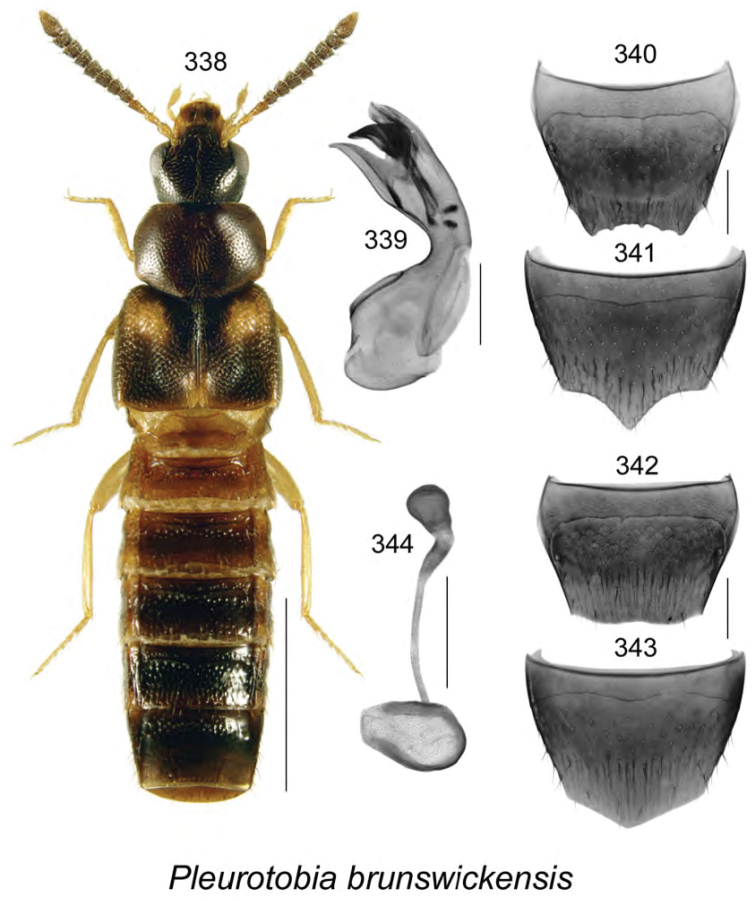
*Pleurotobia brunswickensis* Klimaszewski & Webster, sp. n.: **338** habitus in dorsal view **339** median lobe of aedeagus in lateral view **340** male tergite VIII **341** male sternite VIII **342** female tergite VIII **343** female sternite VIII **344** spermatheca. Scale bar of habitus = 1 mm; remaining scale bars = 0.2 mm.

Body length 3.9–4.0 mm, narrowly oval, robust, head, pronotum, most of elytra and posterior part of abdomen brownish black, elytra with a yellowish area or spot extending obliquely from each shoulder and a short, narrow longitudinal spot along suture apically, base of abdomen, legs, two basal antennal articles and maxillary palps yellowish (Fig. 338); integument strongly glossy, densely and coarsely punctate, especially on elytra and in tergal impressions; head much narrower than pronotum, eyes large, longer than temples, antennae with articles V–X increasingly broadening toward apex; pronotum sinuate basally and rounded laterally, broadest at middle and then abruptly narrowed apicad; elytra with prominent shoulders, broader than pronotum; abdomen subparallel, three basal tergites with deep impressions, each coarsely punctate. **Male.** Median lobe of aedeagus with bulbus moderately large, oval, tubus long, strongly produced ventrally, its ventral margin strongly sinuate, subapical section wide and apex thin, narrow and acutely pointed ventrally in lateral view (Fig. 339); apical margin of tergite VIII broadly, shallowly emarginate between two large lateral teeth, emargination weakly crenulate (Fig. 340); sternite VIII strongly, triangularly produced apically (Fig. 341). **Female.** Tergite VIII slightly sinuate apically (Fig. 342); sternite VIII obtusely produced apically, apex subangulate (Fig. 343) spermatheca with capsule short, widely club shaped, stem narrow, curved (Fig. 344).

This species is externally similar to *Pleurotobia bourdonae*, but has a narrower, less coarsely punctate and glossier body, and yellowish body color, the apical teeth of male tergite VIII are less prominent, and the median lobe of the aedeagus is differently shaped, with the venter strongly sinuate in lateral view (Figs 332, 339).

######### Distribution.

Known only from NB, Canada.

######### Natural history.

The holotype was found in a slightly dried *Pleurotus* mushroom on a sugar maple in an old hardwood forest in early August, the paratype was found in a fleshy fungus in a silver maple forest in July.

######### Comments.

See the previous species.

####### Subtribe Gyrophaenina Kraatz, 1856

######## Agaricomorpha vincenti

Taxon classificationAnimaliaColeopteraStaphylinidae

Klimaszewski & Webster sp. n.

http://zoobank.org/B284BD37-1501-4831-9788-0F6723ECD1A8

Figs 345–351

######### Holotype (male).

**Canada, New Brunswick, Carleton Co.**, Jackson Falls, “Bell Forest”, 46.2200°N, 67.7231°W, 7–21.VI.2012, C. Alderson & V. Webster, coll. // Rich Appalachian hardwood forest, Lindgren funnel trap in canopy of *Fagus grandifolia* (LFC). **Paratypes: Canada, New Brunswick, Carleton Co.**, Jackson Falls, “Bell Forest”, 46.2200°N, 67.7231°W, 1–8.VI.2009, R. Webster & M.-A. Giguère, coll. // Rich Appalachian hardwood forest with some conifers, Lindgren funnel trap (1 ♀, LFC); same data except 8–23.V.2012, C. Alderson & V. Webster // Lindgren funnel trap in canopy of *Acer saccharum* (1 ♀, RWC); same data except 17–31.VII.2012 // Lindgren funnel trap in canopy of *Juglans cinerea* (1 ♂, LFC). **Northumberland Co.**, ca. 1.5 km NW of Sevogle, 47.0939°N, 65.8387°W, 8–22.VII.2013, C. Alderson & V. Webster // *Populus tremuloides* stand with a few conifers, Lindgren funnel trap 1 m high under *Populus tremuloides* (1 ♀, RWC). **Restigouche Co.**, Dionne Brook P.N.A., 47.9030°N, 68.3503°W, 30.V-15.VI.2011, M. Roy & V. Webster // Old-growth northern hardwood forest, Lindgren funnel trap (1 ♀, RWC). **Sunbury Co.**, Gilbert Island, 45.8770°N, 66.2954°W, 23.V-6.VI.2013, C. Alderson, C. Hughes, & V. Webster // hardwood forest, Lindgren funnel trap in canopy of *Fraxinus pennsylvanica* (1 ♂, RWC).

######### Etymology.

This species is named in honor of Vincent Webster who collected a number of specimens of this species and many other species reported in this and other papers.

######### Description.

**Figures 345–351.**
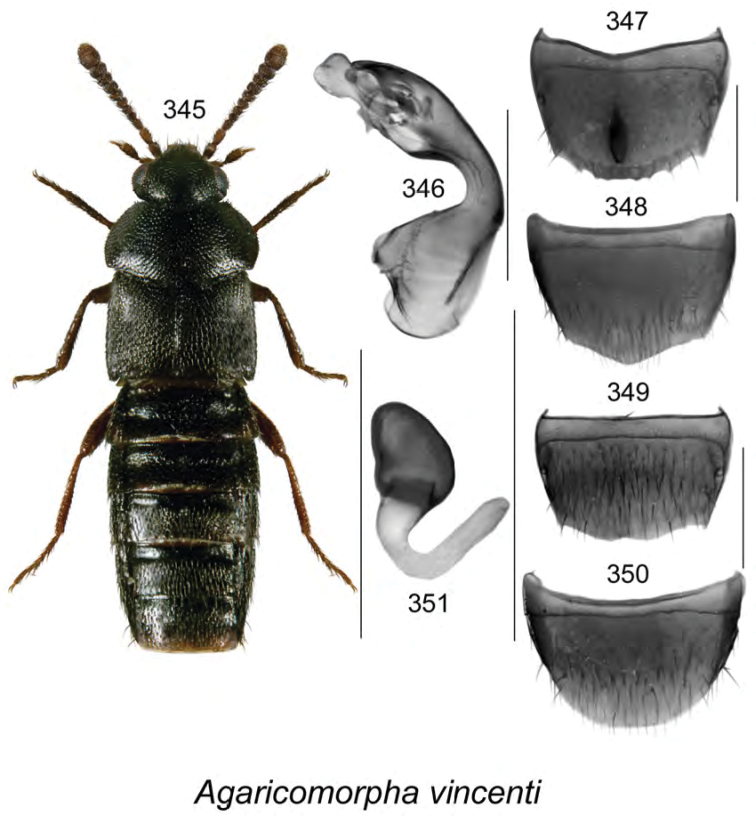
*Agaricomorpha vincenti* Klimaszewski & Webster, sp. n.: **345** habitus in dorsal view **346** median lobe of aedeagus in lateral view **347** male tergite VIII **348** male sternite VIII **349** female tergite VIII **350** female sternite VIII **351** spermatheca. Scale bar of habitus = 1 mm; remaining scale bars = 0.2 mm.

Body small, compact, and narrowly oval in outline; length 1.7–1.9 mm; body, antennae, and legs uniformly black (Fig. 345); forebody with strong microsculpture, that on elytra and abdomen coarse, scale-like, punctation coarse, sparse, and flatly impressed, pubescence sparse and approximately evenly distributed on forebody; head transverse, eyes large, postocular area reduced, pubescence directed posteriad and obliquely mesad; antennae incrassate, basal three antennomeres elongate, IV subquadrate, V–X increasingly broadening apically, XI oval and elongate; maxillary palpi with four articles, penultimate article expanded apically, and apical article acicular; pronotum strongly transverse, distinctly broader than elytra, base strongly sinuate, lateral edges abruptly converging apicad, disk with most pubescence directed posteriad, some at base directed laterad; elytra at suture as long as pronotum, pubescence directed straight posteriad; abdomen gradually but weakly tapering apically, tergites II and III strongly impressed basally, and with elevated punctures. **Male.** Median lobe of aedeagus with bulbus moderately large in lateral view, tubus U-shaped, narrow with broad and swollen apical part, flagellum long and thin (Fig. 346); tergite VIII transverse, apical margin arcuate, unevenly crenulate (Fig. 347); apical margin of sternite VIII obtusely angulate, broadly rounded medially (Fig. 348). **Female.** Tergite VIII transverse, sinuate apically with small median emargination (Fig. 349); sternite VIII transverse, apical margin subsemicircularly rounded (Fig. 350); spermatheca small, with capsule asymmetrical, narrowing toward apex, stem short, U-shaped (Fig. 351).

######### Distribution.

Known only from NB, Canada.

######### Natural history.

Specimens of *Agaricomorpha vincenti* were captured in Lindgren funnel traps in a rich Appalachian hardwood forest, a *Populus tremuloides* stand with a few conifers, an old-growth northern hardwood forest, and a hardwood forest on an island in a river. Nothing is known about the specific habitat requirements of this species. Adults were collected during May, June, and July in NB.

######### Comments.

This species may be readily distinguished from *Agaricomorpha websteri* Klimaszewski & Brunke by the differently shaped pronotum, which is distinctly broader than the elytra, by its uniformly black body, and by the shape of the median lobe of the aedeagus, male tergite VIII, and spermatheca (Figs 345, 346, 347, 351).(See Brunke et al. 2012 for details on *Agaricomorpha websteri*).

######## Gyrophaena (Gyrophaena) aldersonae

Taxon classificationAnimaliaColeopteraStaphylinidae

Klimaszewski & Webster sp. n.

http://zoobank.org/591DD88D-CD77-4E18-9619-5681645302D8

Figs 352–355

######### Holotype (male).

**Canada, New Brunswick, York Co.**, 15 km W of Tracy, off Rt 645, 45.6848 N, 66.8821°W, 21–28.VI.2009, R. Webster & M.-A. Giguère, coll. // Red pine forest, Lindgren funnel trap (LFC). **Paratypes: Canada, New Brunswick, Queens Co.**, Cranberry Lake P.N.A., 46.1125°N, 65.6075°W, 11–18.VI.2009, R. Webster & M.-A. Giguère, coll. // Red oak forest, Lindgren funnel trap (1 ♂, RWC); same data except 2.IX.2009, R.P. Webster, coll. // Red oak forest, polypore (bracket) fungus on side of log (1 ♀, RWC). **Restigouche Co.**, Dionne Brook P.N.A., 47.9030°N, 68.3503°W, 30.V-15.VI.2011, M. Roy & V. Webster // Old-growth northern hardwood forest, Lindgren funnel trap (1 ♂, RWC); same data except 28.VII-9.VIII.2011 (1 ♂, RWC). **York Co.**, Charters Settlement, 45.8395°N, 66.7391°W, 19.V.2006, R.P. Webster, coll. // Mixed forest, on polypore fungus on log (1 ♂, LFC).

######### Etymology.

This species is named in honor of Chantelle Alderson who helped collect many species reported in this and other papers.

######### Description.

**Figures 352–355.**
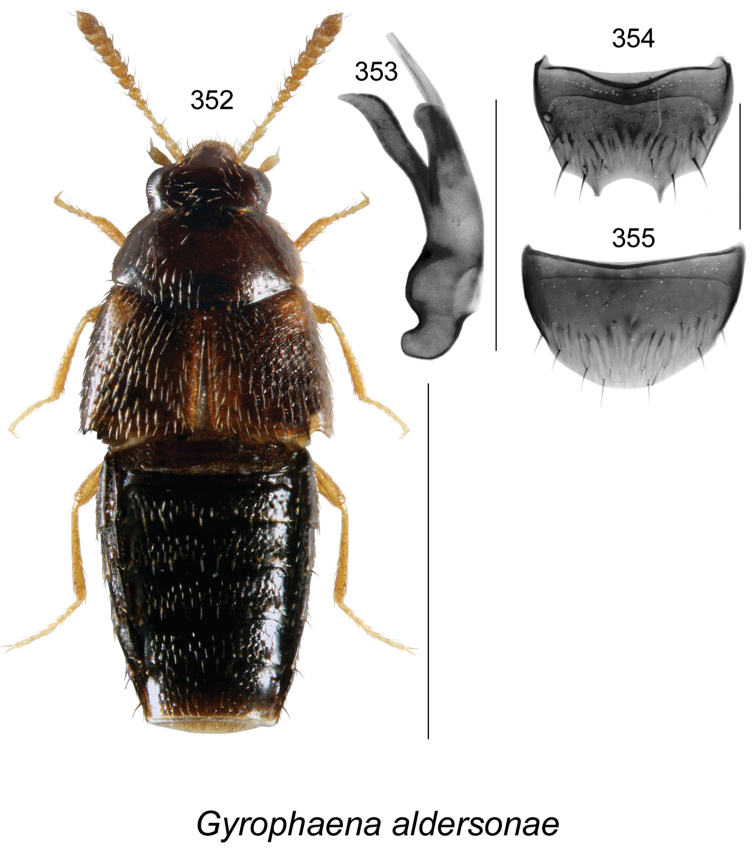
Gyrophaena (Gyrophaena) aldersonae Klimaszewski & Webster, sp. n.: **352** habitus in dorsal view **353** median lobe of aedeagus in lateral view **354** male tergite VIII **355** male sternite VIII. Scale bar of habitus = 1 mm; remaining scale bars = 0.2 mm.

Body length 1.7 mm, short, robust, oval, head, pronotum, elytra, and abdomen dark brown, elytra with small paler, reddish area on each shoulder and one along suture, appendages yellowish (Fig. 352); integument with weak meshed microsculpture on head and pronotum and strong on elytra, strongly glossy; pubescence short and sparse, appressed to integument; head small with protruding eyes, almost half as wide as pronotum; pronotum narrow, strongly transverse, broadest at base, almost as wide as elytra at base, and strongly narrowed apicad, pubescence directed posteriad; elytra broader than pronotum, widest posteriorly, pubescence directed posteriad; abdomen widest at base, tapering apicad. **Male.** Median lobe of aedeagus with tubus long, broad, and narrowly elongate, apex sharp, produced ventrally in lateral view (Fig. 353); tergite VIII transverse, apical margin with two acute pronounced teeth separated by about one-third width of tergite, with an arcuate emargination between them and shallower ones on either side (Fig. 354); sternite VIII transverse, evenly arcuate apically (Fig. 355). **Female.** Unknown.

######### Distribution.

Known only from NB, Canada.

######### Natural history.

*Gyrophaena aldersonae* were captured in Lindgren funnel traps in a red oak forest and an old-growth northern hardwood forest. Two individuals were collected from a polypore (bracket) fungus on the sides of logs. Adults were collected from May to September.

######### Comments.

*Gyrophaena aldersonae* is a distinct species in the Nearctic fauna, and males have a uniquely shaped tergite VIII (Fig. 354) and median lobe of the aedeagus in lateral view (Fig. 353). The shape of the median lobe and apical part of male tergite VIII are somewhat similar to those of *Gyrophaena joyioides* Wüsthoff reported from Croatia and the Caucasus (Lohse *in* Lohse 1974, Seevers 1951).

######## Gyrophaena (Gyrophaena) brevicollis

Taxon classificationAnimaliaColeopteraStaphylinidae

Seevers, 1951

Figs 356–362

######### Material examined.

**New Brunswick, Sunbury Co.**, McGowans Corner, Grand Lake P.N.A., 45.8959°N, 66.2823°W, 16.VI.2013, R.P. Webster // Silver maple forest, in *Polyporus squamosus* (on dead standing silver maple) (1 ♂, 1 ♀, RWC).

######### Natural history.

Two individuals of *Gyrophaena brevicollis* were collected from *Polyporus squamosus* (Polyporaceae) on a dead standing silver maple in a silver maple forest. One specimen from ON was collected from gilled mushrooms (Brunke et al. (2012), otherwise little is known about the habitat association of this species.

######### Distribution in Canada and Alaska.

ON, **NB** (Bousquet et al. 2013). Brunke et al. (2012) reported this species for the first time for Canada from several sites in southern ON.

######### Comments.

Except for a slight difference in the shape of male tergite VIII, the NB specimen agrees with the description and illustrations in Seevers (1951) for *Gyrophaena brevicollis*. We have noted that the shape of the male tergite is variable in other *Gyrophaena* species (Klimaszewski et al. 2009b).

**Figures 356–362.**
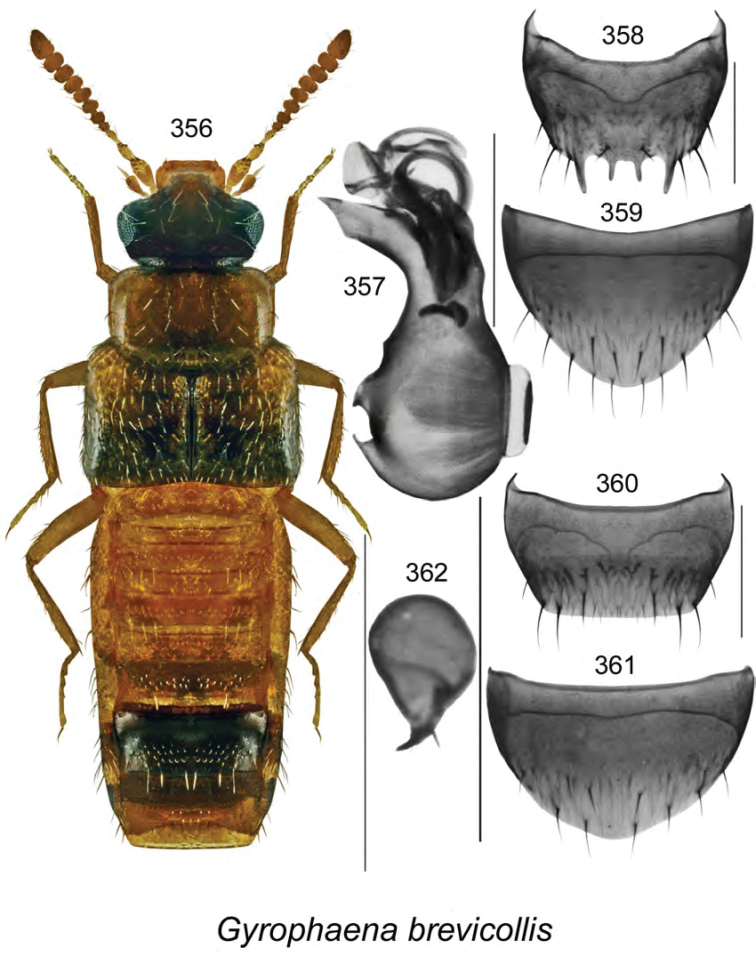
Gyrophaena (Gyrophaena) brevicollis Seevers: **356** habitus in dorsal view **357** median lobe of aedeagus in lateral view **358** male tergite VIII **359** male sternite VIII **360** female tergite VIII **361** female sternite VIII **362** spermatheca. Scale bar of habitus = 1 mm; remaining scale bars = 0.2 mm.

####### Subtribe Homalotina Heer, 1839

######## Anomognathus americanus

Taxon classificationAnimaliaColeopteraStaphylinidae

(Casey, 1894)

Figs 363–366

######### Material examined.

**Canada, New Brunswick, Restigouche Co.**, Dionne Brook P.N.A., 47.9030 N, 68.3503°W, 30.V.-15.VI.2011, M. Roy & V. Webster, coll. // Old-growth northern hardwood forest, Lindgren funnel trap (1 ♀, RWC).

######### Distribution in Canada and Alaska.

**(New Canadian record).** Apparently the species has not been found in North America since Casey’s original description of specimens from NY; it was treated as a synonym of *Anomognathus cuspidatus* Erichson by Fenyes (1918), but this has to be confirmed.

**Figures 363–366.**
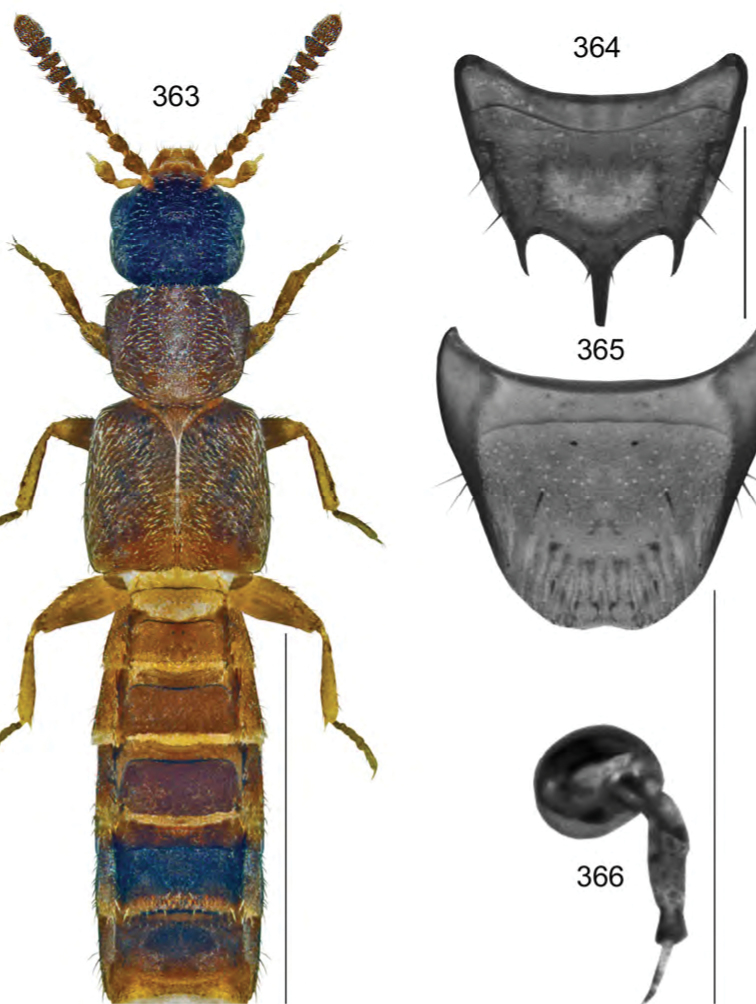
*Anomognathus americanus* (Casey): **363** habitus in dorsal view **364** female tergite VIII **365** female sternite VIII **366** spermatheca. Scale bar of habitus = 1 mm; remaining scale bars = 0.2 mm.

###### Tribe Hoplandriini Casey, 1910

####### Subtribe Hoplandriina Casey, 1910

######## Hoplandria (Lophomucter) laevicollis

Taxon classificationAnimaliaColeopteraStaphylinidae

(Notman, 1920)

Figs 367–374

######### Material examined.

**New Brunswick, Northumberland Co.**, ca, 2.5 km W of Sevogle, 47.0876°N, 65.8613°W, 21.VIII.2013, 27.VIII.2013, R.P. Webster // Old *Pinus banksiana* forest, in rotten boletus mushrooms (8 ♀, RWC); ca. 1.5 km NW of Sevogle, 47.0939°N, 65.8387°W, 6–21.VIII.2013, C. Alderson & V. Webster // *Populus tremuloides* stand with a few conifers, Lindgren funnel trap 1 m high under *Populus tremuloides* (1 ♀, RWC).

######### Natural history.

Most adults of *Hoplandria laevicollis* from NB were found in rotten bolete mushrooms in an old jack pine forest. One individual was captured in a Lindgren funnel trap in a stand of trembling aspen. Adults were collected during August.

######### Distribution in Canada and Alaska.

ON, QC, **NB** (Génier 1989; Brunke et al. 2012; Bousquet et al. 2013).

######### Comments.

All specimens of *Hoplandria laevicollis* from NB were females. The identification was based on the description and key in Génier (1989). It should be noted that female characters are not as diagnostic as those of males, and thus the determination of these specimens should be considered as provisional until males are obtained from the sites where the species was found.

**Figures 367–374.**
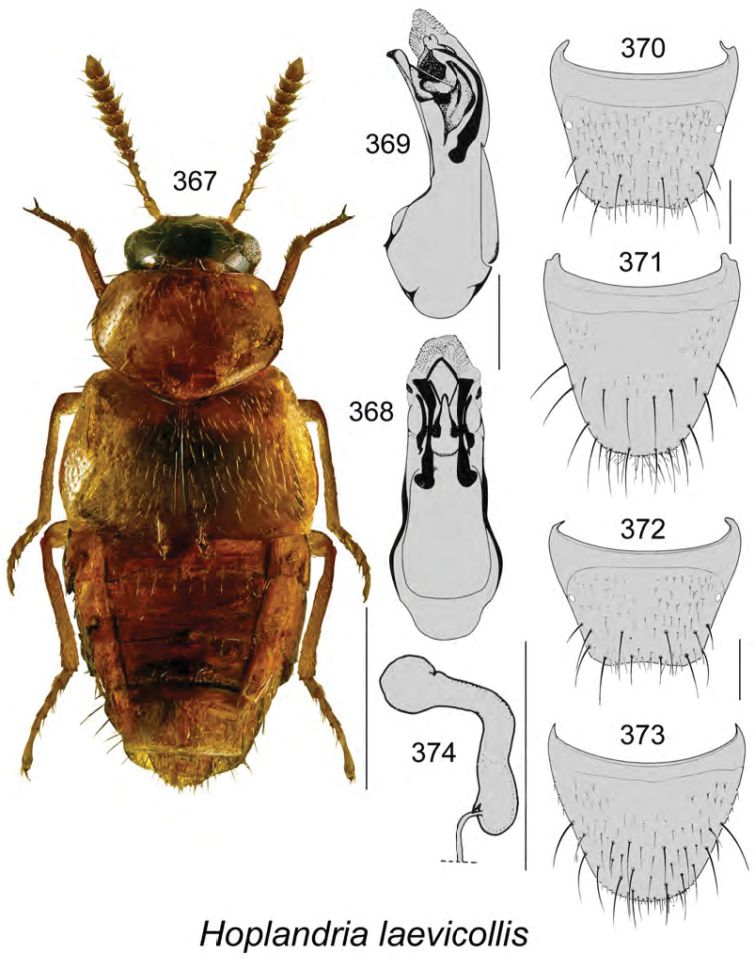
Hoplandria (Lophomucter) laevicollis (Notman): **367** habitus in dorsal view **368** median lobe of aedeagus in dorsal view **369** median lobe of aedeagus in lateral view **370** male tergite VIII **371** male sternite VIII **372** female tergite VIII **373** female sternite VIII **374** spermatheca. Scale bar of habitus = 1 mm; remaining scale bars = 0.2 mm.

###### Tribe Hypocyphtini Laporte, 1835

####### Oligota chrysopyga

Taxon classificationAnimaliaColeopteraStaphylinidae

Kraatz, 1859†

Figs 375–380

######## Material examined.

**Canada, New Brunswick, York Co.**, Charters Settlement, 45.8395°N, 66.7391°W, 18.X.2007, 3.IX.2010, 7.IX.2010, 19.IX.2010, 22.IX.2010, R.P. Webster, coll. // Mixed forest, in decaying (moldy) corncobs & cornhusks (1 ♂, 3 ♀, 6 sex undetermined, RWC); same data but 7.IX.2010 (1 ♀, 1 sex undetermined, LFC).

######## Natural history.

*Oligota chrysopyga* was common in a pile of decaying and moldy corncobs and cornhusks near a composter in a residential area adjacent to a mixed forest. Not much is known about the biology of *Oligota*. Frank et al. (1992) mentions that some species prey on mites, which were abundant in the moldy corncobs and cornhusks where the NB specimens were collected.

######## Distribution in Canada and Alaska.

**NB (New Canadian record).** Although now considered cosmopolitan, the only other North American record of this adventive species was by Frank (1976) from FL, where it was apparently introduced from the Caribbean.

**Figures 375–380.**
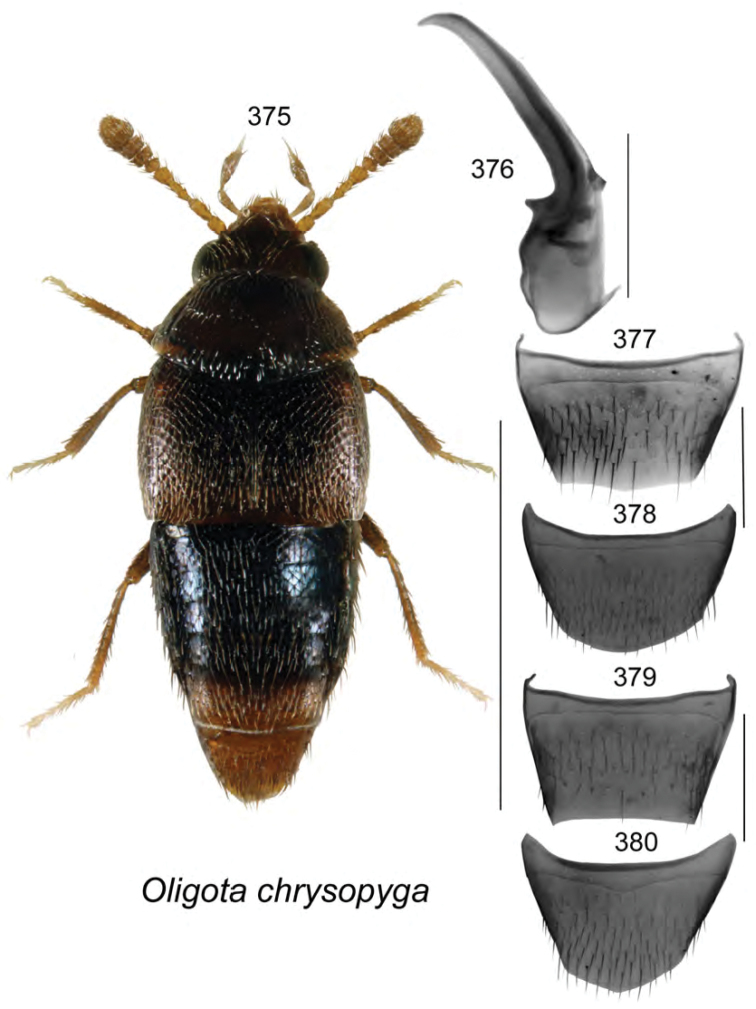
*Oligota chrysopyga* Kraatz: **375** habitus in dorsal view **376** median lobe of aedeagus in lateral view **377** male tergite VIII **378** male sternite VIII **379** female tergite VIII **380** female sternite VIII. Scale bar of habitus = 1 mm; remaining scale bars = 0.2 mm.

####### Oligota parva

Taxon classificationAnimaliaColeopteraStaphylinidae

Kraatz, 1862†

Figs 381–386

######## Material examined.

**New Brunswick, York Co.**, Charters Settlement, 45.8395°N, 66.7391°W, 5.X.2007, 26.IX.2008, 5.IX.2009, 3.IX.2010, 19.IX.2010, R.P. Webster, coll. // Mixed forest, in decaying (moldy) corncobs & cornhusks (5 ♂, 5 ♀, RWC); same data but 3.IX.2010, 7.IX.2010 (2 ♂, 1 ♀, LFC).

######## Natural history.

*Oligota parva* was common in a pile of decaying and moldy corncobs and cornhusks near a composter in a residential area adjacent to a mixed forest. Mites were abundant in the moldy corncobs and cornhusks where the specimens were collected. Majka et al. (2008) reported this species from sea beach drift at the top of the littoral zone on PE. Adults were collected during September and October.

######## Distribution in Canada and Alaska.

**NB**, PE (Bousquet et al. 2013). Majka et al. (2008) reported this adventive species from Canada for the first time from PE.

**Figures 381–386.**
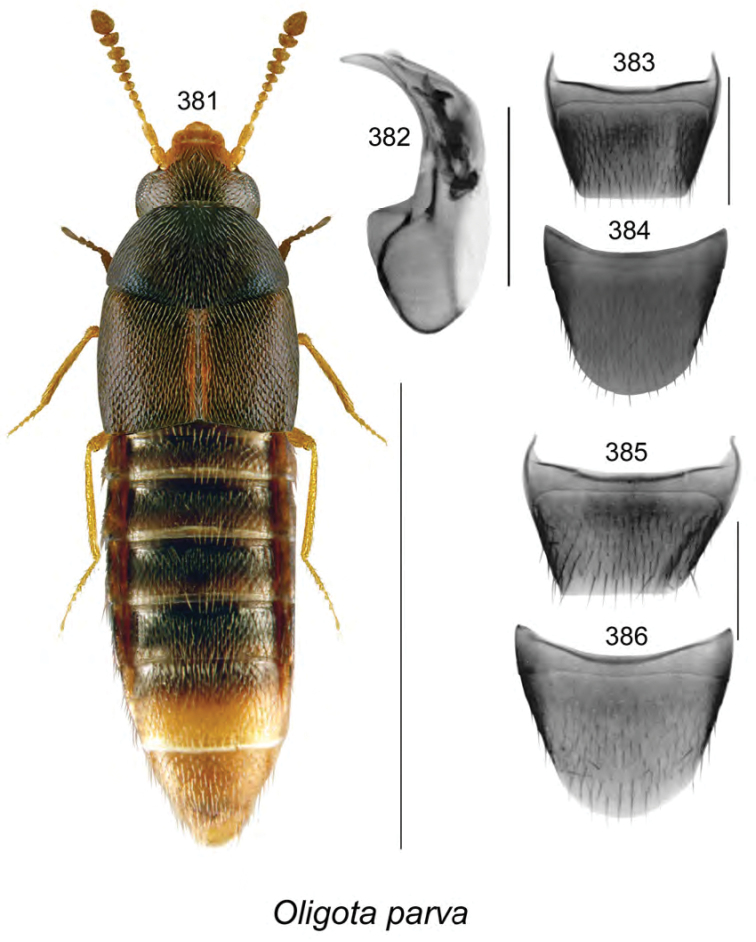
*Oligota parva* Kraatz: **381** habitus in dorsal view **382** median lobe of aedeagus in lateral view **383** male tergite VIII **384** male sternite VIII **385** female tergite VIII **386** female sternite VIII. Scale bar of habitus = 1 mm; remaining scale bars = 0.2 mm.

####### Oligota polyporicola

Taxon classificationAnimaliaColeopteraStaphylinidae

Klimaszewski & Webster sp. n.

http://zoobank.org/EC6B980E-A346-4187-89D3-9BB2CF2D0018

Figs 387–393

######## Holotype (male).

**Canada, New Brunswick, Sunbury Co.**, Acadia Research Forest, 45.9799°N, 66.3394°W, 18.VI.2007, R.P. Webster, coll. // Road 7 control, mature red spruce and red maple forest, fleshy polypore fungi on stump (LFC). **Paratypes: Canada, New Brunswick, Carleton Co.**, Wakefield, Meduxnekeag Valley Nature Preserve, 46.1907°N, 67.6740°W, 15.VI.2006, R.P. Webster, coll. // Hardwood forest, on fleshy polypore (bracket) fungi on dead standing beech (1 ♂, AFC; 1 ♂, 1 ♀, LFC; 1 sex undetermined, RWC); Jackson Falls, “Bell Forest Nature Preserve”, 46.2199°N, 67.7231°W, 7.VI.2007, R.P. Webster, coll. // Rich Appalachian hardwood forest, in polypore fungi on large fallen basswood (1 ♂, 2 ♀, RWC); same data but 9.X.2006 // Hardwood forest, on fleshy polypore fungi on dead standing beech (1 ♂, CNC; 1 ♀, LFC). **Sunbury Co.**, Acadia Research Forest, 45.9799°N, 66.3394°W, 18.VI.2007, R.P. Webster, coll. // Road 7 control, mature red spruce and red maple forest, fleshy polypore fungi on stump (3 ♂, 1 ♀, 1 sex undetermined, RWC). **York Co.**, New Maryland, Charters Settlement, 45.8286°N, 66.7365°W, 22.VI.2008, R.P. Webster, coll. // Mixed forest, in polypore fungus on *Populus* log (1 ♂, RWC).

######## Etymology.

Named after polypore mushrooms where the holotype and many of the paratypes were found.

######## Description.

**Figures 387–393.**
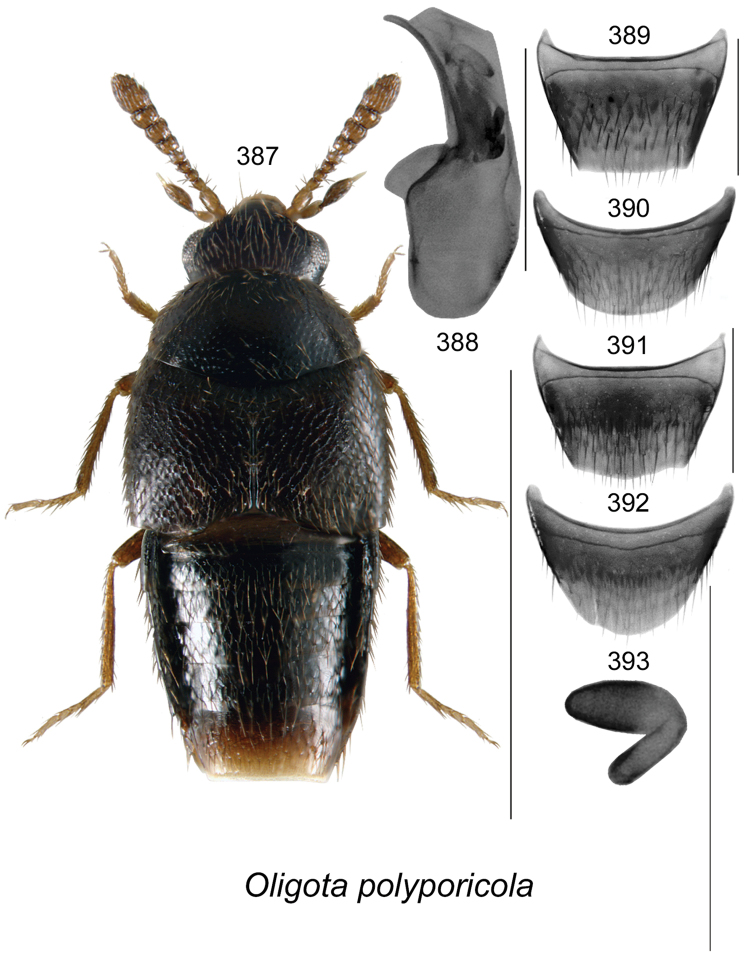
*Oligota polyporicola* Klimaszewski & Webster, sp. n.: **387** habitus in dorsal view **388** median lobe of aedeagus in lateral view **389** male tergite VIII **390** male sternite VIII **391** female tergite VIII **392** female sternite VIII **393** spermatheca. Scale bar of habitus = 1 mm; remaining scale bars = 0.2 mm.

Body length 1.4–1.5 mm, short, compact, broadly oval, piceous brown to black, with legs, antennae, maxillary palps, and tip of abdomen reddish brown (Fig. 387); forebody moderately and abdomen strongly glossy; integument with microsculpture mesh-like on head and pronotum, coarse, scale-like on elytra and less so on abdomen; pubescence sparse and long; head transverse with large protruding eyes, pubescence directed anteriad; antennae with four apical articles broad and forming loose club, articles VI–VII moderately transverse; pronotum strongly transverse, lateral margins strongly converging apically, pubescence directed posteriad on midline of disk and obliquely laterad elsewhere; elytra broad, arcuate laterally with pubescence directed obliquely laterad; abdomen tapering apicad. **Male.** Median lobe of aedeagus with tubus long, arcuate apically, apex thin and produced ventrally in lateral view, bulbus moderately long with large carina apicalis (Fig. 388); internal sac structures as illustrated (Fig. 388); tergite VIII truncate apically (Fig. 389); sternite VIII broadly arcuate apically (Fig. 390). **Female.** Tergite VIII with apical margin very broadly obtusely angulate (Fig. 391); sternite VIII rounded apically (Fig. 392); spermatheca with capsule elongate-oval in apical half, angularly bent at middle (Fig. 393).

######## Natural history.

This species was found in hardwood forests, a mixed forest, and a mature red spruce and red maple forest. Adults were found in polypore fungi on dead standing American beeches, a large fallen basswood, a *Populus* log, and on a stump. Specimens occurred within the tubes of the polypore fungi. Adults were collected during June and October.

######## Distribution.

Known only from NB, Canada.

######## Comments.

We have checked the world literature on the genus and compared all available genital illustrations and found none matching our species, which led to the conclusion that it was undescribed (Williams 1970a, 1970b, 1972, 1973a, 1973b, 1975, 1976, 1979, Frank 1972, Lohse 1974, Frank et al. 1992, Assing 1995, 2003). In addition, we consulted J.H. Frank, who studied American and Caribbean types and species of *Oligota*, and he confirmed that our species was not among the species he studied.

####### Oligota pusillima

Taxon classificationAnimaliaColeopteraStaphylinidae

Gravenhorst, 1806†

Figs 394–400

######## Material examined.

**Canada, New Brunswick, York Co.**, Charters Settlement, 45.8395°N, 66.7391°W, 30.IX.2007, 5.X.2007, 27.IV.2008, 8.VIII.2010, 7.IX.2010, R.P. Webster, coll. // Mixed forest, in decaying (moldy) corncobs & cornhusks (2 ♂, 4 ♀, RWC); same data but 5.X.2007, 3.IX.2010 (1 ♂, 1 ♀, LFC).

######## Natural history.

*Oligota pusillima* was found in a pile of decaying and moldy corncobs and cornhusks near a composter in a residential area adjacent to a mixed forest. Mites were abundant in the moldy corncobs and cornhusks where the specimens were collected. Adults were collected during April, August, September, and October.

######## Distribution in Canada and Alaska.

**NB (New Canadian record).**

######## Comments.

*Oligota pusillima* is considered a cosmopolitan species (Smetana 2004). It was known in the USA from MA (Fauvel 1889) and NY (synonyms *Oligota linearis* Casey and *Oligota parallela* Casey), but it was never before recorded from Canada.

**Figures 394–400.**
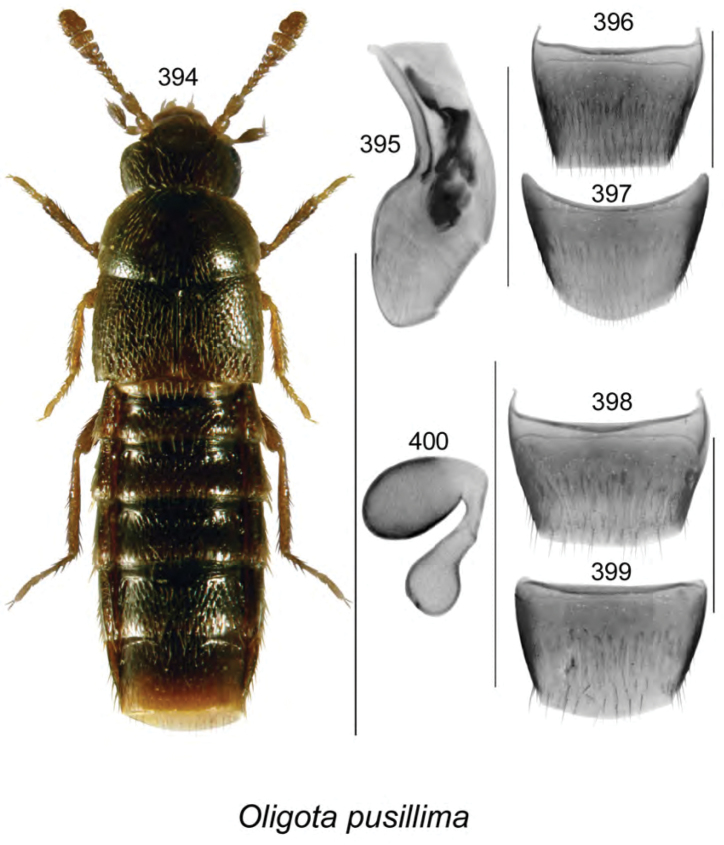
*Oligota pusillima* Gravenhorst: **394** habitus in dorsal view **395** median lobe of aedeagus in lateral view **396** male tergite VIII **397** male sternite VIII **398** female tergite VIII **399** female sternite VIII **400** spermatheca. Scale bar of habitus = 1 mm; remaining scale bars = 0.2 mm.

####### Oligota sevogle

Taxon classificationAnimaliaColeopteraStaphylinidae

Klimaszewski & Webster sp. n.

http://zoobank.org/A7DB3175-8184-4038-99D2-7573FD4A8688

Figs 401–407

######## Holotype (male).

**Canada, New Brunswick, Northumberland Co**., ca. 2.5 km W of Sevogle, 47.0876°N, 65.8613°W, 1–14.V.2013, C. Alderson & V. Webster // Old *Pinus banksiana* forest, Lindgren funnel trap (LFC). **Paratypes: Canada, New Brunswick, Northumberland Co**., ca. 2.5 km W of Sevogle, 47.0876°N, 65.8613°W, 1–14.V.2013, 31.V-15.VI.2013, C. Alderson & V. Webster // Old *Pinus banksiana* forest, Lindgren funnel traps (1 ♂, 5 ♀, RWC); same data but 1–14.V.2013 (1 ♀, LFC). **Restigouche Co.**, Dionne Brook P.N.A., 47.9064°N, 68.3441°W, 31.V–15.VI.2011, M. Roy & V. Webster // Old-growth white spruce & balsam fir forest, Lindgren funnel trap (1 ♂, RWC); same data but 15–27.VI.2011 (1 ♀, LFC). **York Co.**, 15 km W of Tracy off Rt. 645, 45.6848°N, 66.8821°W, 8–15.VI.2009, 2–20.VI.2011, M. Roy & V. Webster // Old red pine forest, Lindgren funnel trap (1 ♂, RWC).

######## Etymology.

Named after the village of Sevogle near where the type and most of the paratypes were collected, in apposition.

######## Description.

**Figures 401–407.**
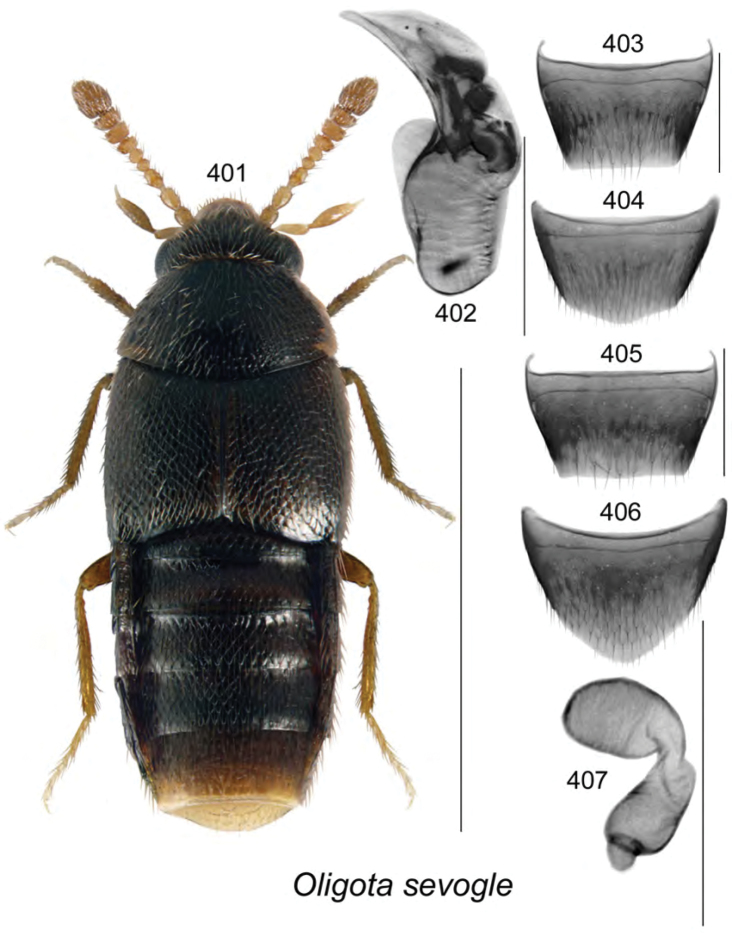
*Oligota sevogle* Klimaszewski & Webster, sp. n.: **401** habitus in dorsal view **402** median lobe of aedeagus in lateral view **403** male tergite VIII **404** male sternite VIII **405** female tergite VIII **406** female sternite VIII **407** spermatheca. Scale bar of habitus = 1 mm; remaining scale bars = 0.2 mm.

Body length 1.4–1.5 mm, short, compact, moderately broadly oval, piceous to nearly black, with legs or only tarsi, antennae, maxillary palps, and tip of abdomen yellowish brown (Fig. 401); moderately glossy; integument with microsculpture mesh-like on head and pronotum, coarse, scale-like on elytra and abdomen; pubescence sparse and long; head transverse with eyes large, protruding, pubescence directed anteriad; antennae with four apical articles forming loose club, articles VI–VII narrow and strongly transverse; pronotum strongly transverse, lateral margins strongly converging apicad, pubescence directed obliquely laterad; elytral margins broadly arcuate laterally with pubescence directed obliquely laterad; abdomen gradually narrowed apicad. **Male.** Median lobe of aedeagus with tubus long, arcuate, apex moderately thin and gradually arched ventrally in lateral view, bulbus moderately long with large carina apicalis (Fig. 402); internal sac structures as illustrated (Fig. 402); tergite VIII truncate apically (Fig. 403); sternite VIII with apical margin very obtusely angulate, broadly rounded medially (Fig. 404). **Female.** Tergite VIII truncate apically (Fig. 405); sternite VIII broadly parabolic apically (Fig. 406); spermatheca broad, with capsule oval in apical half, constricted and bent at middle (Fig. 407).

######## Natural history.

Adults were collected from Lindgren funnel traps in a jack pine forest (most), a red pine forest, and an old-growth white spruce and balsam fir forest. Specimens were captured during May and June.

######## Distribution.

Known only from NB, Canada.

######## Comments.

We have checked the world literature on the genus and compared all available genital illustrations and found none matching our species, which led to the conclusion that it was undescribed (Williams 1970a, 1970b, 1972, 1973a, 1973b, 1975, 1976, 1979, Frank 1972, Lohse in Lohse 1974, Frank et al. 1992, Assing 1995, 2003). In addition, we consulted J.H. Frank, who studied American and Caribbean types and species of *Oligota*, and he confirmed that our species was not among the species he studied.

###### Tribe Oxypodini C.G. Thomson, 1859

####### Subtribe Dinardina Mulsant & Rey, 1873

######## Blepharhymenus brendeli

Taxon classificationAnimaliaColeopteraStaphylinidae

(Casey, 1894)

Figs 408–414

######### Material examined.

**New Brunswick, Albert Co.**, Caledonia Gorge P.N.A., 45.7808°N, 64.7775°W, 4.VII.2011, R.P. Webster // Canada Creek, cold, clear, shaded rocky brook with small waterfalls, sifting saturated moss on rocks near flowing water (1 ♂, 1 sex undetermined, NBM); Caledonia Gorge P.N.A., 45.8432°N, 64.8411°W, 5.VII.2011, R.P. Webster // Turtle Creek, rocky cool water & shaded creek, in saturated moss on rocks (1 sex undetermined, NBM); same locality, collection date and collector but 45.8385°N, 64.8435°W // Old-growth hardwood forest, cold, clear, shaded rocky brook with small waterfalls, sifting saturated moss on rocks near flowing water (1 sex undetermined, NBM); Caledonia Gorge P.N.A., 45.8176°N, 64.7800°W, 6.VII.2011, R.P. Webster // Mature hardwood forest, mossy seepage with *Carex*, sifting saturated moss and *Carex* litter (1 sex undetermined, NBM); Caledonia Gorge P.N.A., 45.7706°N, 64.8063°W, 12.IX.2012, R.P. Webster & M.-A. Giguère // McKinely Brook, in leaf litter in areas with *Carex* along brook (1 sex undetermined, NBM; 1 sex undetermined, RWC); **Charlotte Co.**, 3.0 km NW of Pomeroy Ridge, 45.3059°N, 67.4343°W, 16.VI.2008, R.P. Webster, coll. // Old-growth eastern white cedar swamp, in moss & leaf litter near small vernal pools (1 ♀, LFC; 1 sex undetermined, RWC); ca. 9 km NW of New River, 45.2096°N, 66.6483°W, 13.VI.2008, R.P. Webster, coll. // Alder swamp near large brook, in grass & leaf litter (1 ♂, RWC). **Northumberland Co.**, ca, 2.5 km W of Sevogle, 47.0876°N, 65.8613°W, 14–28.V.2013, C. Alderson & V. Webster // Old *Pinus banksiana* stand, Lindgren funnel trap (1 sex undetermined, AFC). **Restigouche Co.**, Jacquet River Gorge P.N.A., 47.7146°N, 66.1644°W, 24.VI.2008, R.P. Webster, coll. // Alder swamp adjacent to slow flowing brook, in leaves on muddy soil (1 ♂, 1 sex undetermined, RWC). **Saint John Co.**, ca. 2 km NE of Maces Bay, 45.1151°N, 66.4553°W, 8.V.2006, R.P. Webster, coll. // Eastern white cedar swamp, in sphagnum & litter near brook (1 ♂, LFC; 1 ♂, 1 ♀, RWC). **York Co.**, New Maryland, off Hwy 2, E of Baker Brook, 45.8760°N, 66.6252°W, 4.VI.2005, R.P. Webster, coll. // Old-growth cedar swamp in moss & litter (1 ♂, LFC); Charters Settlement, 45.8341°N, 66.7445°W, 22.IV.2005, R.P. Webster, coll. // Mature spruce & cedar forest, seepage area, in saturated sphagnum & leaf litter (1 ♀, RWC); same locality but 45.8395°N, 66.7391°W, 20.V.2010, R.P. Webster // Alder swamp, small brook, under cobblestones (1 sex undetermined, RWC); Rt. 645 at Beaver Brook, 45.6840°N, 66.8679°W, 3.V.2008, R.P. Webster, coll. // Red maple/alder swamp, in moist leaves near small vernal pools near small stream (1 ♀, RWC); Keswick Ridge, 45.9962°N, 66.8781°W, 5–19.V.2015, C. Alderson & V. Webster // Mixed forest, Lindgren funnel trap 1 m high under trees (1 sex undetermined, NBM); Kouchibouguac N. P., 10.VII.1977, I. Smith, code 6065G (1 ♂, 1 ♀, CNC); Kouchibouguac N. P., 18.V.1977, S.J. Miller, code 5070Z (1 ♀, CNC). **Nova Scotia**, Cape Breton Highlands National Park, Fishing Cove Trail, 21.VI.1984, A. Smetana (3 ♂, 2 ♀, CNC); Cape Breton H.N.P., nr. Mary Ann Falls, 22.VI.1984, A. Smetana (1 ♂, CNC); Cape Breton H.N.P., 12 m, Warren Lake Trail, PG982768, 26.IX.1984, J.M. Campbell & A. Davies, sifting litter and moss (1 ♀, CNC); Halifax, Sackville, No. 181, 20.V.1951, Lindroth (2 ♀, CNC). **Ontario, Peterborough Co.**, Warsaw, 5–8.IX.1974, I.M. Smith (1 ♀, CNC); Whitney, Nipissing Dist., 19.IX.1974, I.M. Smith (2 ♀, CNC); Peterborough Co., Warsaw Caves, Conservation area, 9.VI.1975, I.M. Smith (1 ♀, CNC); Carleton Co., 6 mi W. Richmond, 15.IX.1974, I.M. Smith (1 ♀, CNC); Ottawa River Deschênes Lookout, 1.V.1985, A. Davies, Berlese flood debris (1 ♀, CNC); Nepean NCC Log Farm, 1.XI.1985, A. Davies, ex Salix litter at edge of beaver pond (1 ♀, CNC). **Québec, Co. Vaudreuil**, Rigaud end Ch. de la Croix, 5.V.1988, 950, A. and Z. Smetana (1 ♀, CNC); same data except 952 (1 ♂, 5 ♀, CNC); Gatineau Pk., Fortune Lk., 28.VIII.1982, Lohse & Campbell (3 ♂, 3 ♀, CNC); Gatineau Pk., Ramsay Lake area, 12.IX.1970, J.M. Campbell (1 ♀, CNC).

######### Natural history.

In NB, *Blepharhymenus brendeli* adults were found near shaded brook and stream margins, near vernal pools near brooks, and in mossy seepage areas in hardwood forests, old-growth eastern white cedar forests and swamps, in a mature spruce and cedar forest, and in alder swamps. Specimens were found in saturated moss on rocks near flowing water, in saturated moss and *Carex* litter in seepages, in leaf litter in areas with *Carex* near brooks, in moss and litter, and in moss, sphagnum, and leaf litter near brooks in the above habitats. The QC specimens were collected in an oak–beech–maple forest, by sifting deep, moldy leaf litter along bases of large rock blocks, and in a small seepage under a hydro line with large ferns, dogwood and *Salix*, and by sifting layers of moist dead fern leaves and detritus under ferns. Nothing was previously known about the habitat associations of this species. Adults were collected during April, May, June, July, and September.

######### Distribution in Canada and Alaska.

**ON**, **QC**, **NB**, **NS (New Canadian record).**

**Figures 408–414.**
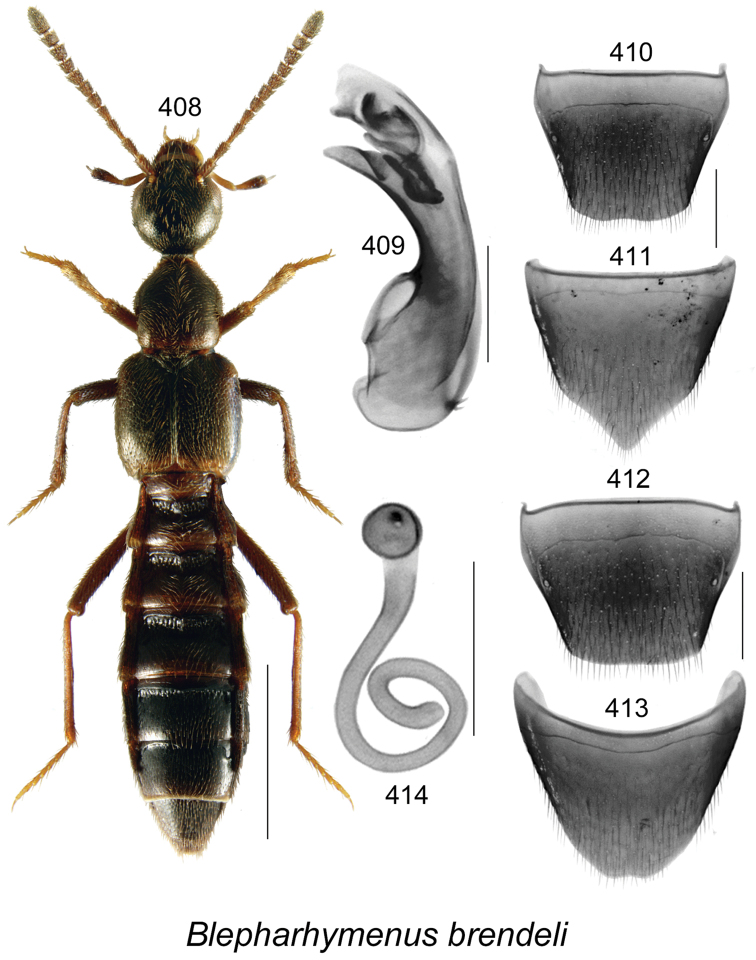
*Blepharhymenus brendeli* (Casey): **408** habitus in dorsal view **409** median lobe of aedeagus in lateral view **410** male tergite VIII **411** male sternite VIII **412** female tergite VIII **413** female sternite VIII **414** spermatheca. Scale bar of habitus = 1 mm; remaining scale bars = 0.2 mm.

####### Subtribe Meoticina Seevers, 1978

######## Meotica pallens

Taxon classificationAnimaliaColeopteraStaphylinidae

(Redtenbacher, 1849)†

Figs 415–423

Meotica pallens (For diagnosis, see Klimaszewski et al. 2007)

######### Material examined.

**New Brunswick, Restigouche Co.**, Dionne Brook P.N.A., 47.9030°N, 68.3503°W, 30.V-15.VI.2011, M. Roy & V. Webster // Old-growth northern hardwood forest, Lindgren funnel trap (1 ♀, RWC). **York Co.**, Charters Settlement, 45.8395°N, 66.7391°W, 2.V.2010, R.P. Webster // Mixed forest opening, collected with net during evening flight between 16:30 and 20:00 h (2 ♀, RWC); same locality and collector but 45.8331°N, 66.7279°W, 20.V.2010 // Beaver dam, among sticks, debris, and clay on dam (1 ♂, RWC).

######### Natural history.

Adults were collected with a net in a mixed forest opening during the evening, and sifted from among sticks, debris and clay on a beaver dam. One individual was caught in a Lindgren funnel trap in an old-growth northern hardwood forest. This species was collected during May and June in NB.

######### Distribution in Canada and Alaska.

BC, ON, **NB**, NS (Klimaszewski et al. 2007; Majka and Klimaszewski 2008b; Bousquet et al. 2013).

**Figures 415–423.**
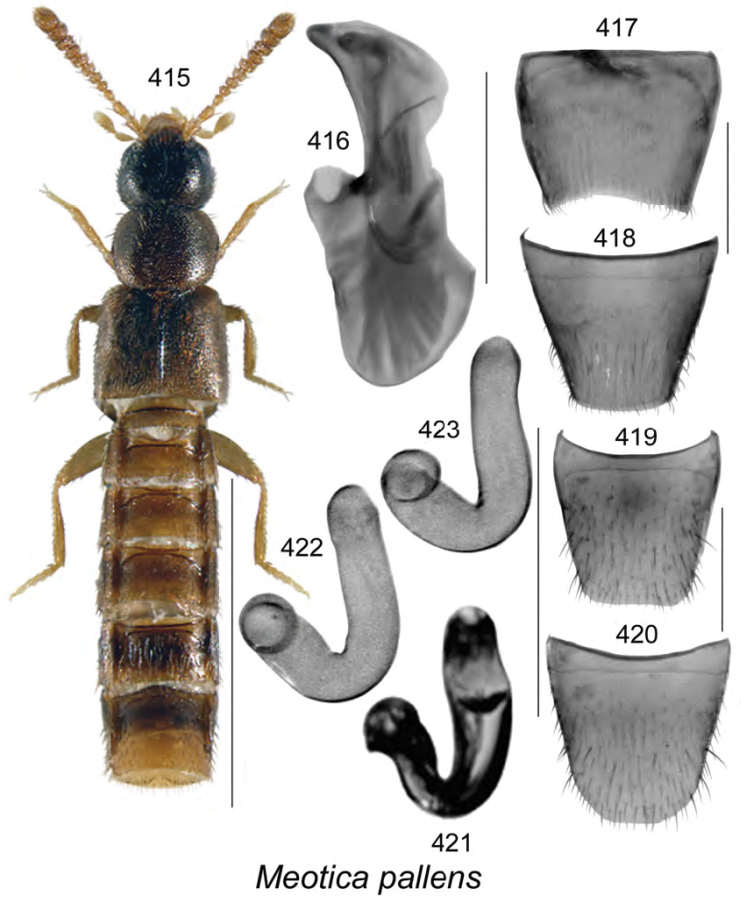
*Meotica pallens* (Redtenbacher): **415** habitus in dorsal view **416** median lobe of aedeagus in lateral view **417** male tergite VIII **418** male sternite VIII **419** female tergite VIII **420** female sternite VIII **421–423** spermatheca. Scale bar of habitus = 1 mm; remaining scale bars = 0.2 mm.

####### Subtribe Oxypodina C.G. Thomson, 1859

######## Calodera caseyi

Taxon classificationAnimaliaColeopteraStaphylinidae

Assing, 2002

Figs 424–427

Calodera caseyi (For diagnosis, see Assing 2008)

######### Material examined.

**New Brunswick, York Co.**, 14 km WSW of Tracy, S of Rt. 645, 45.6741°N, 66.8661°W, 10–26.V.2010, R. Webster & C. MacKay, coll. // Old mixed forest, Lindgren funnel trap (1 ♀, RWC).

######### Distribution in Canada and Alaska.

**NB (New Canadian record).**

######### Comments.

The male of this species is unknown.

**Figures 424–427.**
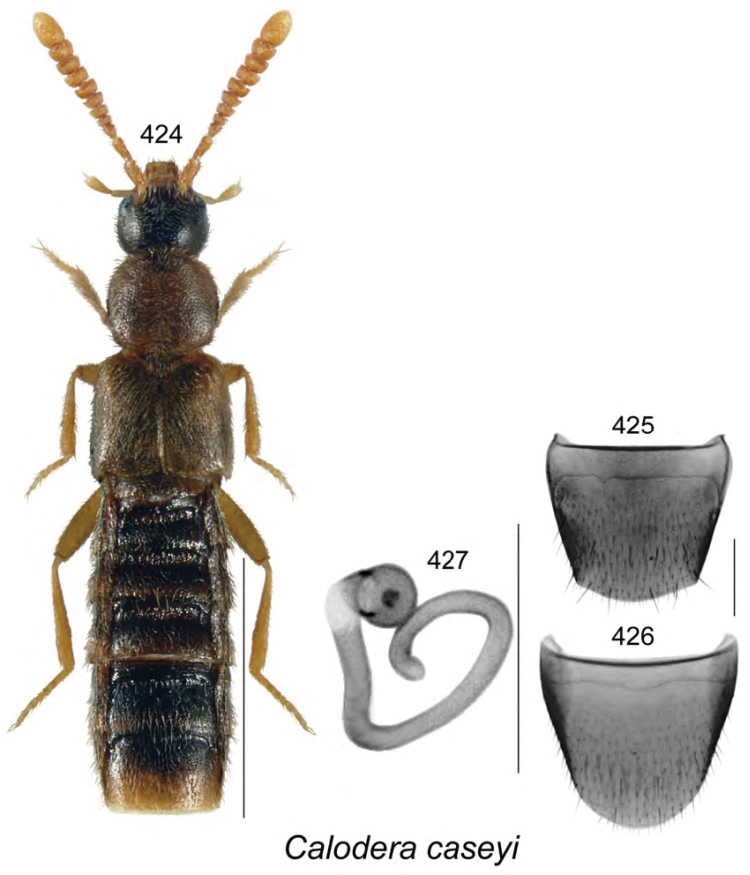
*Calodera caseyi* Assing: **424** habitus in dorsal view **425** female tergite VIII **426** female sternite VIII **427** spermatheca. Scale bar of habitus = 1 mm; remaining scale bars = 0.2 mm.

######## Dexiogyia angustiventris

Taxon classificationAnimaliaColeopteraStaphylinidae

(Casey, 1894)

Figs 428–435

Dexiogyia angustiventris (For diagnosis, see Brunke et al. 2012)

######### Material examined.

**New Brunswick, Albert Co.**, Caledonia Gorge P.N.A., 45.7786°N, 64.8068°W, 2.VII.2011, R.P. Webster // McKinely Brook, old-growth sugar maple & yellow birch forest, in *Tricholomopsis platyphylla* (Pers.) Sing. (1 ♂, RWC). **Sunbury Co.**, Gilbert Island, 45.8770°N, 66.2954°W, 12–29.VI.2012, C. Alderson, C. Hughes & V. Webster // Hardwood forest, Lindgren funnel trap in canopy of *Populus tremuloides* (1 ♀, RWC). **York Co.**, Charters Settlement, 45.8286°N, 66.7365°W, 2.VI.2007, R.P. Webster, coll. // Mature red spruce forest, under bark of red spruce (1 ♀, RWC); 16 km W of Tracy, off Rt. 645, 45.6855°N, 66.8847°W, 18.V-2.VI.2010, R. Webster & C. MacKay, coll. // Old red pine forest, Lindgren funnel trap (1 ♀, RWC); Keswick Ridge, 45.9962°N, 66.8781°W, 4–19.VI.2014, C. Alderson & V. Webster // Field/meadow, Lindgren funnel trap 1 m high (1 ♂, RWC).

######### Natural history.

Specimens of *Dexiogyia angustiventris* from NB were caught in Lindgren funnel traps in a hardwood forest in the canopy of *Populus tremuloides*, an old red pine forest, and in an open field and meadow. One individual was collected from *Tricholomopsis platyphylla* (Pers.) Sing., on a log in an old-growth sugar maple and yellow birch forest and another from under bark of red spruce in a mature red spruce stand. Brunke et al. (2012) reported specimens from under bark of white pine in ON. According to Seevers (1978) *Dexiogyia* is associated with subcortical microhabitats, particularly pine, and occurs in burrows of wood-boring Coleoptera.

######### Distribution in Canada and Alaska.

ON, **NB** (Bousquet et al. 2013). Brunke et al. (2012) reported *Dexiogyia angustiventris* for the first time for Canada based on specimens from ON.

**Figures 428–435.**
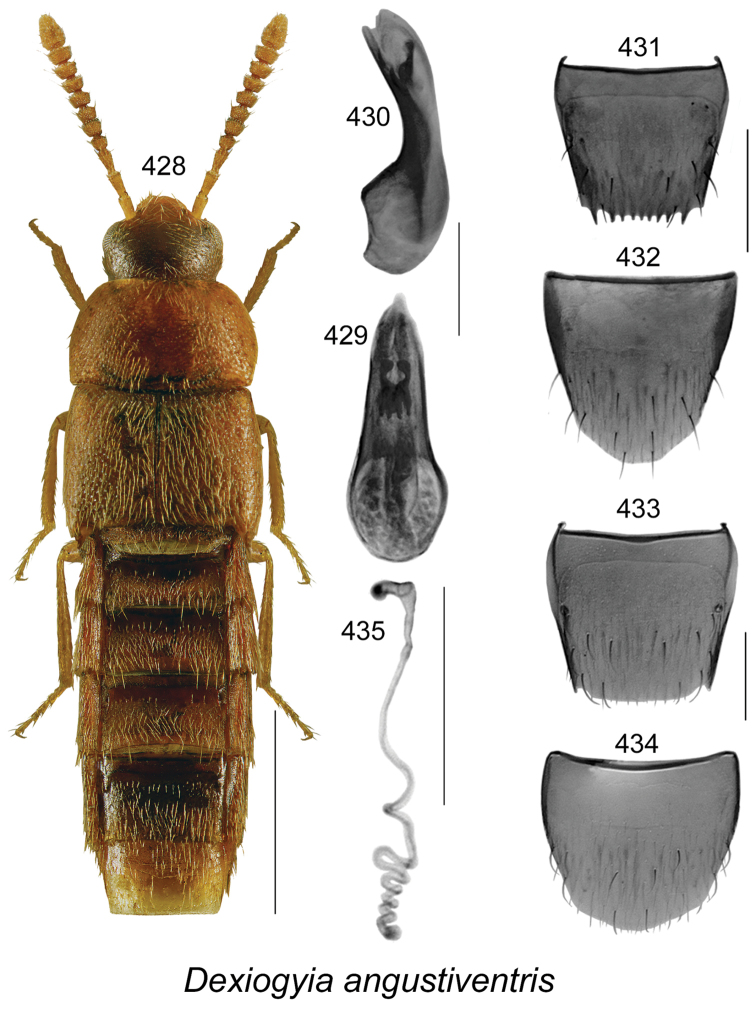
*Dexiogyia angustiventris* (Casey): **428** habitus in dorsal view **429** median lobe of aedeagus in dorsal view **430** median lobe of aedeagus in lateral view **431** male tergite VIII **432** male sternite VIII **433** female tergite VIII **434** female sternite VIII **435** spermatheca. Scale bar of habitus = 1 mm; remaining scale bars = 0.2 mm.

######## Hylota cryptica

Taxon classificationAnimaliaColeopteraStaphylinidae

Klimaszewski & Webster sp. n.

http://zoobank.org/DEFBFA82-F2F3-49F3-9C92-C979E53396DE

Figs 436–442

######### Holotype (male).

**Canada, New Brunswick, York Co.**, 15 km W of Tracy, off Rt. 645, 45.6848°N, 66.8821°W, 8–20.VI.2011, M. Roy & V. Webster, coll. // Old red pine forest, flight intercept trap (LFC). **Paratypes: Canada, New Brunswick, Queens Co.**, Cranberry Lake P.N.A., 46.1125°N, 65.6075°W, 13–25.V.2011, M. Roy & V. Webster // Red oak forest, Lindgren funnel trap (1 ♀, RWC); C.F.B. Gagetown, 45.7516°N, 66.1866°W, 4–17.VI.2013, C. Alderson & V. Webster // Old mixed forest with *Quercus rubra*, Lindgren funnel traps in canopy of *Quercus rubra* (2 ♂, 1 ♀, RWC). **Northumberland Co.**, ca, 2.5 km W of Sevogle, 47.0876°N, 65.8613°W, 14–28.V.2013, C. Alderson & V. Webster // Old *Pinus banksiana* stand, Lindgren funnel trap (1 ♀, AFC). **Restigouche Co.**, Dionne Brook P.N.A., 47.9030°N, 68.3503°W, 15–27.VI.2011, M. Roy & V. Webster // Old-growth northern hardwood forest, Lindgren funnel trap (1 ♀, RWC); Dionne Brook P.N.A., 47.9064°N, 68.3441°W, 27.VI-14.VII.2011, M. Roy & V. Webster // Old-growth white spruce & balsam fir forest, flight intercept traps (1 ♀, LFC; 1 ♀, RWC). **Sunbury Co.**, Gilbert Island, 45.8770°N, 66.2954°W, 25.VII-8.VIII.2012, C. Alderson, C. Hughes & V. Webster // Hardwood forest, in canopy of *Tilia americana* (1 ♀, RWC); same data but 12–29.VI.2012 (1 ♀, LFC). **York Co.**, 14 km WSW of Tracy, S of Rt. 645, 45.6741°N, 66.8661°W, 2–16.VI.2010, R. Webster & C. MacKay, coll. // Old mixed forest with red & white spruce, red & white pine, balsam fir, eastern white cedar, red maple, and *Populus* sp., Lindgren funnel trap (1 ♀, LFC); same data except 16–30.VI.2010 (1 ♀, RWC); 16 km W of Tracy, off Rt. 645, 45.6855°N, 66.8847°W, 18.V-2.VI.2010, R. Webster & C. MacKay // Old red pine forest, Lindgren funnel trap (1 ♂, RWC); 15 km W of Tracy, off Rt. 645, 45.6848°N, 66.8821°W, 16–30.VI.2010, R. Webster & C. MacKay, coll. // Old red pine forest, Lindgren funnel trap (1 ♀, RWC); Douglas, Currie Mountain, 45.9832°N, 66.7564°W, 19.VIII-6.IX.2013, C. Hughes & A. Morrison // Old *Pinus strobus* stand, Lindgren funnel trap in canopy of *Pinus strobus* (1 ♀, RWC); Keswick Ridge, 45.9962°N, 66.8781°W, 4–19.VI.2014, C. Alderson & V. Webster // Mixed forest, Lindgren funnel trap 1 m high under trees (1 sex undetermined, NBM).

######### Etymology.

*Cryptica* is a Latin feminine adjective meaning concealed, in allusion to similarity to its sibling species, *Hoplandria ochracea*.

######### Description.

**Figures 436–442.**
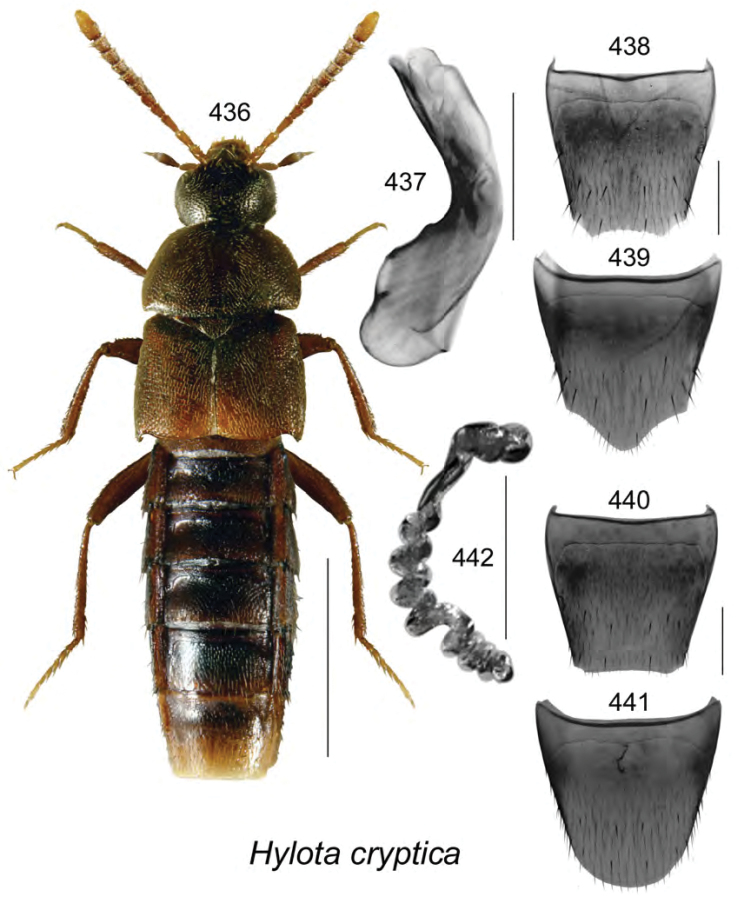
*Hylota cryptica* Klimaszewski & Webster, sp. n.: **436** habitus in dorsal view **437** median lobe of aedeagus in lateral view **438** male tergite VIII **439** male sternite VIII **440** female tergite VIII **441** female sternite VIII **442** spermatheca. Scale bar of habitus = 1 mm; remaining scale bars = 0.2 mm.

Body length 3.2–3.4 mm, narrowly oval, dark brown except antennae, tarsi, and posterior part of elytra near suture paler (Fig. 436); forebody densely punctate and pubescent; head about one-third of maximum pronotal width; antennal articles IV–X from slightly elongate to subquadrate; pronotum broadest at basal third and strongly narrowed apicad, at base as wide as elytra; elytra transverse and slightly longer than pronotum; abdomen arcuate laterally and tapering toward apex. **Male.** Median lobe of aedeagus with tubus strongly bent ventrally in lateral view (Fig. 437), (similar but less strongly produced ventrally in *Oligota ochracea* Casey); male tergite VIII broadly emarginate apically, with minute crenulation (Fig. 438) (with more pronounced teeth in *Hylota ochracea*); sternite VIII subtriangularly produced apically with apex rounded (Fig. 439). **Female.** Tergite VIII truncate apically, margin entire (Fig. 440); sternite VIII semicircularly rounded apically (Fig. 441); spermatheca with capsule small, sac-shaped and semispherical apically, stem with about eight to nine tight coils (Fig. 442).

######### Distribution.

Known only from NB, Canada.

######### Natural history.

All specimens of *Hylota cryptica* were captured in Lindgren funnel traps or flight intercept traps in various forest types. These included a red oak forest, an old mixed forest with red oak, mixed forests, a hardwood forest on an island in a river, an old-growth northern hardwood forest, an old-growth white spruce and balsam fir forest, an old jack pine forest, an old red pine forest, and an old white pine stand. Nothing is known about the specific habitat requirements of this species.

######### Comments.

This cryptic species may be separated from *Hylota ochracea* by its larger, broader and darker body, pronotum at least as wide as elytra at base (slightly narrower in *Hylota ochracea*), elongate antennal articles V–X (transverse in *Hylota ochracea*), tubus of median lobe less bent laterally, apical margin of male tergite VIII with minute crenulation (with teeth in *Hylota ochracea*), and spermatheca with fewer coils (8–9 in *Hylota cryptica* and about 15–17 in *Hylota ochracea*).

######## Mniusa

Taxon classificationAnimaliaColeopteraStaphylinidae

Mulsant & Rey, 1875

######### Comments.

Klimaszewski et al. (2014) reviewed the Canadian species of *Mniusa* and *Ocyusa*. In this review, *Mniusa minutissima* Klimaszewski & Langor was reported for the first time for NB and *Mniusa odelli* Klimaszewski & Webster was described as a new species. Klimaszewski et al. (2014) illustrated the male genitalia (Fig. 7b, c, d) of a specimen tentatively determined as *Mniusa odelli* but did not include it and other specimens from NS and QC in the type series because of the poor quality of the specimens. An additional male specimen confirmed as *Mniusa odelli* was found at the type locality in NB during 2014. The male genitalia conform to those illustrated in Klimaszewski et al. (2014) and are illustrated here along with the adult habitus. In light of this discovery, all NB specimens of *Mniusa* were reexamined, and it became apparent that some specimens originally determined as *Mniusa minutissima* were actually *Mniusa odelli*. Below, we present new and corrected data for the distribution of *Mniusa minutissima* and *Mniusa odelli* in NB that reflect these changes and additions.

######## Mniusa minutissima

Taxon classificationAnimaliaColeopteraStaphylinidae

(Klimaszewski & Langor, 2011)

Figs 443–449

Mniusa minutissima (For diagnosis, see Klimaszewski et al. 2011)

######### Material examined.

**Additional New Brunswick record, York Co.**, Canterbury, Eel River P.N.A., 45.8967°N, 67.6343°W, 21.V-2.VI.2014, C. Alderson & V. Webster // Old-growth eastern white cedar swamp & fen, Lindgren funnel trap (1 ♂, RWC).

######### Natural history.

The original specimens of *Mniusa minutissima* from NB were found by sifting moss near a brook and sifting deep conifer litter at the base of a large red spruce in a mature red spruce forest (Klimaszewski et al. 2014). Previous records of this species captured from Lindgren funnel traps in a rich Appalachian hardwood forest reported by Klimaszewski et al. (2014) were *Mniusa odelli* (see below). An additional record of *Phloeopora minutissima* from NB is reported here from a Lindgren funnel trap in an old-growth eastern white cedar swamp and fen.

######### Distribution in Canada and Alaska.

NB, NF (Klimaszewski et al. 2014).

**Figures 443–449.**
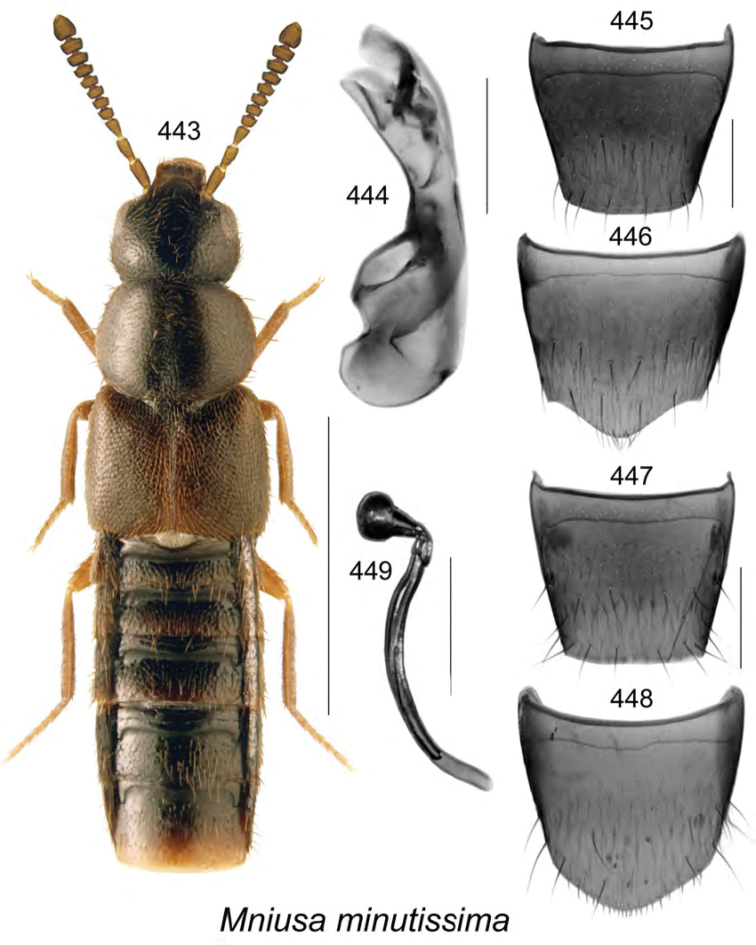
*Mniusa minutissima* (Klimaszewski & Langor): **443** habitus in dorsal view **444** median lobe of aedeagus in lateral view **445** male tergite VIII **446** male sternite VIII **447** female tergite VIII **448** female sternite VIII **449** spermatheca. Scale bar of habitus = 1 mm; remaining scale bars = 0.2 mm.

######## Mniusa odelli

Taxon classificationAnimaliaColeopteraStaphylinidae

Klimaszewski & Webster, 2014

Figs 450–456

Mniusa odelli (For details, see Klimaszewski et al. 2014)

######### Material examined.

**Additional New Brunswick records, Carleton Co.**, Jackson Falls, “Bell Forest”, 46.2200°N, 67.7231°W, 6–12.VI.2008, R.P. Webster, coll. // Rich Appalachian hardwood forest with some conifers, Lindgren funnel trap (1 ♀, RWC); same data but 1–8.VI.2009, R. Webster & M.-A. Giguère, coll. (2 ♂, RWC); same data but 8–16.VI.2009, M.-A. Giguère & V. Webster (1 ♂, RWC). **Restigouche Co.**, Jacquet River Gorge P.N.A., 47.8257°N, 66.0764°W, 22.VII-5.VIII.2014, C. Alderson & V. Webster // Old *Populus balsamifera* stand near river, Lindgren funnel trap 1 m high under trees (1 ♀, NBM). **York Co.**, Fredericton, Odell Park, 45.9484°N, 66.6802°W, 22.V-4.VI.2014, C. Alderson & V. Webster // Old mixed forest, Lindgren funnel trap 1 m under trees (1 ♂, RWC).

######### Natural history.

*Mniusa odelli* was originally described from specimens captured in Lindgren traps in an old-growth eastern hemlock forest. Additional specimens were collected in Lindgren funnel traps in a rich Appalachian hardwood forest (originally determined as *Mniusa minutissima*), an old *Populus balsamifera* stand near a river, and in an old mixed forest. Nothing is known about the specific habitat requirements of this species.

######### Distribution in Canada and Alaska.

NB (Klimaszewski et al. 2014).

**Figures 450–456.**
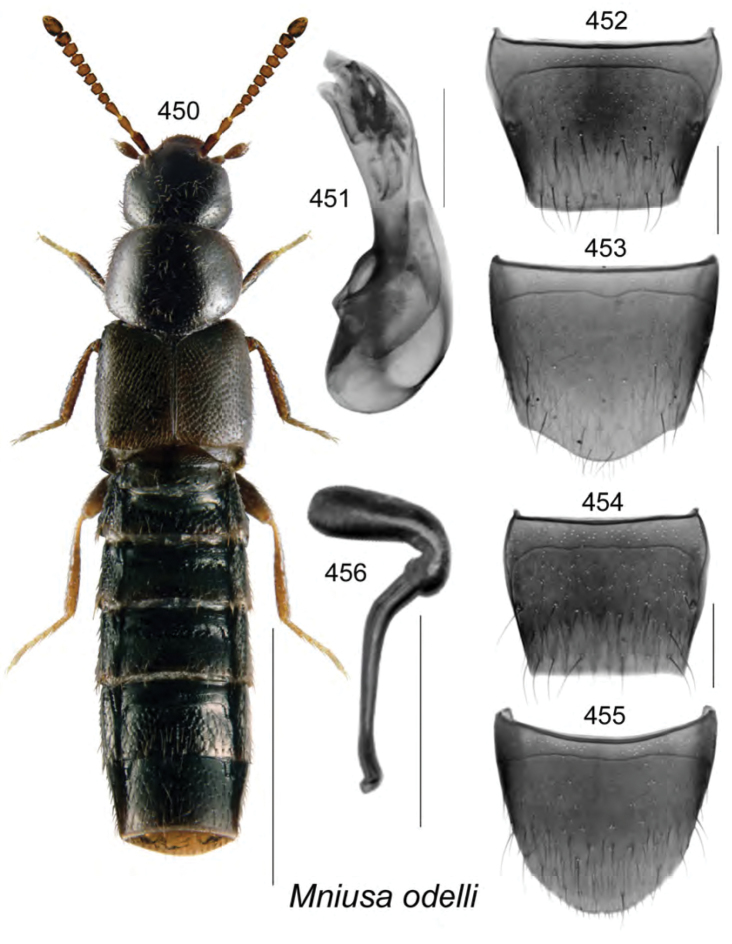
*Mniusa odelli* Klimaszewski & Webster: **450** habitus in dorsal view **451** median lobe of aedeagus in lateral view **452** male tergite VIII **453** male sternite VIII **454** female tergite VIII **455** female sternite VIII **456** spermatheca. Scale bar of habitus = 1 mm; remaining scale bars = 0.2 mm.

######## Neothetalia canadiana

Taxon classificationAnimaliaColeopteraStaphylinidae

Klimaszewski, 2004

Figs 457–464

Neothetalia canadiana (For diagnosis, see Klimaszewski and Pelletier 2004)

######### Material examined.

**New Brunswick, Restigouche Co.**, Dionne Brook P.N.A., 47.9064°N, 68.3441°W, 15–27.VI.2011, M. Roy & V. Webster // Old-growth white spruce & balsam fir forest, Lindgren funnel trap (1 ♀, RWC).

######### Natural history.

Klimaszewski et al. (2011) reported *Neothetalia canadiana* from pitfall traps in coastal barrens in NF. In other areas, it had been found in gopher burrows, carrion, and in litter on the forest floor in a white spruce feather moss forest (Klimaszewski and Pelletier 2004). The sole NB specimen was captured in a Lindgren funnel trap in an old-growth white spruce and balsam fir forest.

######### Distribution in Canada and Alaska.

AK, YT, BC, QC, **NB**, LB (Klimaszewski and Pelletier 2004; Klimaszewski et al. 2011; Bousquet et al. 2013).

**Figures 457–464.**
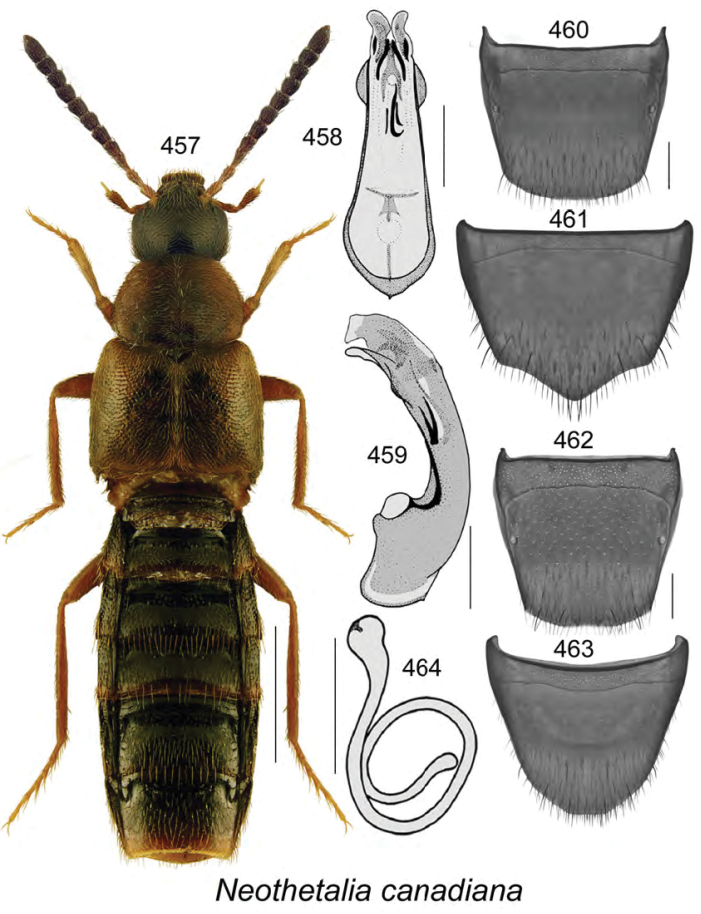
*Neothetalia canadiana* Klimaszewski: **457** habitus in dorsal view **458** median lobe of aedeagus in dorsal view **459** median lobe of aedeagus in lateral view **460** male tergite VIII **461** male sternite VIII **462** female tergite VIII **463** female sternite VIII **464** spermatheca. Scale bar of habitus = 1 mm; remaining scale bars = 0.2 mm.

######## Oxypoda sunpokeana

Taxon classificationAnimaliaColeopteraStaphylinidae

Klimaszewski & Webster sp. n.

http://zoobank.org/EF41B0B6-EB76-415E-872D-E872AECEC320

Figs 465–472

######### Holotype (male).

**Canada, New Brunswick, Restigouche Co.**, NE of jct Little Tobique Rd. and Red Bk., 47.4458°N, 67.0616°W, 13.VI.2006, R.P. Webster, coll. // Alder swamp with eastern white cedar, in moss and grass litter near brook (LFC). **Paratypes: Canada, New Brunswick, Queens Co.**, Upper Gagetown, bog adjacent to Hwy 2, 45.8324°N, 66.2350°W, 3.VII.2010, R.P. Webster, coll. // Tamarack bog, treading *Carex*, leather-leaf, & sphagnum on bog margin (1 ♀, RWC). **Sunbury Co.**, Burton, SW of Sunpoke Lake, 45.7875°N, 66.5736°W, 17.IV.2005, R.P. Webster, coll., // Red maple swamp, in leaf litter near margin of slow stream (1 ♀, LFC **York Co.**, Charters Settlement, 45.8427°N, 66.7234°W, 9.V.2004, R.P. Webster, coll. // Abandoned beaver pond, in moist grass litter on muddy soil (1 ♀, RWC); Kingsclear, Mazerolle Settlement, 45.8729°N, 66.8311°W, 28.IV.2006, R.P. Webster, coll. // Stream margin, in grass litter on muddy soil (1 ♂, LFC); Rt. 645 at Beaver Brook, 45.6860°N, 66.8668°W, 6.V.2008, R.P. Webster, coll. // *Carex* marsh, in litter at base of dead red maple (1 ♂, 2 ♀, RWC); 8.5 km W of Tracy, off Rt. 645, 45.6821°N, 66.7894°W, 6.V.2008, R.P. Webster, coll. // Alder swamp, in moist litter & grass on hummocks near water (1 ♀, RWC); 9.2 km W of Tracy, off Rt. 645, 45.6837°N, 66.8809°W, 22.V.2008, R.P. Webster, coll. // *Carex* marsh adjacent to slow stream, in *Carex* hummock (2 ♂, RWC); 14 km WSW of Tracy, S of Rt. 645, 45.6603°N, 66.8607°W, 2.V.2010, R.P. Webster, coll. // Black spruce bog, in sphagnum hummocks with *Carex* and grasses (1 ♂, RWC).

######### Etymology.

This species is named after Sunpoke Lake where one of the paratypes was collected.

######### Description.

**Figures 465–472.**
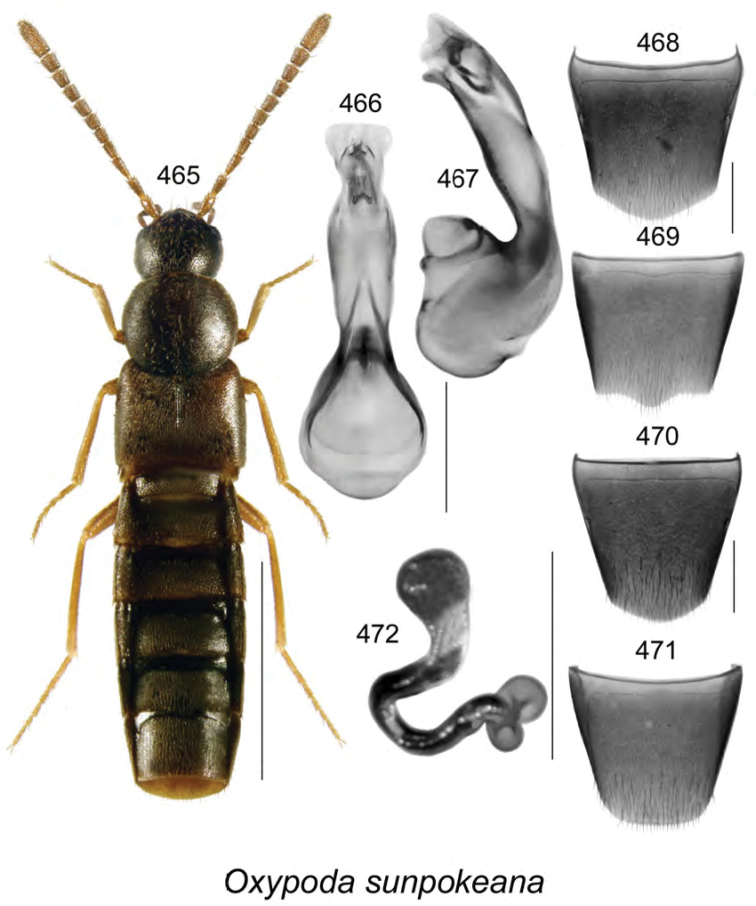
*Oxypoda sunpokeana* Klimaszewski & Webster, sp. n.: **465** habitus in dorsal view **466** median lobe of aedeagus in dorsal view **467** median lobe of aedeagus in lateral view **468** male tergite VIII **469** male sternite VIII **470** female tergite VIII **471** female sternite VIII **472** spermatheca. Scale bar of habitus = 1 mm; remaining scale bars = 0.2 mm.

Body length 2.5–2.7 mm, subparallel, dark brown with yellowish-brown legs and antennae (Fig. 465); integument moderately glossy, densely punctate and pubescent, pubescence short and adhering to body; head round, narrower than pronotum, eyes small, about one-quarter length of temples in dorsal view; antennal articles all elongate; pronotum round, about as wide as elytra; elytra slightly transverse, subquadrate; abdomen broadly arcuate laterally. **Male.** Median lobe of aedeagus with tubus broadening apicad in dorsal view (Fig. 466), bulbus with large carina, tubus long, slightly sinuate and produced ventrally at apex in lateral view (Fig. 467); tergite VIII rounded apically (Fig. 468); sternite VIII with apical margin broadly, triangularly produced in middle third, rounded at apex (Fig. 469). **Female.** Tergite VIII broadly rounded apically (Fig. 470); sternite VIII truncate apically (Fig. 471); spermatheca with capsule club shaped, duct U-shaped, with irregular tight coil posteriorly (Fig. 472).

######### Distribution.

Known only from NB, Canada.

######### Natural history.

Adults of *Oxypoda sunpokeana* were found in various wetland habitats. Specimens were collected by treading *Carex*, leather-leaf and sphagnum on a tamarack bog margin, sifted from litter at the base of a red maple in a *Carex* marsh, sifted from moist litter and grass on hummocks in an alder swamp and adjacent to a slow-flowing stream, sifted from leaf litter near the margin of a slow stream in a red maple swamp, sifted from moist grass litter on muddy soil along an abandoned (dried) beaver pond, and sifted from sphagnum hummocks with *Carex* and grasses in an open black spruce bog. Adults were collected during April, May, and July.

######### Comments.

This species is externally similar to *Oxypoda robusticornis* Bernhauer but has the median lobe of the aedeagus and spermatheca shaped differently. The only other Nearctic *Oxypoda* species with a similarly shaped median lobe is *Oxypoda subpolaris* Casey, but the latter has a differently shaped body with an enlarged, shield-shaped pronotum which is much broader than the elytra.

######## Parocyusa americana

Taxon classificationAnimaliaColeopteraStaphylinidae

(Casey, 1906)

Figs 473–476

Parocyusa americana (For comparison with Parocyusa fuliginosa (Casey), see Brunke et al. 2012.)

######### Material examined.

**New Brunswick, Carleton Co.**, Jackson Falls, 46.2257°N, 67.7437°W, 12.IX.2009, R.P. Webster, coll. // River margin near waterfall, splashing moss near splash zone of waterfall (1 ♀, RWC).

######### Natural history.

Brunke et al. (2012) reported specimens of this species from a hedgerow, from the bank of a sandy creek and from under a rock in a dry streambed. The specimen from NB was collected by splashing moss near the splash zone of a waterfall in September. Specimens from ON were collected during June, September, and October.

######### Distribution in Canada and Alaska.

ON, **NB** (Bousquet et al. 2013). Brunke et al. (2012) reported *Parocyusa americana* for the first time for Canada based on specimens from ON and suggested that this species might be more widespread in northeastern North America in habitats near running water.

######### Comments.

*Parocyusa americana* may be distinguished from *Parocyusa fuliginosa* by antennal articles VI-X elongate (subquadrate to transverse in *Parocyusa fuliginosa*), by elongate pronotum equal in length to elytra (pronotum shorter than elytra in *Parocyusa fuliginosa*), and by the shape of spermatheca. The male of *Parocyusa americana* is unknown.

**Figures 473–476.**
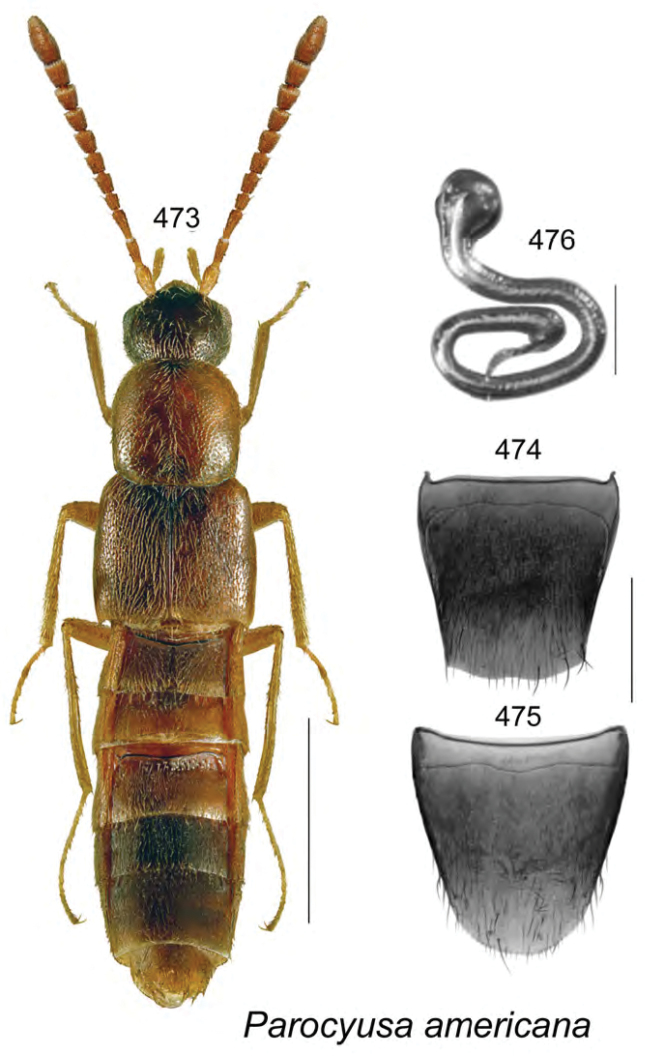
*Parocyusa americana* (Casey): **473** habitus in dorsal view **474** female tergite VIII **475** female sternite VIII **476** spermatheca. Scale bar of habitus = 1 mm; remaining scale bars = 0.2 mm.

######## Parocyusa fuliginosa

Taxon classificationAnimaliaColeopteraStaphylinidae

(Casey, 1906)

Figs 477–485

Parocyusa fuliginosa (For diagnosis, see Klimaszewski et al. 2011)

######### Material examined.

**New Brunswick, Restigouche Co.**, Pollard Brook at Pollard Rd., 47.9861°N, 67.6945°W, 31.VII.2012, R.P. Webster // Clear rocky stream, splashing gravel margin (1 ♂, RWC).

######### Natural history.

The only specimen of *Parocyusa fuliginosa* from NB was collected by splashing the gravel margin of a clear rocky stream in late July. In LB, adults were collected from rocks and gravel along a stream margin in early August.

######### Distribution in Canada and Alaska.

ON, **NB**, LB (Bousquet et al. 2013). Klimaszewski et al. (2011) reported this species for the first time for Canada from LB. Later, Brunke et al. (2012) reported an additional specimen from ON and suggested that this species might be more widespread in northeastern North America in habitats near running water.

**Figures 477–485.**
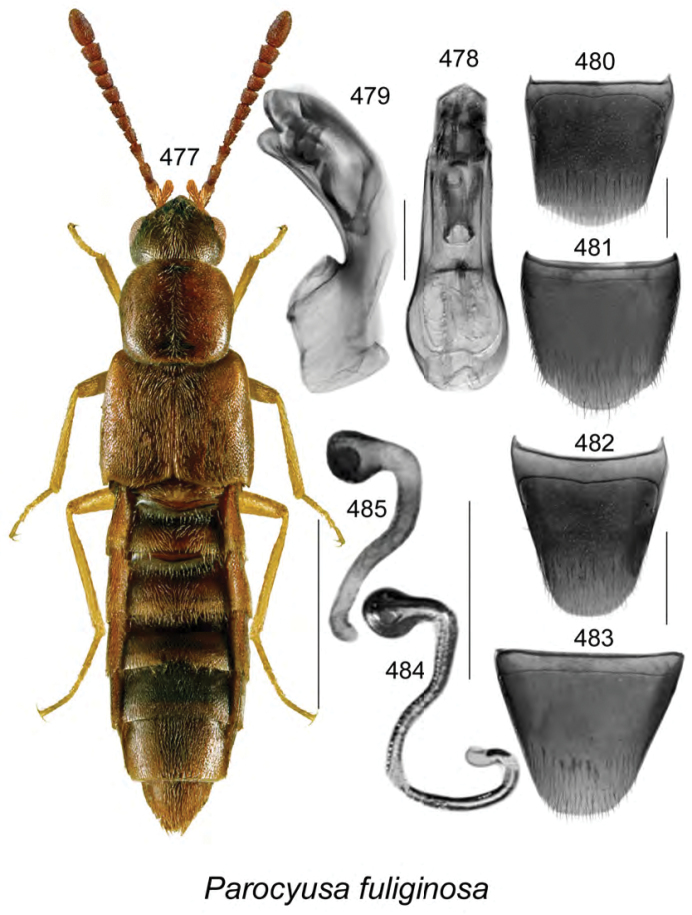
*Parocyusa fuliginosa* (Casey): **477** habitus in dorsal view **478** median lobe of aedeagus in dorsal view **479** median lobe of aedeagus in lateral view **480** male tergite VIII **481** male sternite VIII **482** female tergite VIII **483** female sternite VIII **484, 485**, spermatheca. Scale bar of habitus = 1 mm; remaining scale bars = 0.2 mm.

####### Subtribe Phloeoporina C.G. Thomson, 1859

######## Phloeopora canadensis

Taxon classificationAnimaliaColeopteraStaphylinidae

Klimaszewski & Langor, 2011

Figs 486–493

Phloeopora canadensis (For diagnosis, see Klimaszewski et al. 2011)

######### Material examined.

**New Brunswick, Northumberland Co.**, Upper Graham Plains, 47.1001°N, 66.8154°W, 28.V-10.VI.VII.2014, C. Alderson & V. Webster // Old black spruce forest, Lindgren funnel trap (1 ♂, RWC). **Restigouche Co.**, Dionne Brook P.N.A., 47.9064°N, 68.3441°W, 15–27.VI.2011, M. Roy & V. Webster // Old-growth white spruce & balsam fir forest, Lindgren funnel trap (3 ♀, RWC). **Sunbury Co.**, Sunpoke Lake, 45.7656°N, 66.5550°W, 20.VII-3.VIII.2012, C. Alderson & V. Webster // Red oak forest near seasonally flooded marsh, Lindgren funnel trap in canopy of *Quercus rubra* (1 ♀, RWC); Acadia Research Forest, 45.9990°N, 66.2623°W, 14–25.VI.2012, C. Alderson & V. Webster // Mature balsam fir forest with scattered red spruce & red maple, Lindgren funnel trap (1 ♀, RWC). **York Co.**, 14 km WSW of Tracy, S of Rt. 645, 45.6741°N, 66.8661°W, 16–30.VI.2010, R. Webster & C. MacKay, coll. // Old mixed forest with red & white spruce, red & white pine, balsam fir, eastern white cedar, red maple, and *Populus* sp., Lindgren funnel trap (2 ♂, RWC); Charters Settlement, 45.8286°N, 66.7365°W, 3.VI.2007, R.P. Webster, coll. // Mature red spruce forest, under bark of red spruce (1 sex undetermined, RWC); same data except 6.VI.2007 // Mature red spruce & red maple forest, under scolytid infested bark of red spruce (1 ♀, RWC); Douglas, Currie Mountain, 45.9832°N, 66.7564°W, 9–24.VII.2013, C. Alderson & V. Webster // Old *Pinus strobus* stand, Lindgren funnel trap 1 m high under *Pinus strobus* (1 ♂, RWC).

######### Natural history.

Most NB specimens of *Phloeopora canadensis* were captured in Lindgren funnel traps, mostly in conifer or mixed forests. These included an old black spruce forest, an old-growth white spruce and balsam fir forest, a mature red and white spruce forest, an old white pine stand, an old mixed forest and a red oak forest (adjacent to a black spruce stand). The only specimens with microhabitat data were collected from under bark of red spruce and under scolytid (*Dendroctonus*) infested bark of red spruce in a red spruce stand. Adults were collected from May to July. In NF, this species was collected in May, July, and October from under bark of tamarack recently killed by *Dendroctonus simplex* LeConte (Klimaszewski et al. 2011).

######### Distribution in Canada and Alaska.

QC, **NB**, NF (Bousquet et al. 2013).

**Figures 486–493.**
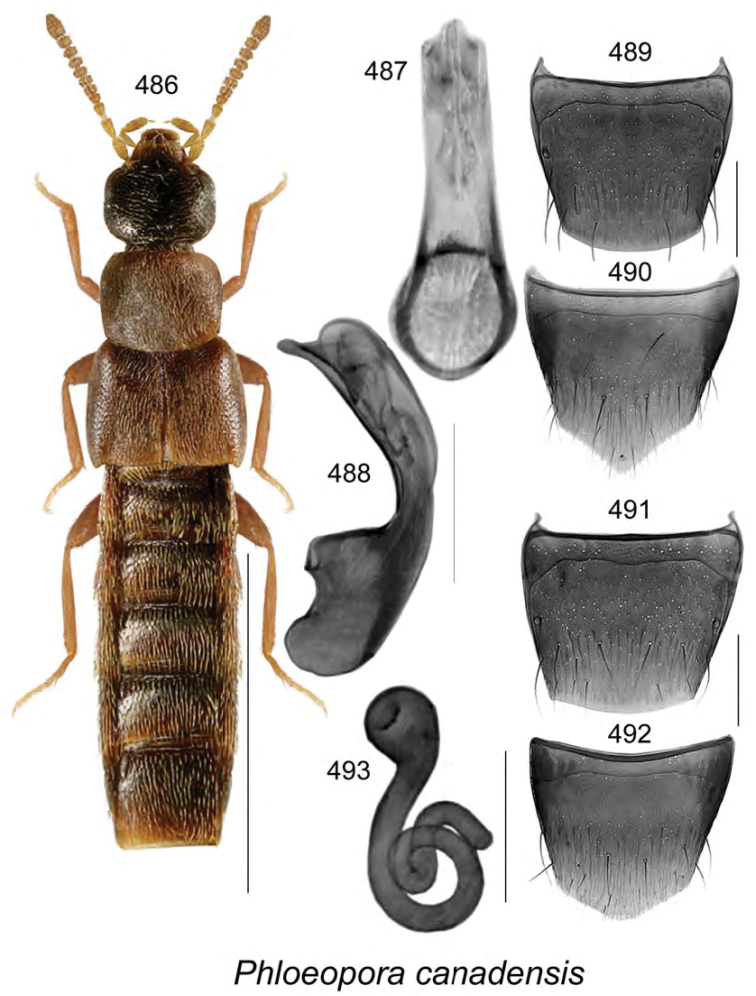
*Phloeopora canadensis* Klimaszewski & Langor: **486** habitus in dorsal view **487** median lobe of aedeagus in dorsal view **488** median lobe of aedeagus in lateral view **489** male tergite VIII **490** male sternite VIII **491** female tergite VIII **492** female sternite VIII **493** spermatheca. Scale bar of habitus = 1 mm; remaining scale bars = 0.2 mm.

######## Phloeopora gilbertae

Taxon classificationAnimaliaColeopteraStaphylinidae

Klimaszewski & Webster sp. n.

http://zoobank.org/59A46EAB-8630-4A35-AA56-945C45A2CCD1

Figs 494–500

######### Holotype (male).

**Canada, New Brunswick, Sunbury Co.**, Gilbert Island, 45.8770°N, 66.2954°W. 18–28.V.2012, C. Alderson & V. Webster // Hardwood forest, Lindgren funnel trap in canopy of *Juglans cinerea* (LFC). **Paratypes: Canada, New Brunswick, Gloucester Co.**, Bathurst, Daly Point Nature Preserve, 47.6392°N, 65.6098°W, 13–28.V.2015, C. Alderson & V. Webster // Mixed forest, purple Lindgren funnel trap in canopy (1, AFC); same data but 25.VI-9.VII.2015, purple Lindgren funnel trap in canopy (1, AFC). **Northumberland Co.**, ca. 1.5 km NW of Sevogle, 47.0939°N, 65.8387°W, 11–26.VI.2013, C. Alderson & V. Webster // *Populus tremuloides* stand with a few conifers, Lindgren funnel trap in canopy of *Populus tremuloides* (1 sex undetermined, RWC); ca. 2.5 km NW of Sevogle, 47.0876°N, 65.8613°W, 26.VI-8.VII.2013, C. Alderson & V. Webster // *Pinus banksiana* forest, Lindgren funnel trap (1 ♀, RWC). **Queens Co.**, Cranberry Lake P.N.A., 46.1125°N, 65.6075°W, 25.V-7.VI.2011, M. Roy & V. Webster // Red oak forest, Lindgren funnel trap (1 ♂, RWC). **Restigouche Co.**, Jacquet River Gorge P.N.A., 47.8257°N, 66.0764°W, 29.V-10.VI.2014, C. Alderson & V. Webster // Old *Populus balsamifera* stand near river, Lindgren funnel traps under trees (2 sex undetermined, AFC; 1 ♀, RWC). **Sunbury Co.**, Gilbert Island, 45.8770°N, 66.2954°W, 18–28.V.2012, C. Alderson, C. Hughes, & V. Webster // Hardwood forest, Lindgren funnel trap 1 m high under *Tilia americana* (1 ♂, RWC); same data except 28.V-12.VI.2012 // Lindgren funnel trap in canopy of *Juglans cinerea* (1 sex undetermined, LFC; 2 ♂, 1 ♀, RWC); same data except 29.VI-11.VII.2012 (1 ♂, LFC; 1 ♂, RWC); same data except 20.VI-5.VII.2013 // Lindgren funnel trap in canopy of *Populus tremuloides* (1 ♂, RWC). **York Co.**, 16 km W of Tracy, off Rt. 645, 45.6855°N, 66.8847°W, 18.V-2.VI.2010, R. Webster & C. MacKay, coll. // Old red pine forest, Lindgren funnel trap (1 ♀, LFC); Fredericton, Odell Park, 45.9539°N, 66.6666°W, 2–15.V.2013, C. Alderson & V. Webster // Hardwood stand, Lindgren funnel trap 1 m high under trees (1 ♂, RWC). **Northwest Territories**, vic. Inuvik, 5 km SE townsite, 68.32881°N, 133.63556°W, 17.VII-3.VIII.2001 mixed *Picea*–*Betula* forest // UHR ethanol funnel trap 1c, M. Gavel et al., collectors (1 ♂, LFC).

######### Etymology.

This species is dedicated to Amélie Gilbert (LFC), who dissected thousands of specimens of Aleocharinae for our projects.

######### Description.

**Figures 494–500.**
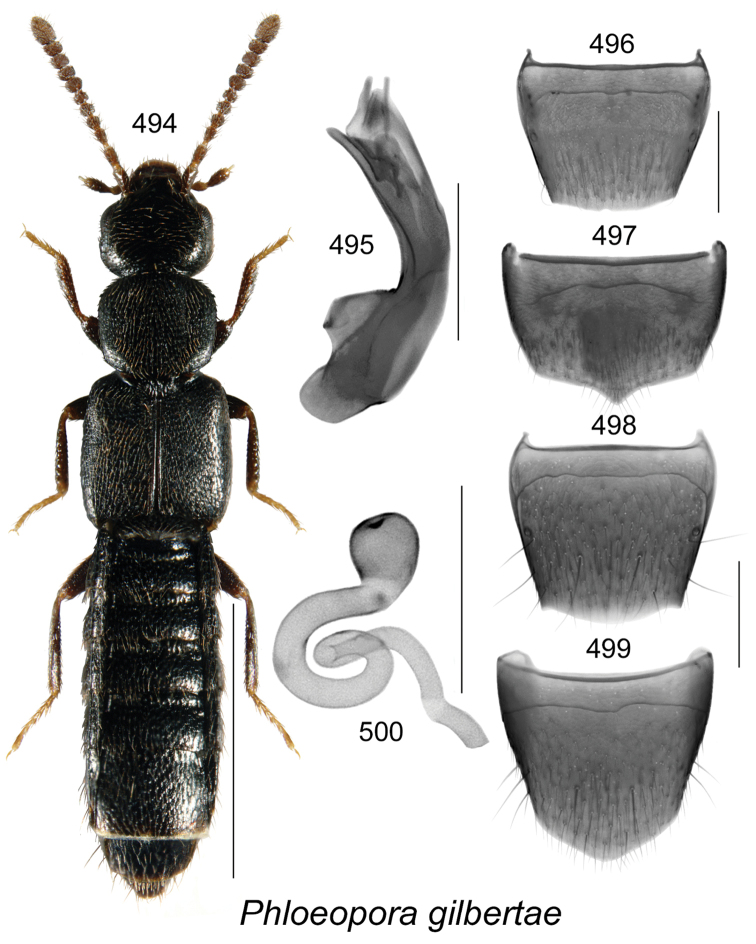
*Phloeopora gilbertae* Klimaszewski & Webster: **494** habitus in dorsal view **495** median lobe of aedeagus in lateral view **496** male tergite VIII **497** male sternite VIII **498** female tergite VIII **499** female sternite VIII **500** spermatheca. Scale bar of habitus = 1 mm; remaining scale bars = 0.2 mm.

Body length 2.2–2.4 mm, narrowly elongate, subparallel, black except tarsi and antennae reddish brown (Fig. 494); integument strongly glossy, moderately punctate and pubescent, pubescence short and adhering to body; head subequal to pronotum in size, pubescence directed outward and posterolaterad from midline of disk; eyes large, slightly shorter than postocular area; antennal articles incrassate and articles V–X moderately to strongly transverse; pronotum trapezoidal in shape, broadest subapically, narrower than elytra, pubescence directed almost straight posteriad; elytra elongate, with distinct narrowly rounded shoulders, pubescence directed straight and obliquely posteriad; abdomen subparallel with three basal tergites deeply impressed basally. **Male.** Median lobe of aedeagus with tubus long, narrow and curved ventrally (Fig. 495); tergite VIII truncate apically (Fig. 496); sternite VIII with apical margin obtusely triangularly produced in middle one-sixth (Fig. 497). **Female.** Tergite VIII broadly arcuate apically (Fig. 498); sternite VIIII with apical margin broadly triangularly produced, apex rounded (Fig. 499); spermatheca with capsule spherical, stem forming loose coil in apical half, sinuous and narrower basad (Fig. 500).

######### Distribution.

This species is known from NB and the NT and is likely transcontinental in Canada.

######### Natural history.

All adults of *Phloeopora gilbertae* from NB were captured in Lindgren funnel traps, most in hardwood forests. Specimens were captured in the canopy of a *Populus tremuloides* Michx. (trembling aspen) stand, in the canopy of *Populus tremuloides*, *Juglans cinerea* L. (butternut) and under *Tilia americana* L. (American basswood) in a hardwood forest on an island in a river, in Lindgren traps under *Populus balsamifera* L. in a *Populus balsamifera* stand near a river, in a red oak stand with *Populus*, a hardwood stand, and in a *Pinus banksiana* forest. The specimen from the Northwest Territories was collected in a Lindgren funnel trap in a mixed *Picea*–*Betula* forest. Adults were collected during May, June, July, and August. Other members of this genus live in subcortical habitats (Klimaszewski et al. 2011, Webster et al. 2012), and we presume this species lives in similar habitats.

######### Comments.

This species may be separated from its Nearctic congeners by its body proportions, the uniformly black body color except for the appendages, and by the shape of the median lobe of the aedeagus in lateral view, and the spermatheca. It differs from the other two eastern Canadian species, *Phloeopora oregona* Casey and *Phloeopora canadensis* Klimaszewski and Langor by the black body (brown with darker head in *Phloeopora canadensis* and *Phloeopora oregona*), the elongate rather than transverse elytra, the apical part of the median lobe of the tubus straight in lateral view (strongly produced in the other two species), the spermathecal stem forming a shorter loop, and male tergite VIII with the apex more abruptly produced than that of *Phloeopora canadensis*. For illustrations of *Phloeopora canadensis*, see Klimaszewski et al. 2011.

## Supplementary Material

XML Treatment for Aleochara (Calochara) rubricalis

XML Treatment for Aleochara (Calochara) speculicollis

XML Treatment for Aleochara (Echochara) ocularis

XML Treatment for Tinotus caviceps

XML Treatment for Acrotona brachyoptera

XML Treatment for Acrotona sphagnorum

XML Treatment for Acrotona subpygmaea

XML Treatment for Alevonota gracilenta

XML Treatment for Atheta (Datomicra) whitehorsensis

XML Treatment for Atheta (Dimetrota) alphacrenuliventris

XML Treatment for Atheta (Dimetrota) bubo

XML Treatment for Atheta (Dimetrota) campbelli

XML Treatment for Atheta (Dimetrota) chartersensis

XML Treatment for Atheta (Dimetrota) cranberriensis

XML Treatment for Atheta (Dimetrota) giguereae

XML Treatment for Atheta (Dimetrota) makepeacei

XML Treatment for Atheta (Dimetrota) mcalpinei

XML Treatment for Atheta (Dimetrota) petitcapensis

XML Treatment for Atheta (Dimetrota) sphagnicola

XML Treatment for Atheta (sensu lato) pseudoschistoglossa

XML Treatment for Atheta (sensu lato) thujae

XML Treatment for Atheta (Pseudota) klagesi

XML Treatment for Atheta (Pseudota) pseudoklagesi

XML Treatment for Dinaraea curtipenis

XML Treatment for Dinaraea longipenis

XML Treatment for Dinaraea subdepressa

XML Treatment for Paragoniusa myrmicae

XML Treatment for Philhygra atypicalis

XML Treatment for Philhygra hygrotopora

XML Treatment for Philhygra larsoni

XML Treatment for Philhygra proterminalis

XML Treatment for Philhygra pseudolarsoni

XML Treatment for Philhygra terrestris

XML Treatment for Schistoglossa (Schistoglossa) pelletieri

XML Treatment for Seeversiella globicollis

XML Treatment for Strigota ambigua

XML Treatment for Strigota obscurata

XML Treatment for Trichiusa hirsuta

XML Treatment for Trichiusa pilosa

XML Treatment for Trichiusa robustula

XML Treatment for Thamiaraea claydeni

XML Treatment for Thamiaraea corverae

XML Treatment for Myrmecopora vaga

XML Treatment for Pleurotobia bourdonae

XML Treatment for Pleurotobia brunswickensis

XML Treatment for Agaricomorpha vincenti

XML Treatment for Gyrophaena (Gyrophaena) aldersonae

XML Treatment for Gyrophaena (Gyrophaena) brevicollis

XML Treatment for Anomognathus americanus

XML Treatment for Hoplandria (Lophomucter) laevicollis

XML Treatment for Oligota chrysopyga

XML Treatment for Oligota parva

XML Treatment for Oligota polyporicola

XML Treatment for Oligota pusillima

XML Treatment for Oligota sevogle

XML Treatment for Blepharhymenus brendeli

XML Treatment for Meotica pallens

XML Treatment for Calodera caseyi

XML Treatment for Dexiogyia angustiventris

XML Treatment for Hylota cryptica

XML Treatment for Mniusa

XML Treatment for Mniusa minutissima

XML Treatment for Mniusa odelli

XML Treatment for Neothetalia canadiana

XML Treatment for Oxypoda sunpokeana

XML Treatment for Parocyusa americana

XML Treatment for Parocyusa fuliginosa

XML Treatment for Phloeopora canadensis

XML Treatment for Phloeopora gilbertae

## References

[B1] Ahn K-J Ashe JS (1995) Systematic position of the intertidal genus *Bryobiota* Casey and a revised phylogeny of the falagriine genera of America north of Mexico (Coleoptera: Staphylinidae). Annals of the Entomological Society of America 88(2): 143–154. doi: 10.1093/aesa/88.2.143

[B2] Ashe JS (1990) Natural history, development and immatures of *Pleurotobia tristigmata* (Erichson) (Coleoptera: Staphylinidae: Aleocharinae). The Coleopterists Bulletin 44(4): 445–460.

[B3] Ashe JS (1992) Phylogeny and revision of genera of the subtribe Bolitocharina (Coleoptera: Staphylinidae; Aleocharinae). The University of Kansas Science Bulletin 54(10): 335–406.

[B4] Ashe JS (2000) Aleocharinae. In: Arnett RH Thomas MC (Eds) American Beetles, Volume 1: Archostemata, Myxophaga, Adephaga, Polyphaga: Staphyliniformia. CRC Press, Boca Raton, 299–319, 358–374.

[B5] Assing V (1995) Ernstnachweis von *Oligota inexpectata* Williams für Deutschland, mit Bemerkungen zur Unterscheidung von *O. pusillima* (Gravenhorst) und *O. pumilio* Kiesenwetter (Col., Staphylinidae). Entomologische Nachrichten und Berichte 39(4): 224–226.

[B6] Assing V (1997) A revision of the Western Palaearctic species of *Myrmecopora* Saulcy, 1864, *senso lato* and *Eccoptoglossa* Luze, 1904 (Coleoptera, Staphylinidae, Aleocharinae, Falagriini). Beiträge zur Entomologie 47(1): 69–151.

[B7] Assing V (2002) A taxonomic and phylogenetic revision of *Amarochara* Thomson. I. The species of the Holarctic region (Coleoptera: Staphylinidae, Aleocharinae, Oxypodini). Beiträge zur Entomologie 52(1): 111–204.

[B8] Assing V (2003) A new species of *Oligota* from Morocco, with redescriptions of *O. tugurtana* Fauvel and *O. pilicornis* Fauvel (Coleoptera: Staphylinidae, Aleocharinae). Linzer biologische Beiträge 35(1): 533–537.

[B9] Assing V (2008) The genus *Calodera* Mannerheim in Canada (Insecta, Coleoptera, Staphylinidae, Aleocharinae). ZooKeys 2: 203–208. doi: 10.3897/zookeys.2.6

[B10] Assing V Wunderle P (2008) On the *Alevonota* species of the western Palaearctic region (Coleoptera: Staphylinidae: Aleocharinae: Athetini). Beiträge zur Entomologie 58(1): 145–189.

[B11] Bernhauer M (1907) Neue Aleocharini aus Nordamerika. (Col.) (3. Stück.). Deutsche Entomologische Zeitschrift 1907(4): 381–405.

[B12] Bernhauer M (1909) Neue Aleocharini aus Nordamerika. (Col.) (4. Stück.). Deutsche Entomologische Zeitschrift 1909(4): 515–528.

[B13] Bousquet Y Bouchard P Davies AE Sikes D (2013) Checklist of beetles (Coleoptera) of Canada and Alaska. Pensoft Series Faunistica No. 109, Sofia-Moscow, 402 pp. 10.3897/zookeys.360.4742 PMC3867111 24363590

[B14] Brunke AJ (2011, unpubl.) Diversity, habitat use and potential biocontrol services of rove beetles (Coleoptera: Staphylinidae) in soybean agroecosystems and adjacent hedgerows. MSc thesis, Environmental Biology, University of Guelph, xv + 285 pp.

[B15] Brunke AJ Klimaszewski J Dorval J-A Bourdon C Paiero SM Marshall SA (2012) New species and distributional records of Aleocharinae (Coleoptera, Staphylinidae) from Ontario, Canada, with a checklist of recorded species. ZooKeys 186: 119–206. doi: 10.3897/zookeys.186.2947 22577320 10.3897/zookeys.186.2947 PMC3349194

[B16] Casey TL (1894) [1893] Coleopterological notices. V. Annals of the New York Academy of Sciences 7(6/12): 281–606, pl. 1. [the actual year of publication was 1894 but it is cited often as 1893]

[B17] Casey TL (1906) Observation on the Staphylinid groups Aleocharinae and Xantholinini, chiefly of America. Transactions of the American Society of St. Louis 16: 125–434.

[B18] Fauvel A (1889) Liste des coléoptères communs à l’Europe et à l’Amérique du Nord. D’après le catalogue de M. J. Hamilton. Avec remarques et additions. Revue d’Entomologie 8: 92–174.

[B19] Fenyes A (1918) Coleoptera. Fam. Staphylinidae, subfamily Aleocharinae. Genera Insectorum 173: 1–110.

[B20] Frank JH (1972) The genus *Oligota* Mannerheim in the Caribbean region (Coleoptera: Staphylinidae). The Coleopterists Bulletin 26(4): 125–146.

[B21] Frank JH (1976) New records of *Oligota* Mannerheim (Staphylinidae) in Florida. The Coleopterists Bulletin 29 [1975](4): 279–280.

[B22] Frank JH Bennett FD Cromroy HL (1992) Distribution and prey records for *Oligota minuta* (Coleoptera: Staphylinidae), a predator of mites. The Florida Entomologist 75(3): 376–380. doi: 10.2307/3495859

[B23] Génier F (1989) A revision of the genus *Hoplandria* Kraatz of America north of Mexico (Coleoptera: Staphylinidae, Aleocharinae). Memoirs of the Entomological Society of Canada No. 150: 3–59.

[B24] Gusarov VI (2003a) Revision of some types of North American aleocharinaes (Coleoptera: Staphylinidae: Aleocharinae) with synonymic notes. ZooTaxa 353: 1–134.

[B25] Gusarov VI (2003b) A revision of the genus *Seeversiella* Ashe 1986 (Coleoptera: Staphylinidae: Aleocharinae). Zootaxa 142: 1–102.

[B26] Hoebeke ER (1988) Review of the genus *Thamiaraea* Thomson in North America (Coleoptera: Staphylinidae: Aleocharinae) with description of a new species. Journal of the New York Entomological Society 96(1): 16–25.

[B27] Hoebeke ER (1994) *Thamiaraea paralira*, a new species from North America, and new distributional and habitat data for other Nearctic species of *Thamiaraea* (Coleoptera, Staphylinidae). Proceedings of the Entomological Society of Washington 96(1): 1–7.

[B28] Hughes CC Johns RC Sweeney JD (2014) A technical guide to installing beetle traps in the upper crown of trees. Journal of the Acadian Entomological Society 10: 12–18.

[B29] Klimaszewski J (1984) A revision of the genus *Aleochara* Gravenhorst of America north of Mexico (Coleoptera: Staphylinidae, Aleocharinae). Memoirs of the Entomological Society of Canada No. 129: 1–211. doi: 10.4039/entm116129fv

[B30] Klimaszewski J Peck SB (1986) A review of the cavernicolous Staphylinidae (Coleoptera) of eastern North America: Part I. Aleocharinae. Quaestiones Entomologicae 22(2): 51–113.

[B31] Klimaszewski J Pelletier G Sweeney J (2002) Genus *Tinotus* (Coleoptera: Staphylinidae, Aleocharinae) from America north of Mexico: review of the types, distribution records, and key to species. The Canadian Entomologist 134(3): 281–298. doi: 10.4039/Ent134281-3

[B32] Klimaszewski J Pelletier G (2004) Review of the *Ocalea* group of genera (Coleoptera, Staphylinidae, Aleocharinae) in Canada and Alaska: new taxa, bionomics, and distribution. The Canadian Entomologist 136(4): 443–500. doi: 10.4039/n03-069

[B33] Klimaszewski J Sweeney J Price J Pelletier G (2005) Rove beetles (Coleoptera: Staphylinidae) in red spruce stands, eastern Canada: diversity, abundance, and descriptions of new species. The Canadian Entomologist 137(1): 1–48. doi: 10.4039/n03-123

[B34] Klimaszewski J Assing V Majka CG Pelletier G Webster RP Langor DW (2007) Records of adventive aleocharine beetles (Coleoptera: Staphylinidae: Aleocharinae) found in Canada. The Canadian Entomologist 139(1): 54–79. doi: 10.4039/n05-105

[B35] Klimaszewski J Webster RP Savard K (2009a) First record of the genus *Schistoglossa* Kraatz from Canada with descriptions of seven new species (Coleoptera, Staphylinidae, Aleocharinae). In: Majka CG Klimaszewski J (Eds) Biodiversity, Biosystematics, and Ecology of Canadian Coleoptera II. ZooKeys 22: 45–79. doi: 10.3897/zookeys.22.153

[B36] Klimaszewski J Webster RP Savard K (2009b) Review of the rove beetle species of the subtribe Gyrophaenina Kraatz (Coleoptera, Staphylinidae) from New Brunswick, Canada: new species, provincial records and bionomic information. In: Majka CG Klimaszewski J (Eds) Biodiversity, Biosystematics, and Ecology of Canadian Coleoptera II. ZooKeys 22: 81–170. doi: 10.3897/zookeys.22.219

[B37] Klimaszewski J Langor D Pelletier G Bourdon C Perdereau L (2011) Aleocharine beetles (Coleoptera, Staphylinidae) of the province of Newfoundland and Labrador, Canada. Pensoft Publishers, Sofia and Moscow, 313 pp.

[B38] Klimaszewski J Godin B Bourdon C (2012) Further contributions to the aleocharine fauna of the Yukon Territory, Canada (Coleoptera, Staphylinidae). ZooKeys 186: 207–237. doi: 10.3897/zookeys.186.2674 22577321 10.3897/zookeys.186.2674 PMC3349195

[B39] Klimaszewski J Brunke A Assing V Langor DW Newton AF Bourdon C Pelletier G Webster RP Herman L Perdereau L Davies A Smetana A Chandler DS Majka C Scudder GR (2013a) Synopsis of adventive species of Coleoptera (Insecta) recorded from Canada. Part 2: Staphylinidae. Pensoft, Sofia–Moscow, 360 pp.

[B40] Klimaszewski J Webster RP Langor DW Bourdon C Jacobs J (2013b) Review of Canadian species of the genus *Dinaraea* Thomson, with descriptions of six new species (Coleoptera, Staphylinidae, Aleocharinae, Athetini). ZooKeys 327: 65–101. doi: 10.3897/zookeys.327.5908 24167422 10.3897/zookeys.327.5908 PMC3804766

[B41] Klimaszewski J Webster RP Langor DW Bourdon C Hammond HEJ Pohl GR Godin B (2014) Review of Canadian species of the genera *Gnathusa* Fenyes, *Mniusa* Mulsant & Rey and *Ocyusa* Kraatz (Coleoptera, Staphylinidae, Aleocharinae). ZooKeys 412: 9–40. doi: 10.3897/zookeys.412.7282 24899860 10.3897/zookeys.412.7282 PMC4042694

[B42] Klimaszewski J Godin B Langor D Bourdon C Lee S-I Horwood D (2015a) New distribution records for Canadian Aleocharinae (Coleoptera, Staphylinidae), and new synonymies for *Trichiusa*. ZooKeys 498: 51–91. doi: 10.3897/zookeys.498.9282 25931964 10.3897/zookeys.498.9282 PMC4410149

[B43] Klimaszewski J Webster RP Bourdon C Pelletier G Godin B Langor DW (2015b) Review of Canadian species of the genus *Mocyta* Mulsant & Rey (Coleoptera, Staphylinidae, Aleocharinae), with the description of a new species and a new synonymy. ZooKeys 487: 111–139. doi: 10.3897/zookeys.487.9151 25829852 10.3897/zookeys.487.9151 PMC4366688

[B44] Klimaszewski J Webster RP Sikes D Bourdon C Labrecque M (2015c) A review of Canadian and Alaskan species of the genera *Clusiota* Casey and Atheta Thomson, subgenus Microdota Mulsant & Rey (Coleoptera, Staphylinidae, Aleocharinae). ZooKeys 524: 103–136. doi: 10.3897/zookeys.524.6105 26478708 10.3897/zookeys.524.6105 PMC4602293

[B45] Lindgren BS (1983) A multiple funnel trap for scolytid beetles (Coleoptera). The Canadian Entomologist 115(3): 299–302. doi: 10.4039/Ent115299-3

[B46] Lohse GA (1974) Tribe Gyrophaenini. In: Freude H Harde KW Lohse GA (Eds) Die Käfer Mitteleuropas. Band 5. Staphylinidae II (Hypocyphtinae und Aleocharinae), Pselaphidae. Goecke & Evers, Krefeld, 25–34.

[B47] Lohse GA Klimaszewski J Smetana A (1990) Revision of Arctic Aleocharinae of North America (Coleoptera: Staphylinidae). The Coleopterists Bulletin 44(2): 121–202.

[B48] Majka CG Klimaszewski J Lauff RF (2008a) The coastal rove beetles (Coleoptera, Staphylinidae) of Atlantic Canada: a survey and new records. In: Majka CG Klimaszewski J (Eds) Biodiversity, Biosystematics, and Ecology of Canadian Coleoptera. ZooKeys 2: 115–150. doi: 10.3897/zookeys.2.2

[B49] Majka CG Klimaszewski J (2008b) New records of Canadian Aleocharinae (Coleoptera, Staphylinidae). ZooKeys 2: 85–114. doi: 10.3897/zookeys.2.7 10.3897/zookeys.498.9282 PMC4410149 25931964

[B50] Majka CG Klimaszewski J (2010) Contributions to the knowledge of the Aleocharinae (Coleoptera, Staphylinidae) in the Maritime Provinces of Canada. ZooKeys 46: 15–39. doi: 10.3897/zookeys.46.413

[B51] Maruyama M Klimaszewski J (2004) A new genus and species of the myrmecophilous Athetini, *Paragoniusa myrmicae* (Coleoptera: Staphylinidae: Aleocharinae) from Canada. Entomological Review of Japan 59: 241–248.

[B52] Maruyama M Klimaszewski J (2006) Notes on myrmecophilous aleocharines (Insecta, Coleoptera, Staphylinidae) from Canada, with a description of a new species of *Myrmoecia*. Bulletin of the National Science Museum of Tokyo, Ser. A 32(3): 125–131.

[B53] Seevers CH (1951) A revision of the North American and European staphylinid beetles of the subtribe Gyrophaenae (Aleocharinae, Bolitocharini). Fieldiana: Zoology 32(10): 657–762. doi: 10.5962/bhl.title.2816

[B54] Seevers CH (1978) A generic and tribal revision of the North American Aleocharinae (Coleoptera: Staphylinidae) [with additions and annotations by Lee H. Herman]. Fieldiana: Zoology 71: i–vi, 1–289.

[B55] Smetana A (2004) Subfamily Aleocharinae Fleming, 1921. In: Löbl I Smetana A (Eds) Catalogue of Palaearctic Coleoptera, Vol. 2. Apollo Books, Stenstrup, 353–494.

[B56] Strand A Vik A (1964) Die Genitalorgane der nordischen Arten der Gattung *Atheta* Thoms. (Col., Staphylinidae). Norsk Entomologisk Tidsskrift 12(5-8): 327–335, Taf. I-XXI.

[B57] Webster RP Klimaszewski J Pelletier G Savard K (2009) New Staphylinidae (Coleoptera) records with new collection data from New Brunswick, Canada. 1. Aleocharinae. In: Majka CG Klimaszewski J (Eds) Biodiversity, Biosystematics, and Ecology of Canadian Coleoptera II. ZooKeys 22: 171–248. doi: 10.3897/zookeys.22.152

[B58] Webster RP Klimaszewski J Sweeney JD DeMerchant I (2012) New Staphylinidae (Coleoptera) records with new collection data from New Brunswick, and an addition to the fauna of Quebec, Canada: Aleocharinae. In: Klimaszewski J Anderson R (Eds) Biosystematics and Ecology of Canadian Staphylinidae (Coleoptera) II. ZooKeys 186: 83–118. doi: 10.3897/zookeys.186.2655 22577319 10.3897/zookeys.186.2655 PMC3349193

[B59] Williams SA (1970a) Notes on the genus *Oligota* Mannerheim (Col., Staphylinidae) and a key to the British species. The Entomologist’s Monthly Magazine 106: 54–62.

[B60] Williams SA (1970b) Notes on the genus *Oligota* (2): a European species new to science and the distribution of *O. muensteri* Bernh. (Col., Staphylinidae). The Entomologist’s Monthly Magazine 106: 109–110.

[B61] Williams SA (1972) A Brazilian species of *Oligota* (Col., Staphylinidae) new to science and imported into Britain. The Entomologist’s Monthly Magazine 108: 38–39.

[B62] Williams SA (1973a) Further notes on the genus *Oligota* (Col., Staphylinidae). The Entomologist’s Monthly Magazine 108: 107–109.

[B63] Williams SA (1973b) The genus *Oligota* Mannerheim (Col., Staphylinidae) in the Canary Islands. The Entomologist’s Monthly Magazine 108: 222–229.

[B64] Williams SA (1975) The *Oligota* (Col., Staphylinidae) of Madeira. Boletim do Museu Municipal do Funchal. No. XXIX. 128: 18–25.

[B65] Williams SA (1976) The genus *Oligota* (Coleoptera: Staphylinidae) in New Zealand. New Zealand Journal of Zoology 3: 247–255. doi: 10.1080/03014223.1976.9517914

[B66] Williams SA (1979) The genus *Oligota* Mannerheim (Col., Staphylinidae) in the Ethiopian region. The Entomologist’s Monthly Magazine 114: 177–190.

